# Synthesis and Antinociceptive Effect of Some Thiazole-Piperazine Derivatives: Involvement of Opioidergic System in the Activity

**DOI:** 10.3390/molecules26113350

**Published:** 2021-06-02

**Authors:** Nazlı Turan Yücel, Derya Osmaniye, Ümmühan Kandemir, Asaf Evrim Evren, Özgür Devrim Can, Ümide Demir Özkay

**Affiliations:** 1 Faculty of Pharmacy, Department of Pharmacology, Anadolu University, Eskişehir 26470, Turkey; ozgurdt@anadolu.edu.tr (Ö.D.C.); udemir@anadolu.edu.tr (Ü.D.Ö.); 2 Faculty of Pharmacy, Department of Pharmaceutical Chemistry, Anadolu University, Eskişehir 26470, Turkey; dosmaniye@anadolu.edu.tr; 3 Institute of Health Sciences, Department of Pharmacology, Anadolu University, Eskişehir 26470, Turkey; ummuhan_kandemir@hotmail.com; 4 Vocational School of Health Services, Pharmacy Services, Bilecik Şeyh Edebali University, Bilecik 11230, Turkey; asafevrim.evren@bilecik.edu.tr

**Keywords:** thiazole, piperazine, tail-clip, hot-plate, acetic acid-induced writhing test, opioid

## Abstract

In this study, we aimed to design and synthesize novel molecules carrying both the thiazole and piperazine rings in their structures and to investigate their antinociceptive activity. Targeted compounds were obtained by reacting thiosemicarbazide derivative and appropriate 2-bromoacetophenone in ethanol. The structures of the obtained compounds were determined using data from various spectroscopic methods (IR, ^1^H-NMR, ^13^C-NMR, and LCMSMS). Experimental data from in vivo tests showed that test compounds **3a**–**3c**, **3f**, and **3g** (50 mg/kg) significantly prolonged reaction times of animals in tail-clip and hot-plate tests compared to the controls, indicating that these compounds possess centrally mediated antinociceptive activities. Furthermore, these compounds reduced the number of writhing behaviors in the acetic acid-induced writhing tests, showing that the compounds also possess peripheral antinociceptive activity. In the mechanistic studies, naloxone pre-treatments abolished the antinociceptive activities of compounds **3a**–**3c**, **3f**, and **3g**, indicating that opioidergic mechanisms were involved in their antinociceptive effects. Molecular docking studies demonstrating significant interactions between the active compounds and µ- and δ-opioid receptor proteins supported the pharmacological findings. This study is the first showing that molecules designed to bear thiazole and piperazine moieties together on their structure exert centrally and peripherally mediated antinociceptive effects by activating the opioid system.

## 1. Introduction

Pain is a health problem affecting the quality of life of patients due to its prevalence and accompanying disabilities. The pharmacological agents used in the treatment of pain include nonsteroidal anti-inflammatory drugs (NSAID), opioid analgesics, and analgesic adjuvants (such as antidepressants and local anesthetics) [1]. Although there are various analgesics used in clinics today, pain management is still a challenge due to concomitant undesirable side effects of these drugs. Long-term NSAID intake increases the risk of gastrointestinal complications, renal damage, and cardiovascular effects [2], while currently used opioid analgesics have negative effects such as sedation, respiratory depression, addiction, and tolerance [3]. Therefore, studies on the discovery and development of safer alternative drugs with comparable or better analgesic efficacy than conventional drugs are ongoing.

Nitrogen- and sulfur-containing heterocycles are frequently used in drug synthesis. Thiazole, a five-membered ring system carrying three carbons, one nitrogen, and one sulfur atom, is one such structure. It has been reported that thiazole-bearing compounds have some central nervous system (CNS)-related effects, such as anti-schizophrenic [4], anti-parkinsonian [5], neuroprotective [6], acetylcholinesterase inhibitory [7], anticonvulsant [8], antidepressant [9], and sedative-hypnotic [10] effects. Another heterocyclic structure, piperazine, is a 6-membered saturated ring system containing two nitrogen atoms in the first and fourth positions. Piperazine structure is present in several currently used CNS-related drugs, such as amoxapine, trazodone, hydroxyzine, buspirone, clozapine, aripiprazole [11], and vortioxetine [12].

A considerable amount of research data have been reported on the analgesic and anti-inflammatory effects of compounds carrying thiazole [13,14,15,16,17,18,19,20,21] or piperazine moieties [22,23,24,25,26,27,28,29,30,31]. Inhibition of COX isoenzymes [13,16], modulation of glutamatergic system through metabotropic and ionotropic (NMDA) receptors [20], inhibition of cytokine (TNF-α and IL-1β) signaling [20], involvement of α_2_ adrenergic, adenosinergic, and D_2/3_ dopaminergic receptors [22,23], blockage of T-type calcium channels [24], participation of 5-HT_1A_ and 5-HT_2A_ serotonergic receptors [25,26], and contribution of opioid system [21,30,31] have been suggested as some possible mechanisms underlying the aforementioned analgesic effects. 

**Figure 1:**
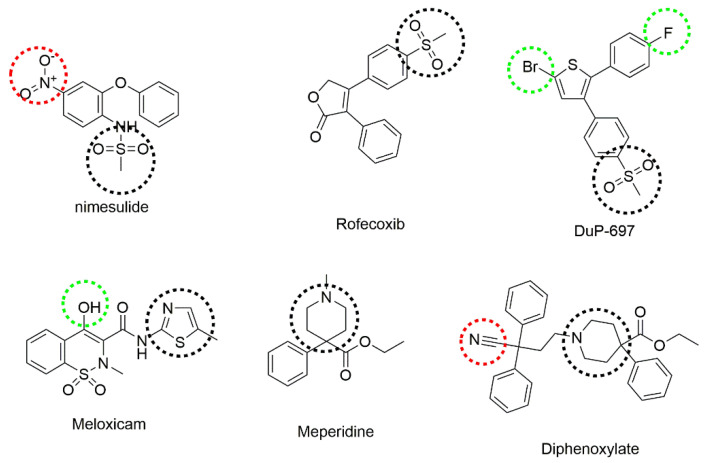
Some analgesic agents containing thiazole, secondary amine, or methylsulfonyl groups. Thiazole, secondary amine, or methylsulfonyl groups are marked in black, electron-donating substituents are marked with green, and electron-withdrawing substituents are marked with red.

The chemical structures of some analgesic agents that contain thiazole, secondary amine, or methylsulfonyl groups, similar to our test compounds, are provided in Figure 1.

Based on the antinociceptive activities of thiazole and piperazine ring systems, we designed and synthesized eight novel compounds containing both moieties and investigated their possible antinociceptive activities by well-validated in vivo methods. In addition, studies on the mechanism of action were performed with naloxone, a non-selective opioid receptor antagonist, to examine the possible involvement of the opioidergic mechanisms in the activity. Binding properties of test compounds to µ-, δ-, and ĸ-opioid receptors were evaluated by in silico studies.

## 2. Results

### 2.1. Chemistry

**Figure 2:**
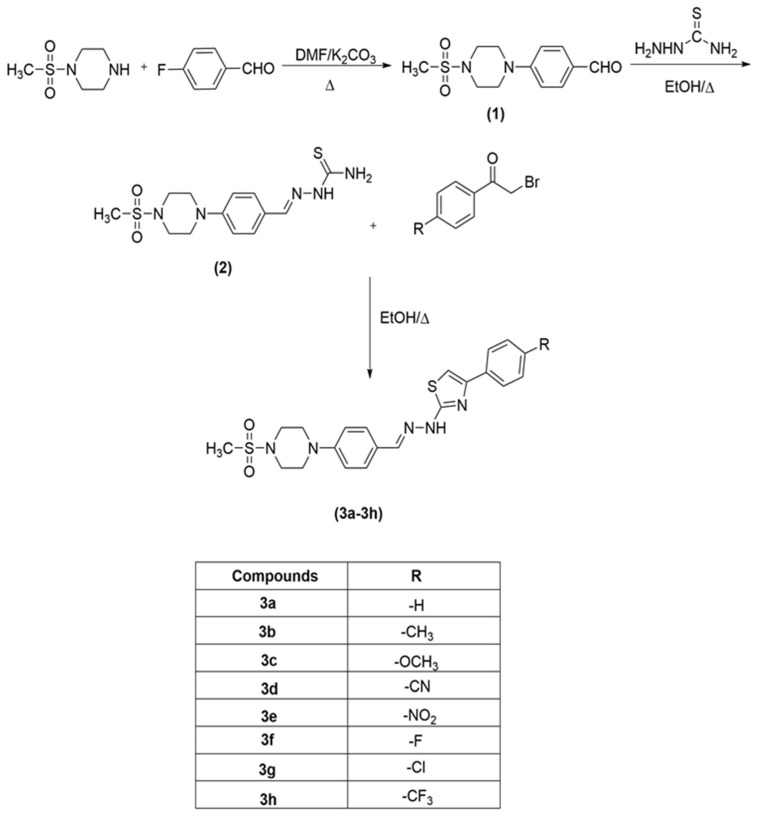
Synthesis pathway of target compounds (**3a**–**3h**).

The compounds **3a**–**3h** were synthesized as outlined in Figure 2. 

Firstly, 4-(4-(methylsulphonyl)piperazin-1-yl) benzaldehyde (**1**) were prepared by reacting 1-(methylsulphonyl) piperazine with 4-fluorobenzaldehyde. Secondly, 4-(4-(methylsulphonyl)piperazin-1-yl) benzaldehyde (**1**) was changed to its corresponding thiosemicarbazone by reacting compound **1** with hydrazinecarbothioamide. Finally, target compounds (**3a**–**3h**) were generated via ring closure reaction. The final compounds (**3a**–**3h**) were purified, and their structures were determined using ^1^H-NMR, ^13^C-NMR, and LCMSMS (see Supplementary Materials Figures S1–S32). 

### 2.2. Prediction of ADME Parameters

Strong pharmacological activity and low toxicity profile of molecules are not sufficient to make it a certain drug candidate—candidate drugs need to possess appropriate pharmacokinetics [32]. Therefore, we calculated various physicochemical parameters of the synthesized compounds (**3a**–**3h**) using the Molinspiration program to estimate their absorption, distribution, metabolism, and excretion (ADME) profiles. 

The theoretical calculations of ADME parameters (topological polar surface area (TPSA), molecular volume (MV), number of hydrogen acceptors (AHB), number of hydrogen donors (DHB), partition coefficient (log P), and molecular weight (MW)) are presented in Table 1.

The Molinspiration program is based on the principle of Lipinski’s five rules that determine the properties a candidate drug molecule must have to be active in humans—an orally administrated drug should not violate more than one rule. The data obtained for compounds **3a**–**3h** did not violate any Lipinski rule, indicated good pharmacokinetic profiles, and increased their therapeutic potentials. On the other hand, it should also be noted that the Lipinski rule is not sufficient on its own and further pharmacokinetic studies are needed to draw solid conclusions. 

### 2.3. Pharmacology

**Figure 3:**
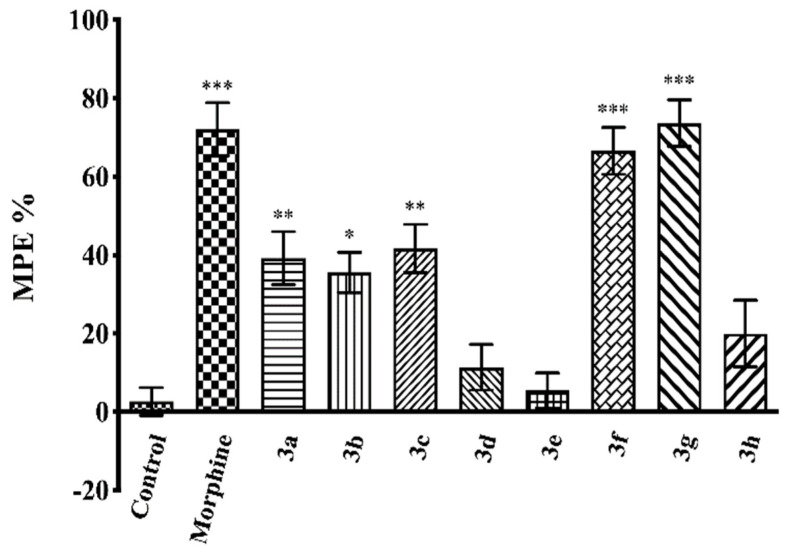
Effects of test compounds (**3a**–**3h**, 50 mg/kg, p.o., administered 60 min before testing) and morphine (10 mg/kg, i.p., administered 30 min before testing), on MPE % values in the mice tail-clip test. Significance against control group * *p* < 0.05, ** *p* < 0.01, *** *p* < 0.001. Values are mean ± SEM. One-way ANOVA and post-hoc Tukey’s test, *n* = 7.

**Figure 4:**
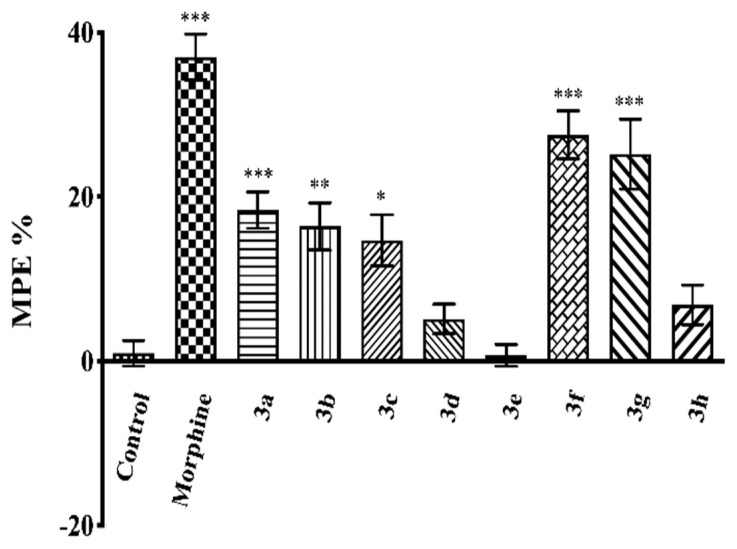
Effects of test compounds (**3a**–**3h**, 50 mg/kg, p.o., administered 60 min before testing) and morphine (10 mg/kg, i.p., administered 30 min before testing) on MPE % values in the mice hot-plate test. Significance against control group * *p* < 0.05, ** *p* < 0.01, *** *p* < 0.001. Values are mean ± SEM. One-way ANOVA and post-hoc Tukey’s test, *n* = 7.

Figure 3 and Figure 4 show the effects of compounds **3a**–**3h** (50 mg/kg, p.o.) and morphine (10 mg/kg, i.p.) as MPE % values in the tail-clip (F(9,60) = 20.01, *p* < 0.001) and hot-plate tests (F(9,60) = 20.86, *p* < 0.001) respectively, in mice. 

Results of the multiple comparison tests indicated that administration of compounds **3a**–**3c**, **3f**, and **3g** (50 mg/kg, p.o.) significantly increased the calculated MPE % values compared to the corresponding control group in both tests. Compounds **3d**, **3e**, and **3h** were ineffective. 

**Figure 5:**
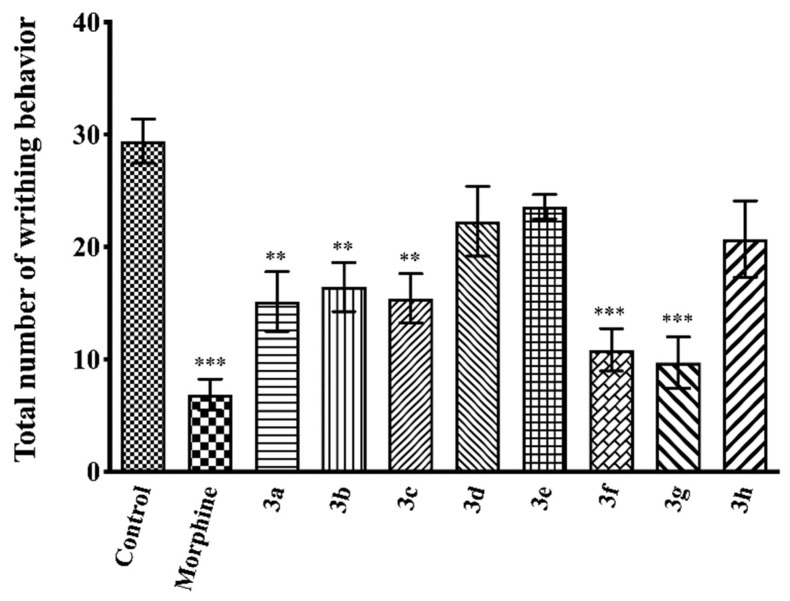
Effects of test compounds **3a**–**3h** (50 mg/kg, p.o., administered 60 min before testing) and morphine (10 mg/kg, i.p., administered 30 min before testing) on the number of writhing behaviors of mice in the acetic acid-induced writhing test. Significance against control group ** *p* < 0.01, *** *p* < 0.001. Values are mean ± SEM. One-way ANOVA and post-hoc Tukey’s test, *n* = 7.

Figure 5 illustrates the effects of test compounds **3a**–**3h** and morphine on the number of writhing behaviors scored in the acetic acid-induced writhing test (F(9,60) = 9.13, *p* < 0.001). Results of the multiple comparison tests indicated that compounds **3a**–**3c**, **3f**, and **3g** significantly decreased the acetic acid-induced writhing responses compared to the control group. The percentage inhibitions of writhing behaviors in the acetic acid writhing test are presented in Table 2. 

Morphine (10 mg/kg, i.p.), used as a reference drug, presented its antinociceptive activity in all tests, as expected (Figure 3, Figure 4 and Figure 5).

**Figure 6:**
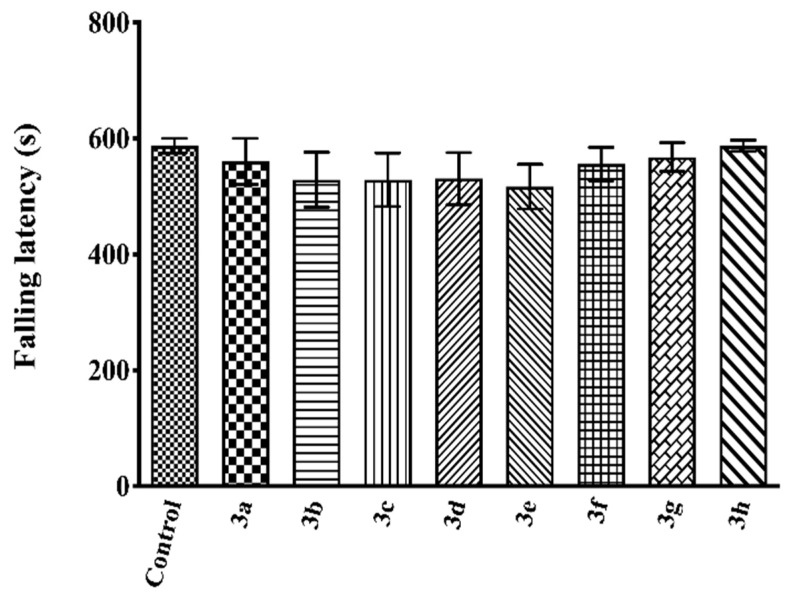
Effects of test compounds **3a**–**3h** (50 mg/kg) on falling latencies of mice in the Rota-Rod test. Values are mean ± SEM. One-way ANOVA and post-hoc Tukey’s test, *n* = 7.

The effects of the test compounds on falling latencies recorded in the Rota-Rod tests are shown in Figure 6. The data show that test compounds did not cause any significant alteration in the mice’s motor coordination (F(8,54) = 0.57, *p* > 0.05).

**Figure 7:**
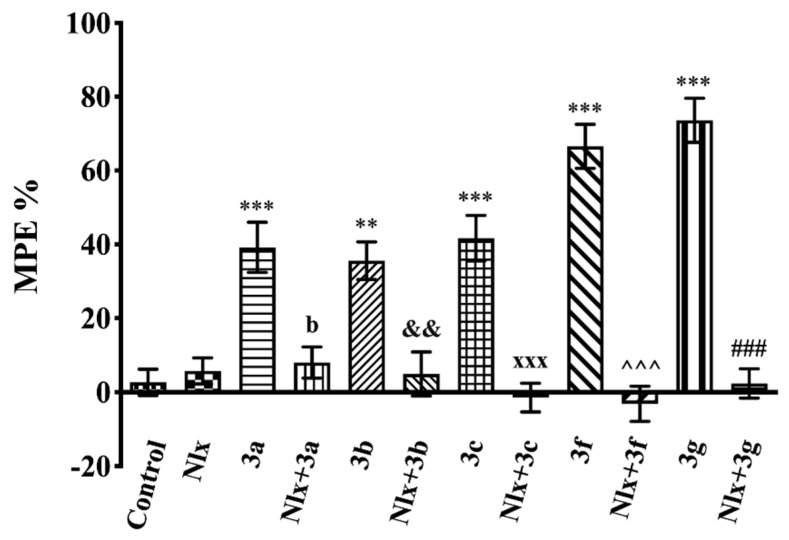
Effect of naloxone (5.48 mg/kg, i.p.) pre-treatment on antinociceptive activity induced by compounds **3a**–**3c**, **3f**, and **3g** in the tail-clip test. Significance against control group ** *p* < 0.01, *** *p* < 0.001; significance against compound groups: **3a** ^b^ *p* < 0.01; **3b** ^&&^ *p* < 0.01; **3c** ^xxx^ *p* < 0.001; **3f** ^^^^^ *p* < 0.001; **3g** ^###^ *p* < 0.001. Values are mean ± SEM. One-way ANOVA and post-hoc Tukey’s test, *n* = 7.

**Figure 8:**
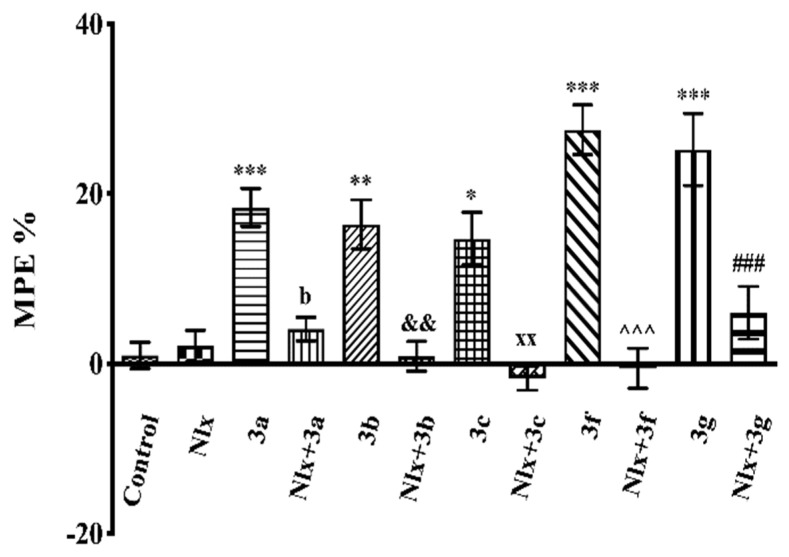
Effect of naloxone (5.48 mg/kg, i.p.) pre-treatment on antinociceptive activity induced by compounds **3a**–**3c**, **3f**, and **3g** in the hot-plate test. Significance against control group * *p* < 0.05, ** *p* < 0.01, *** *p* < 0.001; significance against compound groups: **3a** ^b^ *p* < 0.01; **3b** ^&&^ *p* < 0.01; **3c** ^xx^ *p* < 0.01; **3f** ^^^^^ *p* < 0.001; **3g** ^###^ *p* < 0.001. Values are mean ± SEM. One-way ANOVA and post-hoc Tukey’s test, *n* = 7.

The effects of naloxone pre-treatment on the antinociceptive effects of test compounds in the tail-clip (F(11,72) = 28.53, *p* < 0.001) and hot-plate (F(11,72) = 16.87, *p* < 0.001) tests are shown in Figure 7 and Figure 8, respectively. In both tests, naloxone pre-treatments significantly reversed the increase in MPE % values induced by compounds **3a**–**3c**, **3f**, and **3g**. 

**Figure 9:**
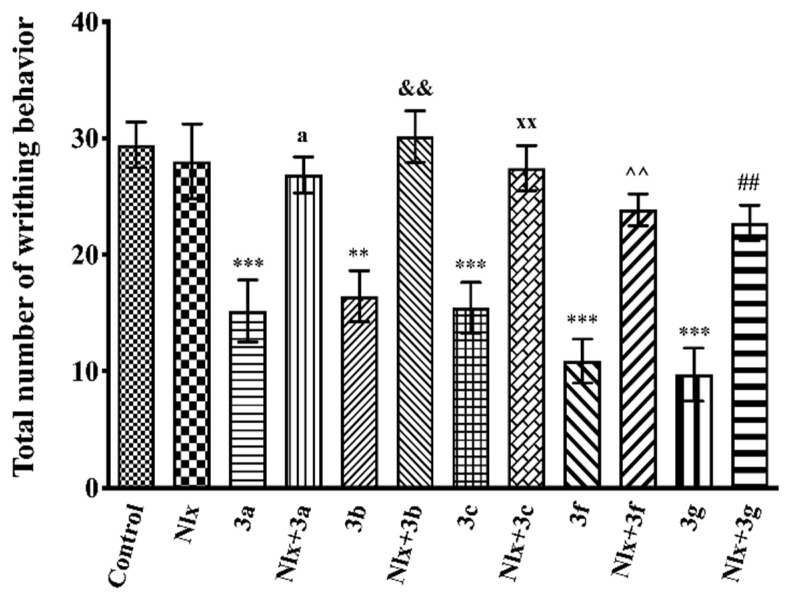
Effect of naloxone (5.48 mg/kg, i.p.) pre-treatment on antinociceptive activity induced by compounds **3a**–**3c**, **3f**, and **3g** in the acetic acid-induced writhing test. Significance against control group ** *p* < 0.01, *** *p* < 0.001; significance against compound groups: **3a** ^a^ *p* < 0.05; **3b** ^&&^ *p* < 0.01; **3c** ^xx^ *p* < 0.01; **3f** ^^^^ *p* < 0.01; **3g** ^##^ *p* < 0.01. Values are mean ± SEM. One-way ANOVA and post-hoc Tukey’s test, *n* = 7.

The effects of naloxone pre-treatment on the antinociceptive effects of the test compounds in the acetic acid-induced writhing test are shown in Figure 9. Naloxone pre-treatments significantly antagonized the decrease in the number of writhing movements and reversed the antinociceptive effect of compounds **3a**–**3c**, **3f**, and **3g** (F(11,72) = 12.03, *p* < 0.001).

### 2.4. Molecular Docking Studies

Molecular docking studies were performed to clarify binding profiles of the tested derivatives to active sites of opioid receptors. For this purpose, the crystal structures of µ-opioid receptor (PDB ID: 5C1M) [33], δ-opioid receptor (PDB ID: 4N6H) [34], and ĸ-opioid receptor (PDB ID: 6B73) [35] were retrieved from the Protein Data Bank server (www.pdb.org, accessed date 28 April 2021). The two-dimensional and three-dimensional docking poses of all compounds against all receptors are presented in Figure 10, Figure 11, Figure 12, Figure 13, Figure 14, Figure 15, Figure 16, Figure 17, Figure 18, Figure 19, Figure 20, Figure 21, Figure 22, Figure 23 and Figure 24, and Supplementary Materials Figures S33–S68.

**Figure 10:**
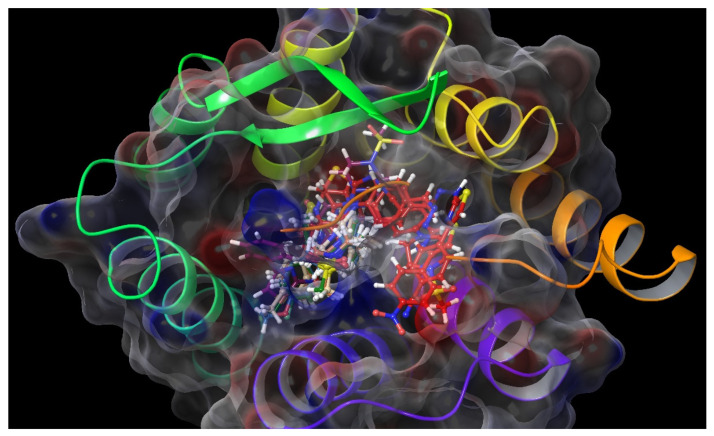
The three-dimensional pose of the interaction of all compounds with the µ-opioid receptor (PDB Code: 5C1M) active site. The inactive compounds (**3d**, **3e**, and **3h**) are presented by a tube model colored with red.

**Figure 11:**
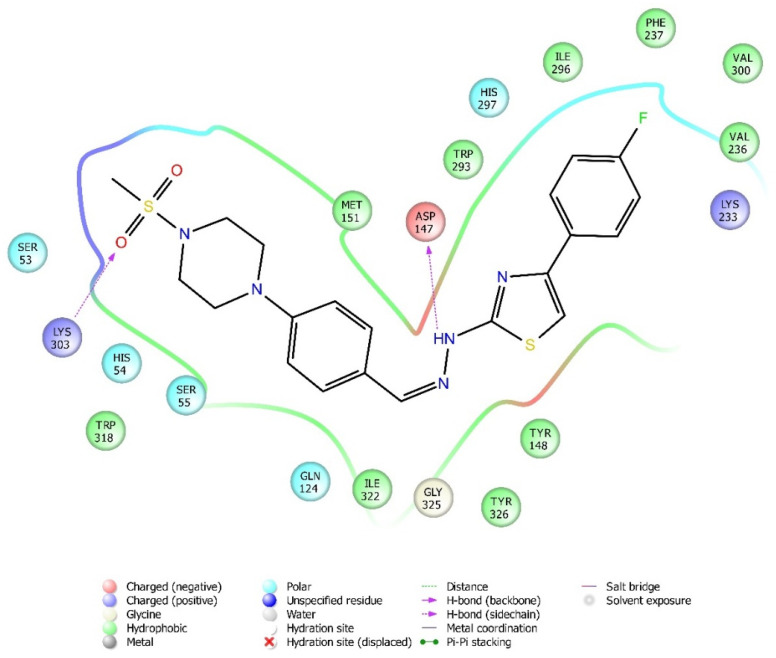
The two-dimensional interacting mode of compound **3f** in the active region of µ-opioid receptor (PDB Code: 5C1M).

**Figure 12:**
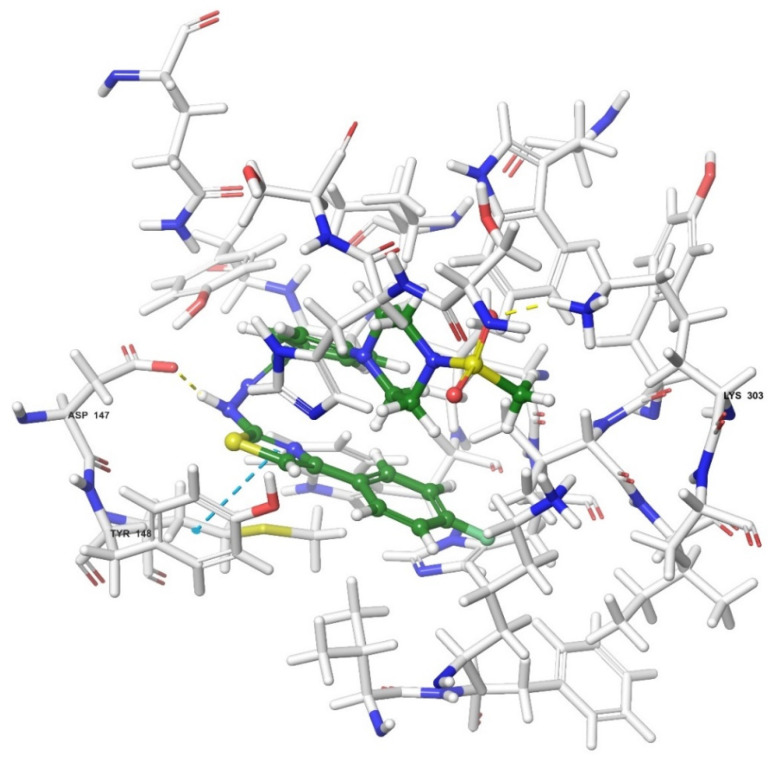
The three-dimensional interacting mode of compound **3f** in the active region of µ-opioid receptor. The ligand and significant residues of the active site of the receptor are presented by a tube model colored with green and white, respectively (PDB Code: 5C1M).

**Figure 13:**
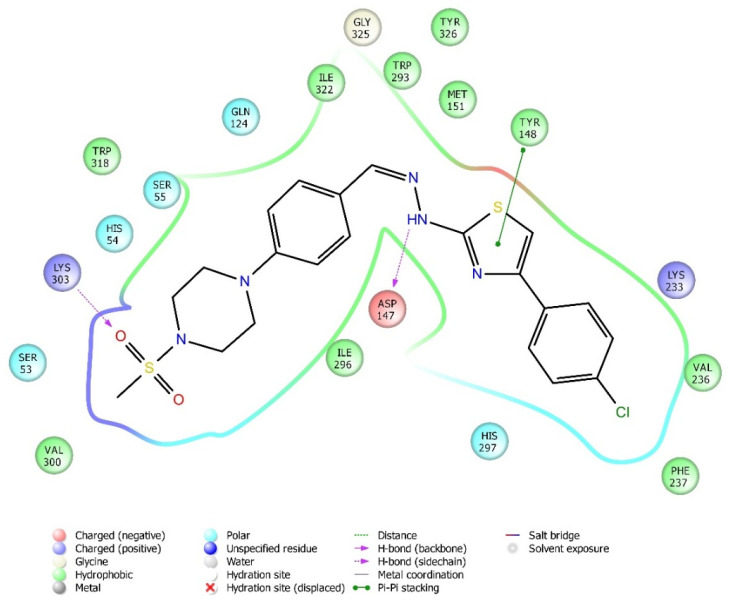
The two-dimensional interacting mode of compound **3g** in the active region of µ-opioid receptor (PDB Code: 5C1M).

**Figure 14:**
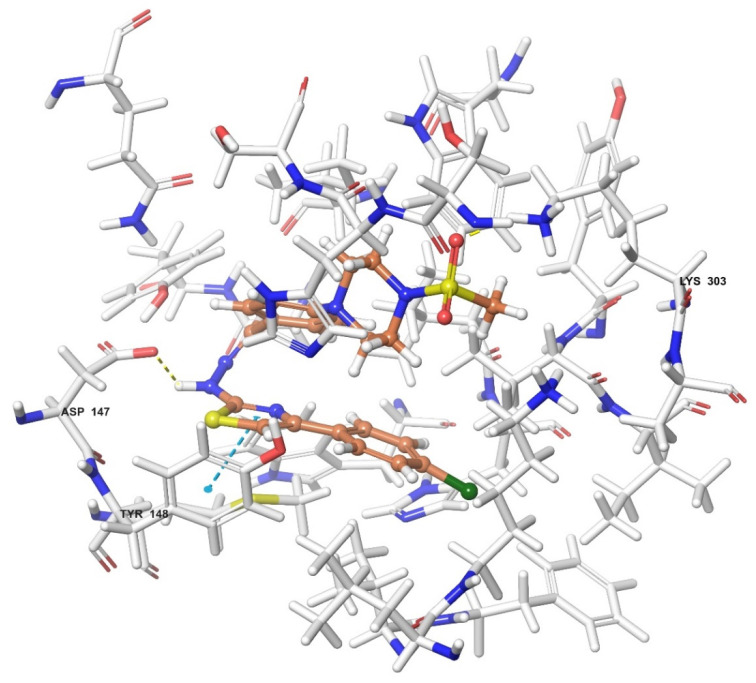
The three-dimensional interacting mode of compound **3g** in the active region of µ-opioid receptor. The ligand and significant residues of the active site of the receptor are presented by a tube model colored with orange and white, respectively (PDB Code: 5C1M).

According to the crystallographic X-ray structure of the µ-opioid receptor (PDB ID:5C1M), the rendered docking poses of all compounds are shown in Figure 10, Figure 11, Figure 12, Figure 13 and Figure 14 and in Supplementary Materials Figures S33–S44. When the compounds were analyzed all together, it is seen that compounds **3a**–**3c**, **3f**, and **3g** fit well to the active site of the receptor, whereas **3d**, **3e**, and **3h** do not place properly (compounds colored in red) (Figure 10). The presented 2D and 3D docking poses of the test compounds exhibited that pharmacologically ineffective derivatives cannot achieve to build necessary interactions with the receptor protein (See Supplementary Materials Figures S33–S44). 

When the interactions are analyzed in terms of binding to the µ-opioid receptor, π-π interactions are observed between the thiazole rings of compounds **3b**, **3c**, and **3g** and the phenyl group of Tyr148. Additionally, the phenyl ring of compounds **3a** and **3h** formed a π-π interaction with imidazole group of His54. Hydrogen bonds were the other bonds constructing between the ligands and µ-opioid receptors. The sulfonyl moieties of compounds **3b**, **3c**, **3f**, and **3g** formed single hydrogen bonds with the amine groups of Lys303. Another hydrogen bond is observed between the hydrazine groups of compounds **3c**, **3f**, and **3g** and hydroxy group of Asp147. These results are in accordance with the previous literature [36]. Moreover, compound **3d** did not interact with this opioid receptor subtype, while compound **3e** is observed to form only a salt bridge with Lys303 amino acid of µ-opioid receptor protein. 

**Figure 15:**
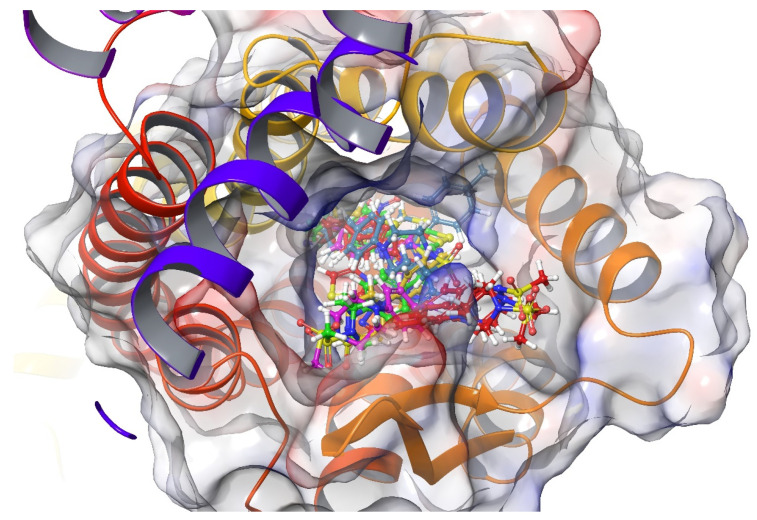
The three-dimensional pose of the interaction of all compounds with the δ-opioid receptor (PDB Code:4N6H) active site. The inactive compounds (**3d**, **3e**, and **3h**) are presented by a tube model colored with red.

**Figure 16:**
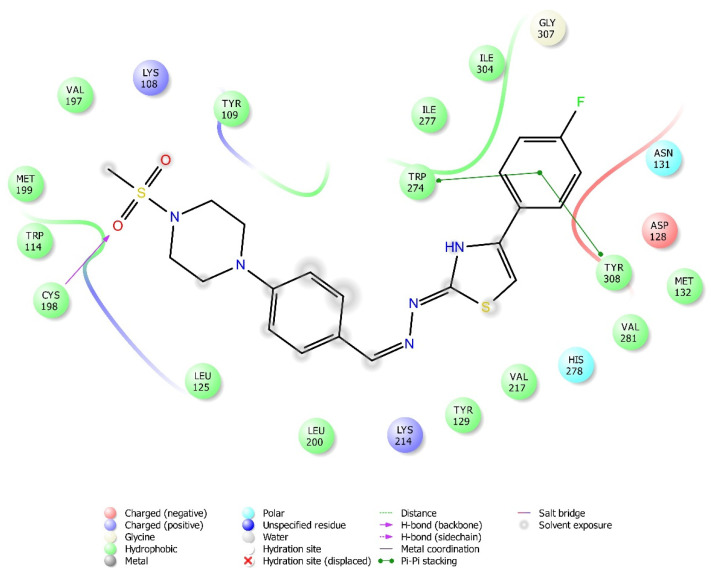
The two-dimensional interacting mode of compound **3f** in the active region of δ-opioid receptor (PDB Code: 4N6H).

**Figure 17:**
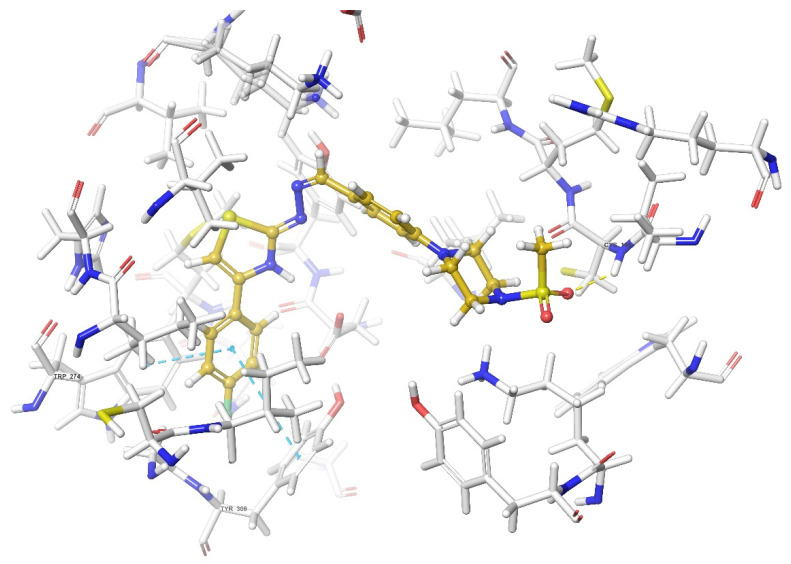
The three-dimensional interacting mode of compound **3f** in the active region of δ-opioid receptor. The ligand and significant residues of the active site of the receptor are presented by a tube model colored with yellow and white, respectively (PDB Code: 4N6H).

**Figure 18:**
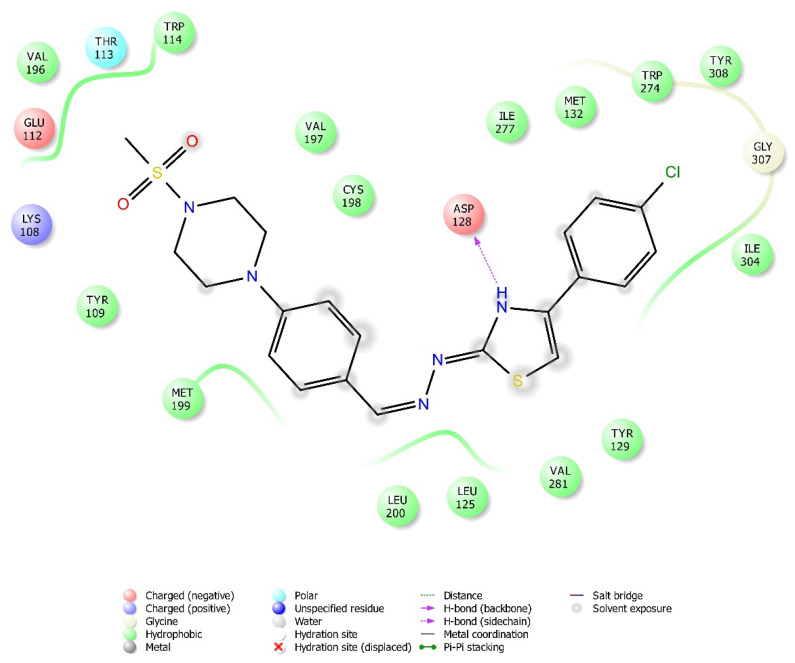
The two-dimensional interacting mode of compound **3g** in the active region of δ-opioid receptor (PDB Code: 4N6H).

**Figure 19:**
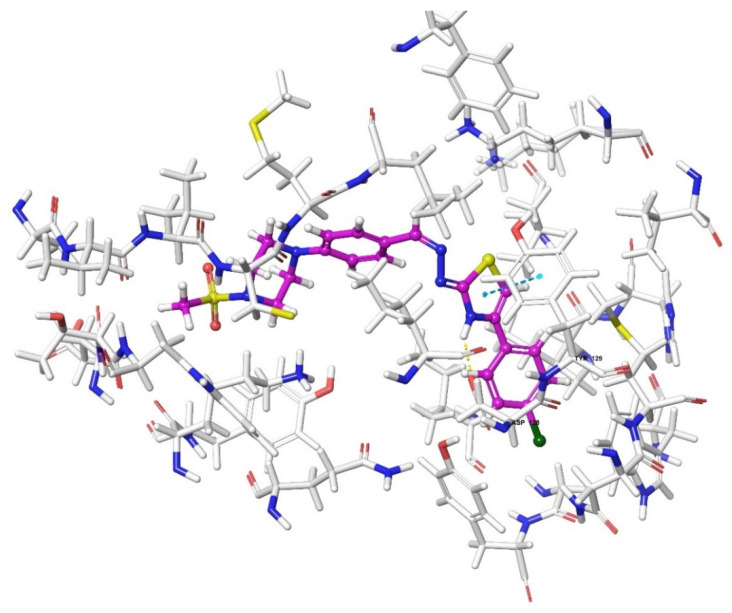
The three-dimensional interacting mode of compound **3g** in the active region of δ-opioid receptor. The ligand and significant residues of the active site of the receptor are presented by a tube model colored with purple and white, respectively (PDB Code: 4N6H).

According to the crystallographic X-ray structure of the δ-opioid receptor (PDB ID: 4N6H), the rendered docking poses of all compounds are provided in Figure 15, Figure 16, Figure 17, Figure 18 and Figure 19 and in Supplementary Materials Figures S45–S56. Obtained findings indicated that compounds **3a**–**3c**, **3f**, and **3g** fit well to the active site of the receptor, whereas **3d**, **3e**, and **3h** do not (compounds colored in red) (Figure 15). The presented 2D and 3D docking poses of the test compounds exhibited that pharmacologically ineffective derivatives are not able to form required interactions with the receptor protein (See Supplementary Materials Figures S45–S56). 

Docking findings obtained from δ-opioid receptor studies revealed that there are two π-π interactions between the phenyl on thiazole ring of compound **3a** and the phenyl groups of Tyr308 and Trp274. Similar bonds are also observed for compound **3f**. Another π-π interaction is observed between the thiazole ring of compound **3b** and phenyl group of Tyr129. It is also seen that the active molecules form notable hydrogen bonds with the receptor. The sulfonyl moieties of compounds **3a** and **3f** formed hydrogen bonds with Cys198. Moreover, sulfonyl moiety and hydrazine nitrogen of compound **3b** displayed hydrogen bond interactions with Lys214 and Asp128, respectively. Thiazole ring of compound **3g** also formed a hydrogen bond with Asp128. Besides, compound **3e** is observed to form a π-cation interaction with His278. Although compound **3c** fitted to the active site of the δ-receptor, no interaction was observed. 

**Figure 20:**
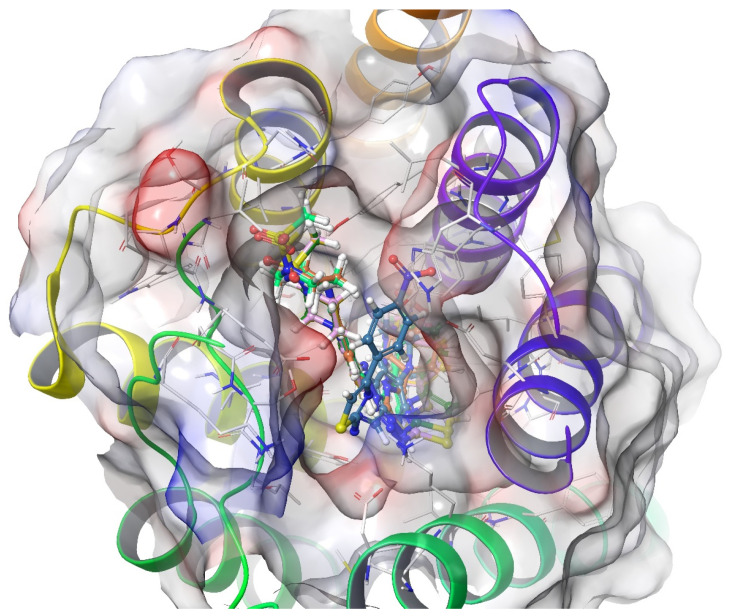
The three-dimensional pose of the interaction of all compounds with the ĸ-opioid receptor (PDB Code:6B73) active site. The compound **3e** is presented by a tube model colored with blue.

**Figure 21:**
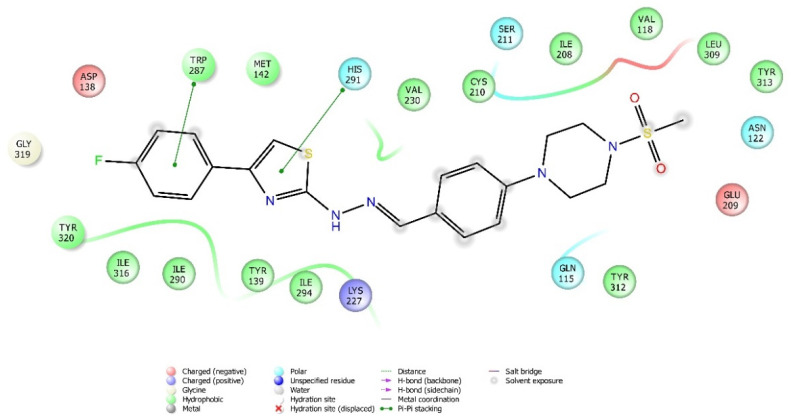
The two-dimensional interacting mode of compound **3f** in the active region of ĸ-opioid receptor (PDB Code: 6B73).

**Figure 22:**
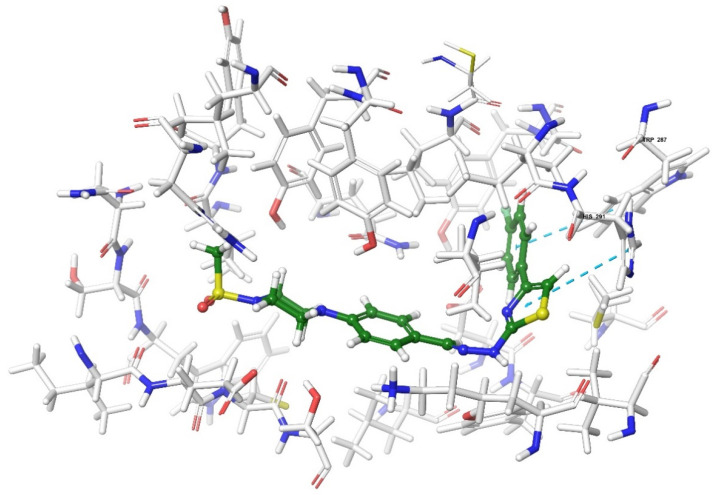
The three-dimensional interacting mode of compound **3f** in the active region of ĸ-opioid receptor. The ligand and significant residues of the active site of the receptor are presented by a tube model colored with green and white, respectively (PDB Code: 6B73).

**Figure 23:**
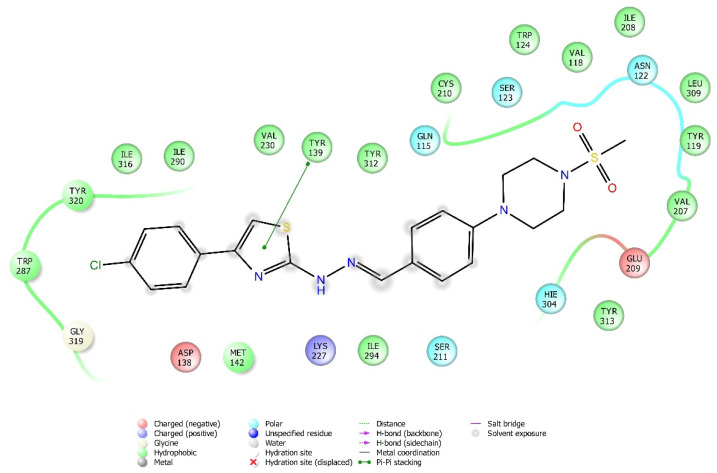
The two-dimensional interacting mode of compound **3g** in the active region of ĸ-opioid receptor (PDB Code: 6B73).

**Figure 24:**
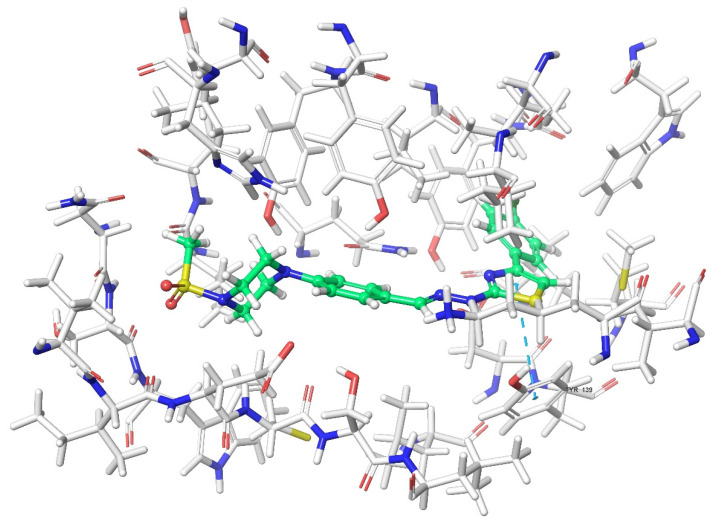
The three-dimensional interacting mode of compound **3g** in the active region of ĸ-opioid receptor. The ligand and significant residues of the active site of the receptor are presented by a tube model colored with green and white, respectively (PDB Code: 6B73).

According to the crystallographic X-ray structure of the ĸ-opioid receptor (PDB ID: 6B73), the rendered docking poses of obtained compounds are provided in Figure 20, Figure 21, Figure 22, Figure 23 and Figure 24 and in Supplementary Materials Figures S57–S68. As seen in the 3D pose, all compounds are located in the active site (Figure 20). It is observed that π-π interactions occur between the thiazole rings of compounds **3a** and **3g**, and phenyl group of Tyr139. Another π-π interaction is seen between the phenyl rings of compounds **3a**, **3b**, and **3f** and indole group of Trp287. Moreover, thiazole rings of compounds **3d**, **3f**, and **3h** are observed to form π-π interactions with imidazole group of His291. Settlement of compound **3e** on this receptor protein was quite different from those of other compounds. This compound formed a π-π interaction with Try139, a salt-bridge with Lys227, Glu297, and a π-cation interaction with Lys227 and Tyr312. On the other hand, compound **3c** did not interact with the ĸ receptors.

Results of the docking studies are summarized in Table 3.

## 3. Discussion

Eight novel compounds (**3a**–**3h**) carrying thiazole-piperazine ring systems were synthesized and investigated for their possible acute antinociceptive activities, in this study.

In antinociceptive activity screening studies, methods that evaluate the nociceptive behaviors of animals induced by nociceptive mechanical, thermal, or chemical stimuli are used. Stimuli applied to create pain perception in experimental animals should be measurable, reproducible, and non-invasive [37]. 

In this study, antinociceptive activities after oral administration of the compounds against mechanical nociceptive stimulus were investigated by the tail-clip tests. The administration of compounds **3a**–**3c**, **3f**, and **3g**, at doses of 50 mg/kg, significantly increased the MPE % values of mice compared to the control group (Figure 3), indicating that these compounds possess antinociceptive activity by affecting the neuronal pathways carrying mechanical stimuli. Moreover, based on the knowledge that this test is predominantly related to nociceptive transmission at the spinal level [38,39], it can be assumed that spinal mechanisms play a role in the antinociceptive effects of these compounds.

The antinociceptive activities after oral administration of the test compounds against thermal nociceptive stimulus were examined by the hot-plate test. Results showed that administrations of the compounds **3a**–**3c**, **3f**, and **3g** significantly enhanced the MPE % values compared to the control group (Figure 4), indicating that these compounds affect the nociceptive pathways carrying the thermal as well as the mechanical stimuli. Based on the well-documented association of this test with supraspinal nociceptive transmission [38,39,40,41], it can be assumed that supraspinal mechanisms play a role in the antinociceptive effects of these compounds, together with the spinal mechanisms. When MPE % values of the compounds are examined in both tests, it is seen that compounds **3f** and **3g** (50 mg/kg, p.o.) are as effective as the reference drug morphine (10 mg/kg, i.p.) in tail-clip tests, unlike the effects in hot-plate tests. These findings may be due to the fact that the antinociceptive effects of the compounds **3f** and **3g** on the spinal pathways are stronger than those in the supraspinal pathways, or because these two compounds affect the mechanical nociceptive pathways more strongly than the thermal ones.

Peripheral antinociceptive effects of test compounds were investigated by an acid-induced writhing test that models visceral pain [42]. In this test, acetic acid administrated by i.p. route acts in direct (by activating the nociceptors) or indirect (by triggering the release of autacoid mediators) ways [43] to stimulate the peripheral receptors on the surface of the peritoneal cavity [44]. This stimulation causes a writhing behavior in animals characterized by contraction of the abdominal muscles and backward stretching of the hind legs [41,45]. Oral administration of compounds **3a**–**3c**, **3f**, and **3g** significantly inhibited the writhing of animals (Figure 5 and Table 2), indicating that these compounds exhibit peripheral antinociceptive effects on the neuronal pathways that transfer chemical painful stimuli. The peripheral antinociceptive activities of these compounds may be related to the reduced release of inflammatory mediators and/or direct blockage of their receptors. Possible increase in the nociceptive thresholds or interruption in the transmission of pain stimuli in the nerve fiber may also be other mechanisms underlying the observed antinociception [40,41,46].

It is known that possible effects of the test compounds on the motor performances of animals may cause false-positive results in nociceptive tests [40,41]. Therefore, Rota-Rod tests were conducted to evaluate the motor coordination of mice. Data show that none of the test compounds caused significant alterations in the motor activities of mice (Figure 6), indicating that the antinociceptive effects exhibited in this study are specific.

After demonstrating the antinociceptive efficacies of compounds **3a**–**3c**, **3f**, and **3g**, the possible involvement of opioidergic mechanisms in the presented pharmacological activity was investigated by naloxone studies. Naloxone pre-treatment reversed the antinociceptive activities of these compounds in all of the nociceptive tests (Figure 7, Figure 8 and Figure 9), indicating that opioidergic mechanisms participate in the antinociceptive activity of these compounds. Then, we performed docking studies in order to clarify the interactions of our molecules with opioid receptors.

Results of the docking studies on the µ-opioid receptor indicated that compounds **3a**–**3c**, **3f**, and **3g** formed π-π interactions and/or hydrogen bonds with His54, Asp147, Tyr148, and Lys303 amino acids of the receptor protein, probably conferring antinociceptive activity to these derivatives. Actually, these findings are in accordance with previous knowledge obtained from the fragment molecular orbital (FMO) method, which revealed that His54 (N-terminus), Asp147 (TM3), and Lys303 (TM6) are the most significant residues contributing to the µ-opioid receptor-mediated analgesic efficacy of opioids [36]. It was determined that π-π interactions between the pharmacologically active compounds **3b**, **3c**, and **3g** and µ-opioid receptors occurred through the Tyr148 amino acid of the receptor protein. Moreover, His54 was the only amino acid involved in the π-π interactions between the µ-receptor protein and compound **3a**. Therefore, it can be speculated that interactions with Tyr148 and His54 amino acids may be supportive of the antinociceptive activities of these molecules. Nevertheless, compound **3h** is the exception. The fact that compound **3h** does not show an antinociceptive effect although it shows π-π interactions with His54 may be related to the inability of this molecule to enter the active pocket due to the CF3 group it carries. Although docking studies have revealed various π-π interactions between the active compounds and the µ-receptor protein, hydrogen bonds seem to be major contributors of the antinociceptive effect. It is observed that compounds **3f**, **3g**, and **3c**, whose phenyl ring is substituted with the electron-donating groups (F, Cl, and OCH_3_, respectively), formed hydrogen bonds with Lys303 and Asp147 amino acids of the µ-opioid receptors. Besides, compound **3b**, carrying another electron-donating group (CH_3_) on its phenyl ring, also formed hydrogen bonds with Lys303 amino acids. On the other hand, no hydrogen bonds have formed between these receptor subtypes and compounds **3d**, **3e**, and **3h**, which have phenyl rings, substituted with electron-withdrawal groups such as CN, NO_2_, and CF_3_. Therefore, it may be speculated that substitution of phenyl ring with electron-donating groups supports the hydrogen bond formation with µ-opioid receptor subtype.

Compounds **3a**, **3b**, **3f**, and **3g** were observed to form π-π interactions and/or hydrogen bonds with Asp128, Tyr129, Cys198, Lys214, Trp274, and Tyr308 amino acids of the δ-opioid receptor protein. These interactions pointed out that δ-opioid receptors, together with µ-receptor subtypes, play roles in the antinociceptive activities of these compounds. Among the pharmacologically active compounds, only **3a** and **3f** were detected to form π-π interactions with both of the Trp274 and Tyr308 amino acids of the δ-opioid receptors. Another π-π interaction was seen between the compound **3b** and Tyr129. Active compounds **3a**, **3b**, **3f**, and **3g** were observed to form hydrogen bonds with Cys198, Asp128, and Lys214 amino acids of δ-opioid receptor protein. Since compounds **3f**, **3g**, and **3b** have phenyl rings substituted by F, Cl, and CH_3_, it can be assumed that substitution of the phenyl ring with electron-donating groups can promote the formation of strong hydrogen bonds between the test compounds and δ-opioid receptors, as in the µ-receptor subtype. Indeed, absence of any hydrogen bonding or π-π interactions between inactive test compounds (**3d**, **3e**, and **3h**) carrying phenyl rings substituted by electron-withdrawal groups and δ-opioid receptors confirms this idea. On the other hand, another active compound **3c** did not interact with the δ-receptor protein, although it fits into the active site of the receptor. Thus, it is possible that compound **3c** induced its antinociceptive activity via µ-opioid receptors that we know to form hydrogen bonds and π-π interactions with this compound, rather than δ-receptors. 

Docking findings obtained from the ĸ-opioid receptor studies were different from the results of µ- and δ-subtypes. Not only active derivatives but also pharmacologically inactive compounds settled down to the ĸ-receptor protein, successfully. It was observed that active compounds **3a**, **3b**, **3f**, and **3g** formed π-π interactions with the Tyr139, Trp287, and His291 amino acids of ĸ receptor protein, while no hydrogen bonds were detected. On the other hand, compounds **3d** and **3h**, having π-π interactions with His291, and compound **3e**, having the same type of interaction with Try139, were inactive in the nociceptive tests. Since these π-π interactions are common for all of the active and inactive compounds, the only significant binding might be the π-π interaction formed with Trp287 amino acid. Nevertheless, it should also be noted that the contributions of the weak π-π interactions to the antinociceptive effects of these compounds are limited. Moreover, it was observed that compound **3c**, an active derivative in the serial, did not show any interaction with the ĸ-receptor protein. All these findings pointed out that there is not a significant settlement/binding and activity relationship between the molecules and ĸ-opioid receptor protein. Therefore, it can be suggested that ĸ-opioid receptors did not mediate the antinociceptive effects of the active compounds presented in this study. 

Data obtained from the docking studies indicated that thiazole rings in the compounds seem responsible to form π-π interactions with all of the µ-, δ-, and ĸ-opioid receptor subtypes. Thiazole ring even constructs a hydrogen bond between compound **3g** and δ-opioid receptor protein. In addition, hydrazine groups of compound **3f** and compound **3b** were shown to build hydrogen bonds with µ- and δ-receptors, respectively. More importantly, methylsulfonyl residues of the active derivatives seem to build strong hydrogen bonds with the active sites of µ- and δ-receptor subtypes, which seem to be critical for the presented antinociceptive effect in this study. The same residues, on the other hand, did not show similar interactions with ĸ-opioid receptors, which seem to be irrelevant to the activity. This difference in binding properties of methylsulfonyl residues may be related to conformational arrangement of the substituents, which alter the settlements on opioid receptors and affect interactions with them. 

Differences in the electronic properties of the compounds can also change their pharmacological activity profiles. For example, electron-donating substituents (CH_3_, OCH_3_, F, and Cl) in the chemical structures of the derivatives seem to support µ- and δ-opioid receptor bindings and antinociceptive activity, more than electron-withdrawing groups such as cyano, nitro, and trifluoromethyl (**3d**, **3e**, and **3h**). Substitutions with CN (**3d**) and NO_2_ (**3e**) groups increased TPSA values, reflecting the increased polarity of the molecules, possibly resulting in reduced transport of molecules across membranes. These compounds were indeed ineffective in activity tests. In addition, substitutions with Cl (**3g**) and CF_3_ (**3h**) increased the lop P values, reflecting the enhanced lipofility of these compounds. Really, **3g** was active in the nociceptive tests. However, **3h** was not. The ineffectiveness of the CF_3_-substituted compound **3h** may possibly be related to its high MW and MV values, hindering the molecule from placing on the µ- and δ-opioid receptors. Furthermore, it was observed that compound **3e**, which has a higher number of hydrogen acceptors than the other molecules (Table 1), did not form any hydrogen bonds to opioid receptors. The probable reason for this is that this molecule is improperly located on the receptor and therefore is not able to form any bond with it.

### Future Directions

In the present study, the opioid system-mediated antinociceptive activities of some novel compounds, bearing both of the thiazole and piperazine ring systems together on their structures, have been evidenced. Although the binding potential of active molecules to opioid receptors has been shown by an in silico method in this study, it will be useful to verify this binding by further methods, such as radioligand binding. Moreover, based on the fact that pain transmission and antinociception are complex processes affected by various endogenous mechanisms [47,48], possible contributions of different mechanisms such as GABAergic, glutamatergic, cannabinoidergic, cholinergic, nitrergic systems, ion channels, or enzymes (such as COX isoenzymes) [49,50], which may be underlying the pharmacological effects of these compounds, need to be clarified with further studies.

Since this study was planned as a synthesis and antinociceptive activity screening study, we contended with the calculation of the ADME parameters, which provides an overview regarding the pharmacokinetic properties of the molecules. On the other hand, dose-response curves can be drawn by using pharmacological responses induced by different doses of each compound, various pharmacodynamic parameters, such as E_max_ and ED_50_, can be calculated, and more detailed pharmacodynamic data for each active molecule can be obtained in the next step of this study.

An important point regarding the potential of new compounds to become analgesic drugs is the side effect profiles of these molecules. Although the tested compounds did not show undesirable side effects such as death, paralysis, ataxia, convulsions, or diarrhea, promising that they do not have a serious toxicity potential, the efficacy and safety of these compounds should be investigated by further detailed studies. In this context, it is of great importance to evaluate active molecules in terms of possible side effects such as respiratory depression, emesis, addiction, and tolerance development, which are typical side effects of opioid drugs [51]. 

## 4. Materials and Methods

### 4.1. Chemicals

All reagents were purchased from commercial suppliers and were used without further purification. Morphine sulphate and naloxone hydrochloride were acquired from Sigma-Aldrich (St. Louis, MO, USA).

### 4.2. Chemistry

Melting points (m.p.) were determined on the Mettler Toledo-MP90 Melting Point System and were uncorrected. IR spectra were recorded on an IR Affinity-1S Infrared spectrophotometer (Shimadzu, Tokyo, Japan). ^1^H NMR and ^13^C NMR spectra in DMSO-*d*_6_ were recorded on a Bruker Fourier 300 (Bruker Bioscience, Billerica, MA, USA), respectively. MS studies were performed on an LCMS-8040 tandem mass system (Shimadzu, Tokyo, Japan). Chemical purities of the compounds were checked by classical TLC applications performed on silica gel 60 F254 (Merck KGaA, Darmstadt, Germany). The Rf values of the synthesized compounds were measured using the solution system of petroleum ether:ethyl acetate (1:1).

#### 4.2.1. Synthesis of 4-(4-(methylsulphonyl)piperazin-1-yl)benzaldehyde (1)

1-(Methylsulphonyl)piperazine (2.8 g, 0.017 mol), 4-fluorobenzaldehyde (1.82 mL, 0.017 mol), and potassium carbonate (2.35 g, 0.017 mol) were refluxed in DMF (10 mL). The complete reaction content was poured into ice-water, and the precipitated product was washed with water, filtered, dried, and recrystallized from EtOH.

#### 4.2.2. Synthesis of 2-(4-(4-(methylsulphonyl)piperazin-1-yl)benzylidene)hydrazine-1-carbothioamide (2)

A mixture of 4-(4-(methylsulphonyl)piperazin-1-yl)benzaldehyde (1) (4 g, 0.014 mol) and hydrazinecarbothioamide (1.36 g, 0.014 mol) was refluxed in ethanol (50 mL) for 10 h. The reaction mixture was cooled, and the precipitated product was filtered, washed with cooled ethanol, and dried.

#### 4.2.3. General Procedure for the Synthesis of Target Compounds (**3a**–**3h**)

2-(4-(4-(methylsulphonyl)piperazin-1-yl)benzylidene) hydrazine-1-carbothioamide (2) (0.48 g, 0.0014 mol) and the appropriate 2-bromo-1-(4-substituted)ethan-1-one (0.0014 mol) derivatives were stirred for 5 h in EtOH at 150 °C. The reaction mixture was cooled, and the precipitated product was filtered, and washed with cooled EtOH. 

##### 1-Methylsulphonyl-4-(4-{[2-(4-phenyl-1,3-thiazol-2-yl)hydrazinylidene]methyl}phenyl)piperazine (**3a**)

Yield: 85%, Rf = 0.57, M.P. = 210–212 °C, FTIR (ATR, cm^−1^): 3294 (N-H), 2843 (C-H), 771, 711. ^1^H-NMR (300 MHz, DMSO-*d*_6_): δ = 2.93 (3H, s, -CH_3_), 3.23–3.26 (4H, m, piperazine), 3.33–3.37 (4H, m, piperazine), 6.98 (2H, d, *J* = 8.9 Hz, Monosubstituephenyl), 7.03 (2H, d, *J* = 8.9 Hz, 1,4-Disubstituephenyl), 7.27–7.32 (2H, m, Monosubstituephenyl + Thiazole), 7.41 (2H, t, *J* = 7.5 Hz, Monosubstituephenyl), 7.53 (2H, d, *J* = 8.9 Hz, 1,4-Disubstituephenyl), 7.85 (2H, d, *J* = 7.2 Hz, Monosubstituephenyl), 7.95 (1H, s, -CH=N-), 11.97 (1H, s, -NH). ^13^C-NMR (75 MHz, DMSO-*d*_6_): δ = 34.32, 45.59, 47.76, 103.67, 115.93, 125.65, 125.96, 127.90, 129.05, 131.91, 135.20, 142.06, 150.92, 151.46, 168.79. ESI-MS (*m*/*z*): [M + H]^+^: 442.09.

##### 1-Methylsulphonyl-4-[{2-[4-(4-methylphenyl)-1,3-thiazol-2-yl]hydrazinylidene}methyl]piperazine (**3b**)

Yield: 82%, Rf = 0.68, M.P. = 226–230 °C, FTIR (ATR, cm^−1^): 3294 (-NH), 2856 (C-H), 817. ^1^H-NMR (300 MHz, DMSO-*d*_6_): δ = 2.32 (3H, s, -CH_3_), 2.93 (3H, s, -CH_3_), 3.23–3.26 (4H, m, piperazine), 3.33–3.36 (4H, m, piperazine), 7.03 (2H, d, *J* = 8.9 Hz, 1,4-Disubstituephenyl), 7.19–7.22 (3H, m, 4-Methylphenyl + Thiazole), 7.53 (2H, d, *J* = 8.9 Hz, 1,4-Disubstituephenyl), 7.74 (2H, d, *J* = 8.1 Hz, 4-Methylphenyl), 7.94 (1H, s, -CH=N-), 11.93 (1H, s, -NH). ^13^C-NMR (75 MHz, DMSO-*d*_6_): δ = 21.27, 34.32, 45.59, 47.77, 102.74, 115.93, 125.67, 125.91, 127.89, 128.75, 129.62, 132.56, 137.19, 141.98, 151.45, 168.69. ESI-MS (*m*/*z*): [M + H]^+^: 456.11.

##### 1-Methylsulphonyl-4-[{2-[4-(4-methoxyphenyl)-1,3-thiazol-2-yl]hydrazinylidene}methyl]piperazine (**3c**)

Yield: 87%, Rf = 0.77, M.P. = 175–178 °C, FTIR (ATR, cm^−1^): 3294 (N-H), 2845 (C-H), 819. ^1^H-NMR (300 MHz, DMSO-*d*_6_): δ = 2.93 (3H, s, -CH_3_), 3.23–3.25 (4H, m, piperazine), 3.33–3.36 (4H, m, piperazine), 3.78 (3H, s, -OCH_3_), 6.96 (2H, d, *J* = 8.9 Hz, 4-Methoxyphenyl), 7.02 (2H, d, *J* = 8.9 Hz, 1,4-Disubstituephenyl), 7.10 (1H, s, Thiazole), 7.52 (2H, d, *J* = 8.9 Hz, 1,4-Disubstituephenyl), 7.78 (2H, d, *J* = 8.9 Hz, 4-Methoxyphenyl), 7.93 (1H, s, -CH=N-), 11.93 (1H, s, -NH). ^13^C-NMR (75 MHz, DMSO-*d*_6_): δ = 34.31, 45.59, 47.77, 55.55, 101.51, 114.03, 114.40, 115.93, 125.69, 127.28, 127.88, 128.07, 141.96, 151.44, 159.20, 168.68. ESI-MS (*m*/*z*): [M + H]^+^: 472.10.

##### 1-Methylsulphonyl-4-[{2-[4-(4-cyanophenyl)-1,3-thiazol-2-yl]hydrazinylidene}methyl]piperazine (**3d**)

Yield: 89%, Rf = 0.34, M.P. = 237–239 °C, FTIR (ATR, cm^−1^): 3292 (N-H), 2845 (C-H), 2222 (C≡N), 817. ^1^H-NMR (300 MHz, DMSO-*d*_6_): δ = 2.92 (3H, s, -CH_3_), 3.23–3.26 (4H, m, piperazine), 3.33–3.37 (4H, m, piperazine), 7.02 (2H, d, *J* = 8.9 Hz, 1,4-Disubstituephenyl), 7.53 (2H, d, *J* = 8.9 Hz, 1,4-Disubstituephenyl), 7.59 (1H, s, Thiazole), 7.86 (2H, d, *J* = 8.5 Hz, 4-Cyanophenyl), 7.96 (1H, s, -CH=N-), 8.03 (2H, d, *J* = 8.5 Hz, 4-Cyanophenyl), 12.01 (1H, s, -NH). ^13^C-NMR (75 MHz, DMSO-*d*_6_): δ = 34.42, 45.58, 47.76, 107.58, 109.98, 115.90, 119.47, 125.51, 126.56, 127.99, 133.13, 139.32, 142.57, 149.27, 151.55, 169.13. ESI-MS (*m*/*z*): [M + H]^+^: 467.09.

##### 1-Methylsulphonyl-4-[{2-[4-(4-nitrophenyl)-1,3-thiazol-2-yl]hydrazinylidene}methyl]piperazine (**3e**)

Yield: 88%, Rf = 0.79, M.P. = 220–223 °C, FTIR (ATR, cm^−1^): 3305 (N-H), 2845 (C-H), 1504, 1334 (NO_2_), 819. ^1^H-NMR (300 MHz, DMSO-*d*_6_): δ = 2.92 (3H, s, -CH_3_), 3.23–3.26 (4H, m, piperazine), 3.34–3.37 (4H, m, piperazine), 7.02 (2H, d, *J* = 8.9 Hz, 1,4-Disubstituephenyl), 7.53 (2H, d, *J* = 8.9 Hz, 1,4-Disubstituephenyl), 7.67 (1H, s, Thiazole), 7.96 (1H, s, -CH=N-), 8.10 (2H, d, *J* = 9.0 Hz, 4-Nitrophenyl), 8.27 (2H, d, *J* = 9.0 Hz, 4-Nitrophenyl), 12.05 (1H, s, -NH). ^13^C-NMR (75 MHz, DMSO-*d*_6_): δ = 34.42, 45.58, 47.75, 108.60, 115.89, 124.55, 125.47, 126.77, 128.01, 141.22, 142.66, 146.63, 148.97, 151.57, 169.22. ESI-MS (*m*/*z*): [M + H]^+^: 487.08.

##### 1-Methylsulphonyl-4-[{2-[4-(4-fluorophenyl)-1,3-thiazol-2-yl]hydrazinylidene}methyl]piperazine (**3f**)

Yield: 79%, Rf = 0.31, M.P. = 190–193 °C, FTIR (ATR, cm^−1^): 3344 (N-H), 2841 (C-H), 817. ^1^H-NMR (300 MHz, DMSO-*d*_6_): δ = 2.92 (3H, s, -CH_3_), 3.23–3.25 (4H, m, piperazine), 3.33–3.35 (4H, m, piperazine), 6.98 (2H, d, *J* = 8.9 Hz, 1,4-Disubstituephenyl), 7.22–7.27 (2H, m, 4-Fluorophenyl + Thiazole), 7.49–7.52 (1H, m, 4-Fluorophenyl), 7.64 (2H, d, *J* = 8.9 Hz, 1,4-Disubstituephenyl), 7.83–7.86 (2H, m, 4-Fluorophenyl), 7.96 (1H, s, -CH=N-), 11.24 (1H, s, -NH). ^13^C-NMR (75 MHz, DMSO-*d*_6_): δ =34.42, 45.58, 47.64, 47.79, 103.41, 115.75 (*J* = 26.08 Hz), 125.20, 125.64, 127.92, 128.99, 142.19, 143.07, 151.49, 151.85, 162.04 (*J* = 244.24 Hz), 168.90, 177.84. ESI-MS (*m*/*z*): [M + H]^+^: 460.08.

##### 1-Methylsulphonyl-4-[{2-[4-(4-chlorophenyl)-1,3-thiazol-2-yl]hydrazinylidene}methyl]piperazine (**3g**)

Yield: 81%, Rf = 0.71, M.P. = 204–208 °C, FTIR (ATR, cm^−1^): 3300 (N-H), 2835 (C-H), 817. ^1^H-NMR (300 MHz, DMSO-*d*_6_): δ = 2.93 (3H, s, -CH_3_), 3.23–3.26 (4H, m, piperazine), 3.34–3.37 (4H, m, piperazine), 7.03 (2H, d, *J* = 8.9 Hz, 1,4-Disubstituephenyl), 7.36 (1H, s, Thiazole), 7.46 (2H, d, *J* = 8.6 Hz, 4-Chlorophenyl), 7.53 (2H, d, *J* = 8.9 Hz, 1,4-Disubstituephenyl), 7.87 (2H, d, *J* = 8.6 Hz, 4-Chlorophenyl), 7.95 (1H, s, -CH=N-), 11.98 (1H, s, -NH). ^13^C-NMR (75 MHz, DMSO-*d*_6_): δ = 34.34, 45.57, 47.78, 106.55, 115.95, 125.57, 126.04, 126.08, 126.50, 127.99, 138.82, 142.50, 149.30, 151.48, 169.07. ESI-MS (*m*/*z*): [M + H]^+^: 476.05.

##### 1-Methylsulphonyl-4-[{2-[4-(4-trifluoromethylphenyl)-1,3-thiazol-2-yl]hydrazinylidene}methyl]piperazine (**3h**)

Yield: 77%, Rf = 0.78, M.P. = 142–145 °C, FTIR (ATR, cm^−1^): 3304 (N-H), 2827 (C-H), 844. ^1^H-NMR (300 MHz, DMSO-*d*_6_): δ = 2.93 (3H, s, -CH_3_), 3.23–3.26 (4H, m, piperazine), 3.34–3.36 (4H, m, piperazine), 7.04 (2H, d, *J* = 8.9 Hz, 1,4-Disubstituephenyl), 7.52–7.55 (3H, m, 1,4-Disubstituephenyl + Thiazole), 7.76 (2H, d, *J* = 8.3 Hz, 4-Trifluoromethylphenyl), 7.96 (1H, s, -CH=N-), 8.06 (2H, d, *J* = 8.1 Hz, 4-Trifluoromethylphenyl), 12.06 (1H, s, -NH). ^13^C-NMR (75 MHz, DMSO-*d*_6_): δ = 34.42, 45.58, 47.64, 104.56, 115.58, 120.89, 125.20, 127.98, 128.99, 129.97, 131.96, 142.30, 143.07, 151.85, 177.84. ESI-MS (*m*/*z*): [M + H]^+^: 510.08.

### 4.3. Pharmacology

#### 4.3.1. Animals

Adult Balb/c male mice (aged 12–15 weeks, body weight 30–35 g), obtained from the Anadolu University Research Unit for Experimental Animals, Eskişehir, Turkey, were used in the study. The animals were housed in well-ventilated rooms with a 12/12 h dark/light cycle at a temperature of 24 ± 1 °C. The food in the cages was withdrawn 12 h before the experiments to avoid a possible food interference with the absorption of the test compounds. 

The experimental protocol of this research has been approved by the Local Ethical Committee on Animal Experimentation of Anadolu University, Eskişehir, Turkey. 

#### 4.3.2. Administration of the Test Compounds

The mice were divided into 10 groups of seven animals each. The test compounds were dissolved in sunflower oil and administered (p.o.) to animals at doses of 50 mg/kg in a volume of 0.1 mL [52]. Sunflower oil was used as a no-drug control and morphine sulphate (10 mg/kg, i.p.) was selected as a reference drug [40].

Measurements were taken 60 min after the administration of the test compounds or sunflower oil, and 30 min after the administration of morphine [53].

#### 4.3.3. Evaluation of the Antinociceptive Activity

##### Tail-Clip Test

A tail-clip test was used to assess the response of animals to mechanically induced noxious stimuli. A metal artery clamp that applies standardized pressure was placed 2–2.5 cm from the base of the tail and latency for turning and biting the clamp was recorded by a stopwatch [54]. A sensitivity test was performed before the test session, and mice that did not respond to the clip for 10 s were eliminated from the experiments. The cut-off time for this test was accepted as 10 s to avoid possible tail damage. Tail-clip tests were performed before and after the test compound administrations, with the prolongations in the reaction times being considered as a parameter for the antinociceptive effect [41].

##### Hot-Plate Test

A hot-plate test was used to assess the reaction of animals against thermal noxious stimuli. The hot-plate device consists of a heated surface kept at a constant temperature of 55 ± 1.0 °C (Ugo Basile, 7280, Varese, Italy). The animals were placed on the surface of the aluminum plate and pain thresholds were determined before and after the test compounds’ administrations. Paw licking or jumping latencies of each animal were recorded in seconds. In sensitivity tests, animals that failed to show a nociceptive response within 15 s were discarded from the experiments. The cut-off time for this test was accepted as 30 s to avoid an injury to the paws [40,55].

In both the tail-clip and hot-plate tests, analgesic efficacies of the test compounds were expressed as a percentage of the maximum possible effect (MPE %) using the following equation:

MPE % = ((post-drug latency − pre-drug latency)/(cut-off time − pre-drug latency)) × 100 (1)

##### Acetic Acid-Induced Writhing Test

The acetic acid-induced writhing test was used to evaluate the responses of animals to chemically induced noxious stimuli elicited by i.p. injection of 0.6% *v*/*v* acetic acid in a volume of 0.1 mL/10 g. The mice were then placed in transparent boxes and the number of writhing behaviors was recorded for 10 minutes, 5 minutes after acetic acid injections [53,56]. Reductions in the number of writhing behaviors were considered as evidence for the antinociceptive effect. The inhibition percentage of the nociceptive behavior was calculated according to the following equation:

Inhibition% = ([(mean number of writhes (control) − mean number of writhes (treatment)]/mean number of writhes (control)) × 100 (2)

##### Mechanistic Studies

The possible involvement of opioid receptors in the antinociceptive effects of test compounds was examined by mechanistic studies using a non-selective opioid receptor antagonist, naloxone. For antagonism studies, mice were pre-treated with naloxone at a dose of 5.48 mg/kg 15 min before the administrations of test compounds [40,41]. Then, experiments were carried out as explained previously.

#### 4.3.4. Evaluation of the Motor Activity

##### Rota-Rod Test

The Rota-Rod test device (Ugo Basile, 47600, Varese, Italy) was used to evaluate the possible effects of test compounds on the motor coordination of animals. Before experiments, mice were trained on the rotating rod of the apparatus set at 16 rpm for 3 consecutive days. Animals that could stay on the rotating mill for more than 180 s were used for the tests. The falling time of the mice was considered as a parameter for motor coordination. The cut-off time for this test was accepted as 10 min [40,41,57].

#### 4.3.5. Statistical Analysis

Statistical evaluation of the experimental data was carried out using GraphPad Prism ver. 8.4.3 software (GraphPad Software, La Jolla, CA, USA). The data used in the statistical analyses were acquired from seven animals in each group. The differences between experimental groups were determined by one-way analysis of variance (ANOVA) followed by a post-hoc Tukey’s test. The results were presented as mean ± standard error of the mean (SEM) and considered statistically significant when *p* < 0.05.

GraphPad Prism ver. 8.4.3 software were used for creating the figures.

### 4.4. Molecular Docking Studies

Molecular docking studies were performed using an in silico procedure to define the binding modes of obtained compounds in active regions of opioid receptors. X-ray crystal structures of µ-opioid receptor (PDB ID: 5C1M) [33], δ-opioid receptor (PDB ID: 4N6H) [34], and ĸ-opioid receptor (PDB ID: 6B73) [35] were retrieved from the Protein Data Bank server (www.pdb.org, accessed date 28 April 2021). Active conformations of these receptors were used. 

The structures of proteins were built using the Schrödinger Maestro [58] interface and were then submitted to the Protein Preparation Wizard protocol of the Schrödinger Suite 2016 Update 2 [59]. The ligands were prepared using LigPrep 3.8 [60] to correctly assign the protonation states at pH 7.4 ± 1.0, as well as the atom types. Bond orders were assigned, and hydrogen atoms were added to the structures. The grid generation was formed using the Glide 7.1 [61] program and docking runs were performed with standard precision docking mode (SP). 

## 5. Conclusions

To the best of our knowledge, this is the first study showing that molecules designed to carry thiazole and piperazine moieties together on their structures have convenient pharmacokinetic profiles and show notable antinociceptive efficacies mediated by the opioid receptors at the spinal, supraspinal, and peripheral sites.

## Figures and Tables

**Table 1:** Some physicochemical parameters of the compounds **3a**–**3h** used in the prediction of ADME profiles.

Compounds	R	MW	TPSA	logP	AHB	DHB	MV	Vio
**3a**	-H	444.58	77.90	3.33	7	1	379.00	0
**3b**	-CH_3_	455.61	77.90	3.77	7	1	395.56	0
**3c**	-OCH_3_	471.61	87.14	3.38	8	1	404.55	0
**3d**	-CN	466.59	101.69	3.08	8	1	395.86	0
**3e**	-NO_2_	486.58	123.72	3.29	10	1	402.34	0
**3f**	-F	459.57	77.90	3.49	7	1	383.94	0
**3g**	-Cl	476.03	77.90	4.00	7	1	392.54	0
**3h**	-CF_3_	509.58	77.90	4.22	7	1	410.30	1

**Table 2:** Effects of test compounds (50 mg/kg, p.o.) and morphine (10 mg/kg, i.p.) on protection (%) values of mice in the acetic acid-induced writhing test.

Treatment	Protection %
Control	-
Morphine	76.69
**3a**	48.54
**3b**	44.17
**3c**	47.57
**3d**	24.27
**3e**	19.90
**3f**	63.10
**3g**	66.99
**3h**	29.61

**Table 3:** Interaction sites of opioidergic receptor subtypes with the test compounds.

Compound	Receptor *	H-Bond	π-π Interaction	Salt Bridge	π-Cation Interaction
**3a**	MOR		His54		
	DOR	Cys198	Trp274, Tyr308		
	KOR		Try139, Trp287		
**3b**	MOR	Lys303	Tyr148		
	DOR	Lys214, Asp128	Tyr129		
	KOR		Trp287		
**3c**	MOR	Lys303, Asp147	Tyr148		
	DOR				
	KOR				
**3d**	MOR				
	DOR				
	KOR		His291		
**3e**	MOR			Lys303	
	DOR				His278
	KOR		Try139	Lys227, Glu297	Lys227, Tyr312
**3f**	MOR	Lys303, Asp147			
	DOR	Cys198	Trp274, Tyr308		
	KOR		His291, Trp287		
**3g**	MOR	Lys303, Asp147	Tyr148		
	DOR	Asp128			
	KOR		Tyr139		
**3h**	MOR		His54		
	DOR				
	KOR		His291		

* Receptor crystals retrieved from the protein data bank. The PDBIDs for MOR, DOR, and KOR were 5C1M, 4N6H, and 6B73, respectively.

## Data Availability

All relevant data are included within the article or Supplementary Materials. The raw data are available upon request from the corresponding author.

## Supplementary Materials

The following are available online. Figure S1. IR spectra of compound **3a**. Figure S2. ^1^H-NMR spectra of compound **3a**. Figure S3. ^13^C-NMR spectra of compound **3a**. Figure S4. LCMSMS spectra of compound **3a**. Figure S5. IR spectra of compound **3b**. Figure S6. ^1^H-NMR spectra of compound **3b**. Figure S7. ^13^C-NMR spectra of compound **3b**. Figure S8. LCMSMS spectra of compound **3b**. Figure S9. IR spectra of compound **3c**. Figure S10. ^1^H-NMR spectra of compound **3c**. Figure S11. ^13^C-NMR spectra of compound **3c**. Figure S12. LCMSMS spectra of compound **3c**. Figure S13. IR spectra of compound **3d**. Figure S14. 1H-NMR spectra of compound **3d**. Figure S15. 13C-NMR spectra of compound **3d**. Figure S16. LCMSMS spectra of compound **3d**. Figure S17. IR spectra of compound **3e**. Figure S18. ^1^H-NMR spectra of compound **3e**. Figure S19. ^13^C-NMR spectra of compound **3e.** Figure S20. LCMSMS spectra of compound **3e**. Figure S21. IR spectra of compound **3f.** Figure S22. ^1^H-NMR spectra of compound **3f**. Figure S23. ^13^C-NMR spectra of compound **3f.** Figure S24. LCMSMS spectra of compound **3f**. Figure S25. IR spectra of compound **3g.** Figure S26. ^1^H-NMR spectra of compound **3g**. Figure S27. ^13^C-NMR spectra of compound **3g**. Figure S28. LCMSMS spectra of compound **3g**. Figure S29. IR spectra of compound **3h.** Figure S30. ^1^H-NMR spectra of compound **3h**. Figure S31. ^13^C-NMR spectra of compound **3h**. Figure S32. LCMSMS spectra of compound **3h.** Figure S33. The two-dimensional interacting mode of compound **3a** in the active region of µ-opioid receptor (PDB Code: 5C1M). Figure S34. The three-dimensional interacting mode of compound **3a** in the active region of µ -opioid receptor. The ligand and significant residues of the active site of the receptor are presented by a tube model colored with purple and white, respectively (PDB Code: 5C1M). Figure S35. The two-dimensional interacting mode of compound **3b** in the active region of µ-opioid receptor. (PDB Code: 5C1M). Figure S36. The three-dimensional interacting mode of compound **3b** in the active region of µ-opioid receptor. The ligand and significant residues of the active site of the receptor are presented by a tube model colored with orange and white, respectively (PDB Code: 5C1M). Figure S37. The two-dimensional interacting mode of compound **3c** in the active region of µ-opioid receptor. (PDB Code: 5C1M). Figure S38. The three-dimensional interacting mode of compound **3c** in the active region of µ-opioid receptor. The ligand and significant residues of the active site of the receptor are presented by a tube model colored with blue and white, respectively (PDB Code: 5C1M). Figure S39. The two-dimensional interacting mode of compound **3d** in the active region of µ-opioid receptor. (PDB Code: 5C1M). Figure S40. The three-dimensional interacting mode of compound **3d** in the active region of µ-opioid receptor. The ligand and significant residues of the active site of the receptor are presented by a tube model colored with red and white, respectively (PDB Code: 5C1M). Figure S41. The two-dimensional interacting mode of compound **3e** in the active region of µ-opioid receptor. (PDB Code: 5C1M). Figure S42. The three-dimensional interacting mode of compound **3e** in the active region of µ-opioid receptor. The ligand and significant residues of the active site of the receptor are presented by a tube model colored with red and white, respectively (PDB Code: 5C1M). Figure S43. The two-dimensional interacting mode of compound **3h** in the active region of µ-opioid receptor. (PDB Code: 5C1M). Figure S44. The three-dimensional interacting mode of compound **3h** in the active region of µ-opioid receptor. The ligand and significant residues of the active site of the receptor are presented by a tube model colored with red and white, respectively (PDB Code: 5C1M). Figure S45. The two-dimensional interacting mode of compound **3a** in the active region of δ-opioid receptor. (PDB Code: 4N6H). Figure S46. The three-dimensional interacting mode of compound **3a** in the active region of δ-opioid receptor. The ligand and significant residues of the active site of the receptor are presented by a tube model colored with blue and white, respectively (PDB Code: 4N6H). Figure S47. The two-dimensional interacting mode of compound **3b** in the active region of δ-opioid receptor. (PDB Code: 4N6H). Figure S48. The three-dimensional interacting mode of compound **3b** in the active region of δ-opioid receptor. The ligand and significant residues of the active site of the receptor are presented by a tube model colored with blue and white, respectively (PDB Code: 4N6H). Figure S49. The two-dimensional interacting mode of compound **3c** in the active region of δ-opioid receptor. (PDB Code: 4N6H). Figure S50. The three-dimensional interacting mode of compound **3c** in the active region of δ-opioid receptor. The ligand and significant residues of the active site of the receptor are presented by a tube model colored with green and white, respectively (PDB Code: 4N6H). Figure S51. The two-dimensional interacting mode of compound **3d** in the active region of δ-opioid receptor. (PDB Code: 4N6H). Figure S52. The three-dimensional interacting mode of compound **3d** in the active region of δ-opioid receptor. The ligand and significant residues of the active site of the receptor are presented by a tube model colored with red and white, respectively (PDB Code: 4N6H). Figure S53. The two-dimensional interacting mode of compound **3e** in the active region of δ-opioid receptor. (PDB Code: 4N6H). Figure S54. The three-dimensional interacting mode of compound **3e** in the active region of δ-opioid receptor. The ligand and significant residues of the active site of the receptor are presented by a tube model colored with red and white, respectively (PDB Code: 4N6H). Figure S55. The two-dimensional interacting mode of compound **3h** in the active region of δ-opioid receptor. (PDB Code: 4N6H). Figure S56. The three-dimensional interacting mode of compound **3h** in the active region of δ-opioid receptor. The ligand and significant residues of the active site of the receptor are presented by a tube model colored with red and white, respectively (PDB Code: 4N6H). Figure S57. The two-dimensional interacting mode of compound **3a** in the active region of ĸ-opioid receptor. (PDB Code: 6B73). Figure S58. The three-dimensional interacting mode of compound **3a** in the active region of ĸ-opioid receptor. The ligand and significant residues of the active site of the receptor are presented by a tube model colored with green and white, respectively (PDB Code: 6B73). Figure S59. The two-dimensional interacting mode of compound **3b** in the active region of ĸ-opioid receptor. (PDB Code: 6B73). Figure S60. The three-dimensional interacting mode of compound **3b** in the active region of ĸ-opioid receptor. The ligand and significant residues of the active site of the receptor are presented by a tube model colored with pink and white, respectively (PDB Code: 6B73). Figure S61. The two-dimensional interacting mode of compound **3c** in the active region of ĸ-opioid receptor. (PDB Code: 6B73). Figure S62. The three-dimensional interacting mode of compound **3c** in the active region of ĸ-opioid receptor. The ligand and significant residues of the active site of the receptor are presented by a tube model colored with orange and white, respectively (PDB Code: 6B73). Figure S63. The two-dimensional interacting mode of compound **3d** in the active region of ĸ-opioid receptor. (PDB Code: 6B73). Figure S64. The three-dimensional interacting mode of compound **3d** in the active region of ĸ-opioid receptor. The ligand and significant residues of the active site of the receptor are presented by a tube model colored with yellow and white, respectively (PDB Code: 6B73). Figure S65. The two-dimensional interacting mode of compound **3e** in the active region of ĸ-opioid receptor. (PDB Code: 6B73). Figure S66. The three-dimensional interacting mode of compound **3e** in the active region of ĸ-opioid receptor. The ligand and significant residues of the active site of the receptor are presented by a tube model colored with blue and white, respectively (PDB Code: 6B73). Figure S67. The two-dimensional interacting mode of compound **3h** in the active region of ĸ-opioid receptor. (PDB Code: 6B73). Figure S68. The three-dimensional interacting mode of compound **3h** in the active region of ĸ-opioid receptor. The ligand and significant residues of the active site of the receptor are presented by a tube model colored with pink and white, respectively (PDB Code: 6B73).

## Sample Availability

Samples of the compounds are available from the authors.
