# Supplementary material for: Synthesis and Antinociceptive Effect of Some Thiazole-Piperazine Derivatives: Involvement of Opioidergic System in the Activity
Source: Molecules. 2021 Jun 2;26(11):3350. doi: 10.3390/molecules26113350 (PMC8199615; doi:10.3390/molecules26113350)
Supplement: Supplementary file 1 [file molecules-26-03350-s001.zip › molecules-1203253-SI.pdf]

DOPNALAB

Item	Value
Acquired Date&Time	13.04.2018 14:57:46
Acquired by	System Administrator
Filename	C:\Users\dopnalab\Desktop\derya\ahmet osman tokay\GS-BOS1.ispd
Spectrum name	GS-BOS1
Sample name	GS-BOS
Sample ID	
Option	
Comment	
No. of Scans	10
Resolution	4 [cm-1]
Apodization	Happ-Genzel

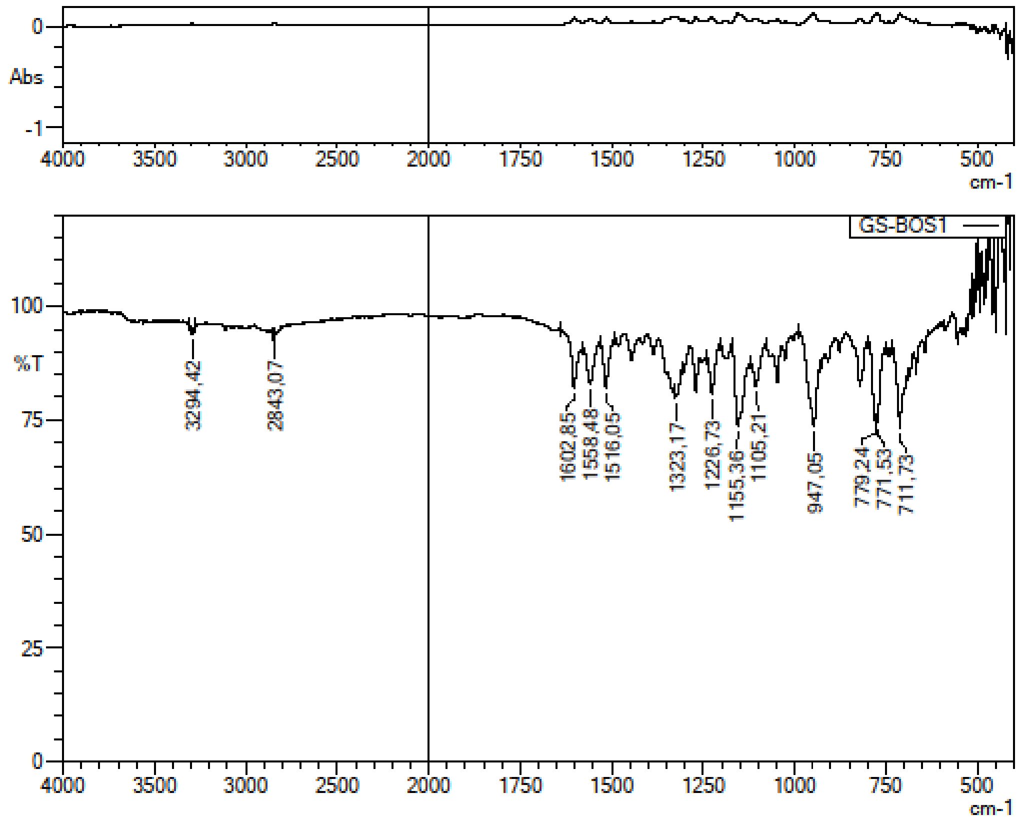

Figure S1. IR spectra of compound **3a**.

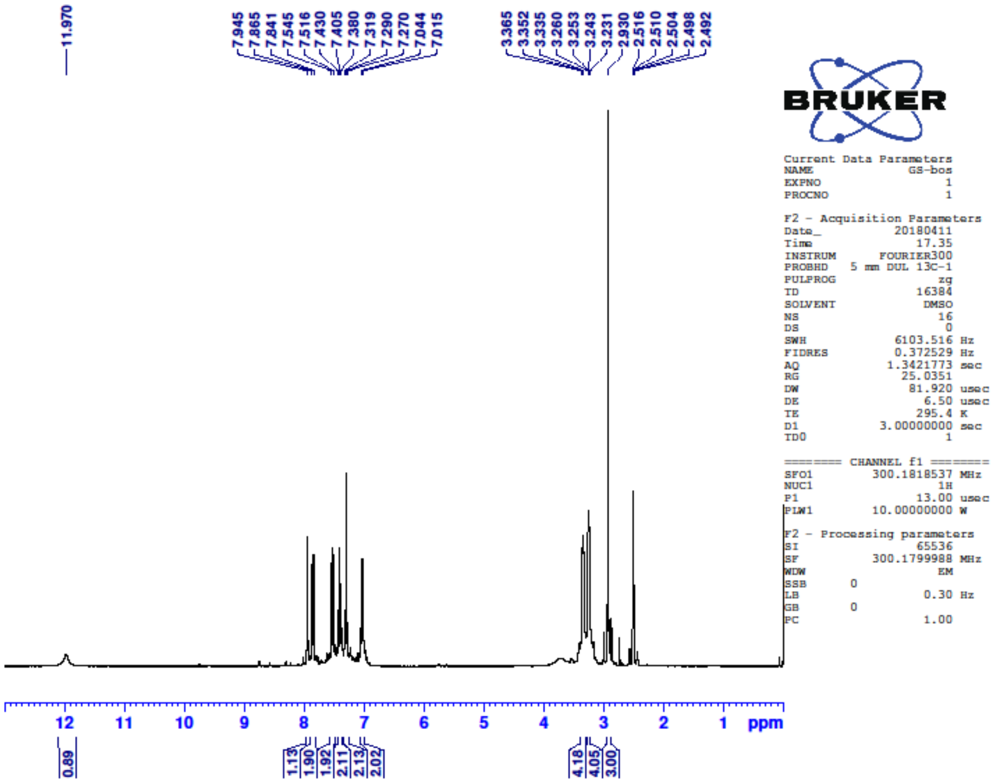

Figure S2. ^{1}H-NMR spectra of compound **3a**.

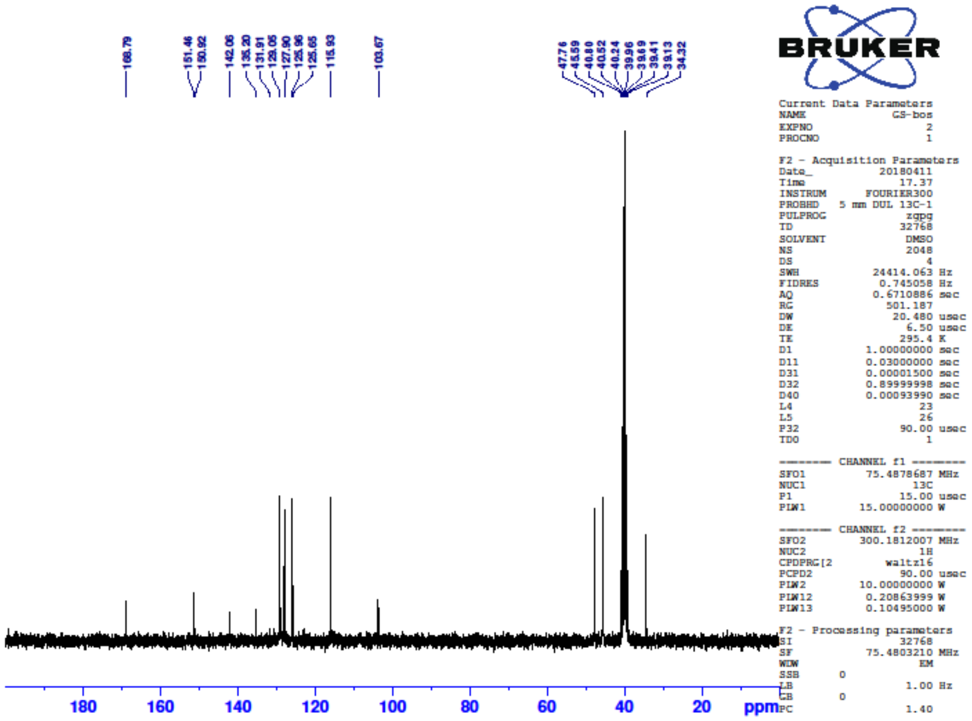

Figure S3. ^{13}C-NMR spectra of compound **3a**.

==== Shimadzu LCMSsolution Analysis Report ====

Acquired by	: Admin
Sample Name	: GS-Bos
Sample ID	:
Vail #	: 5
Injection Volume	: 0.3 uL
Data File Name	: GS-Bos_19.lcd
Method File Name	: genel.lcm
Batch File Name	: batch.lcb
Report File Name	: DefaultLCMS.lcr
Data Acquired	: 19.04.2018 12:52:35
Data Processed	: 19.04.2018 13:03:39

<Chromatogram>

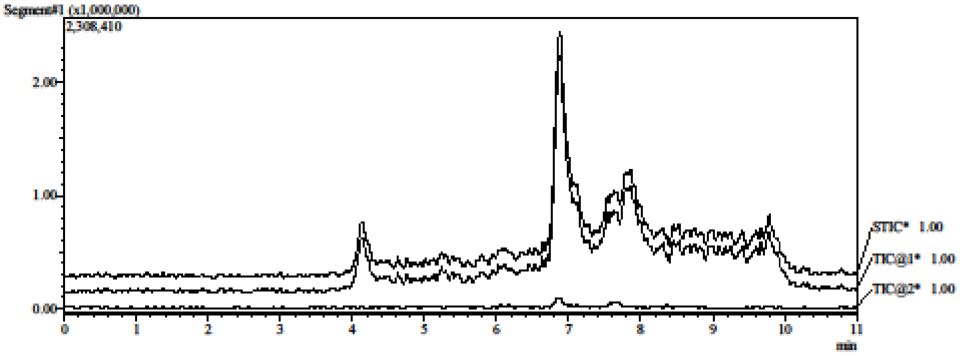

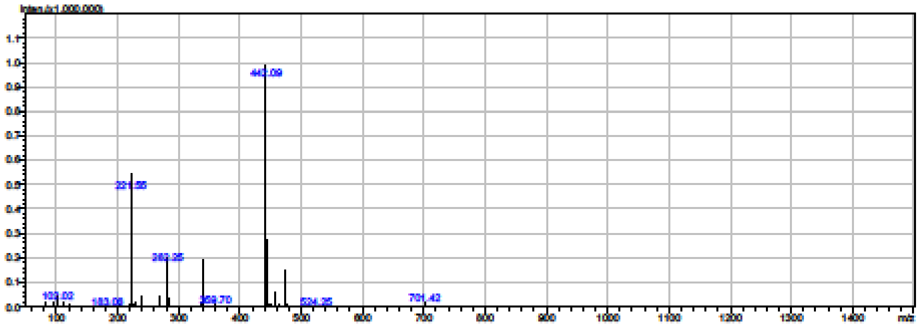

Figure S4. LCMSMS spectra of compound **3a**.

DOPNALAB

	Item	Value
	Acquired Date&Time	13.04.2018 15:02:22
	Acquired by	System Administrator
	Filename	C:\Users\dopnalab\Desktop\derya\ahmet osman tokay\GS-ME1.ispd
	Spectrum name	GS-ME1
	Sample name	GS-ME
	Sample ID	
	Option	
	Comment	
	No. of Scans	10
	Resolution	4 [cm-1]
	Apodization	Happ-Genzel

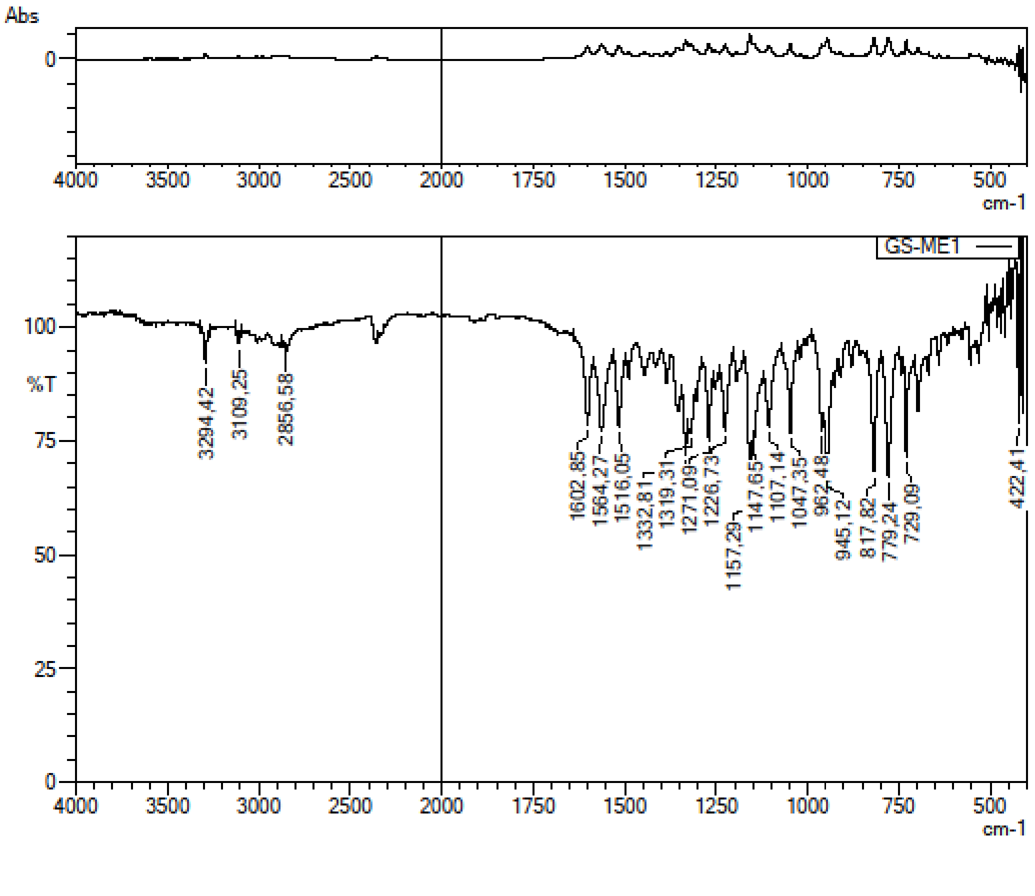

Figure S5. IR spectra of compound **3b**.

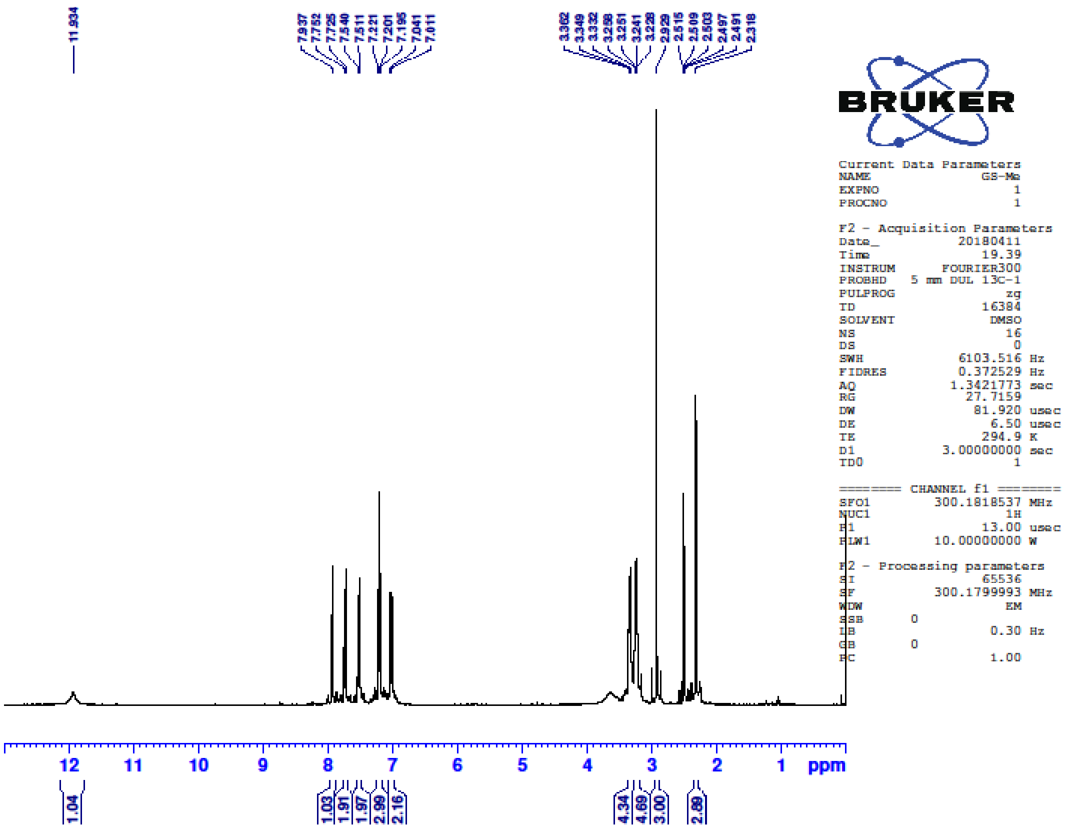

Figure S6. ^{1}H-NMR spectra of compound **3b**.

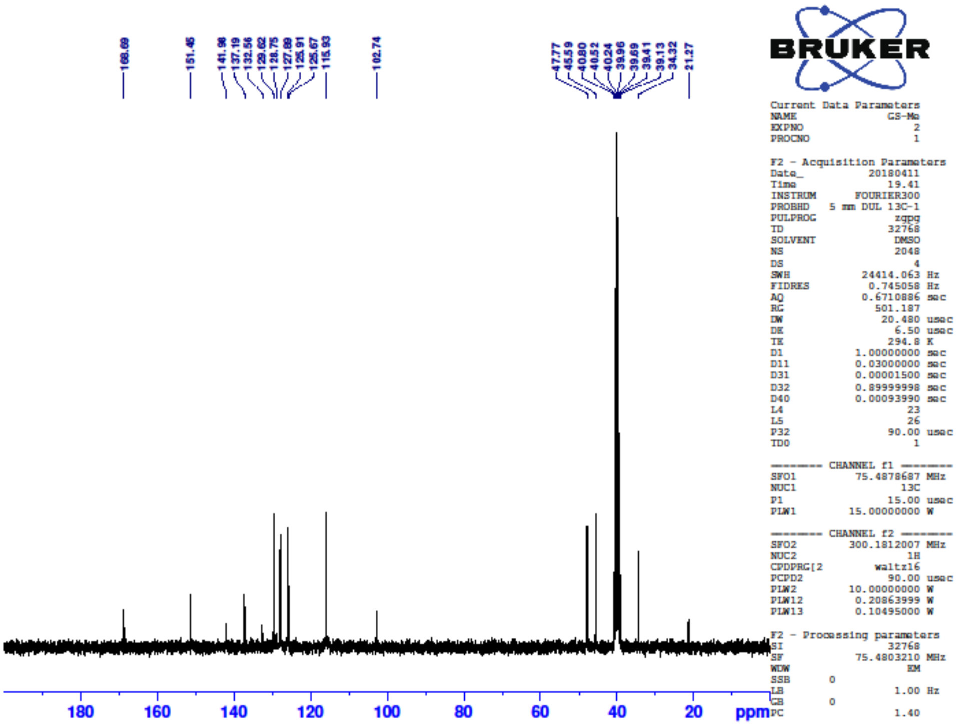

Figure S7. ^{13}C-NMR spectra of compound **3b**.

==== Shimadzu LCMSsolution Analysis Report ====

Acquired by	: Admin
Sample Name	: GS-Me
Sample ID	:
Vail #	: 6
Injection Volume	: 0.3 uL
Data File Name	: GS-Me_20.lcd
Method File Name	: genel.lcm
Batch File Name	: batch.lcb
Report File Name	: DefaultLCMS.lcr
Data Acquired	: 19.04.2018 13:04:10
Data Processed	: 19.04.2018 13:15:12

<Chromatogram>

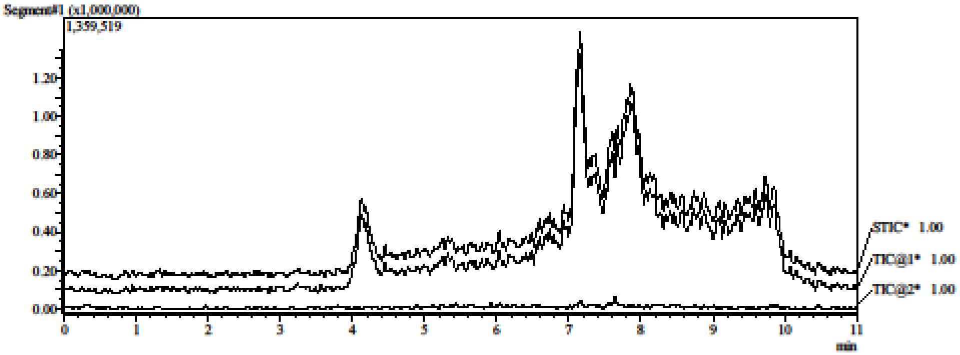

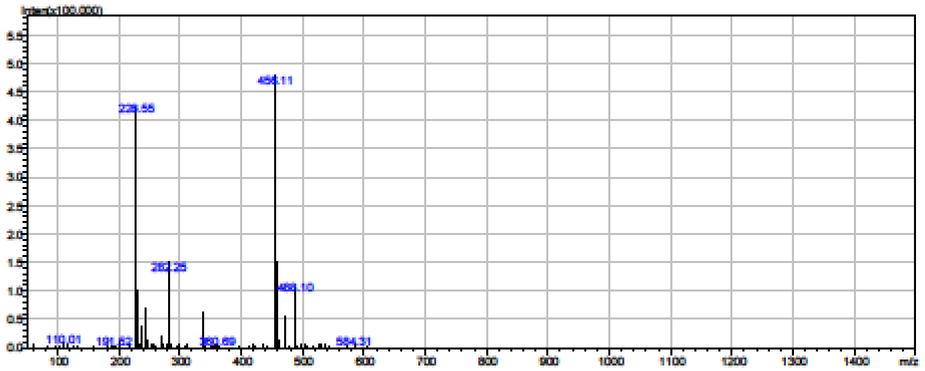

Figure S8. LCMSMS spectra of compound **3b**.

DOPNALAB

Item	Value
Acquired Date&Time	13.04.2018 15:04:31
Acquired by	System Administrator
Filename	C:\Users\dopnalab\Desktop\derya\ahmet osman tokay\GS-OME1.ispd
Spectrum name	GS-OME1
Sample name	GS-OME
Sample ID	
Option	
Comment	
No. of Scans	10
Resolution	4 [cm-1]
Apodization	Happ-Genzel

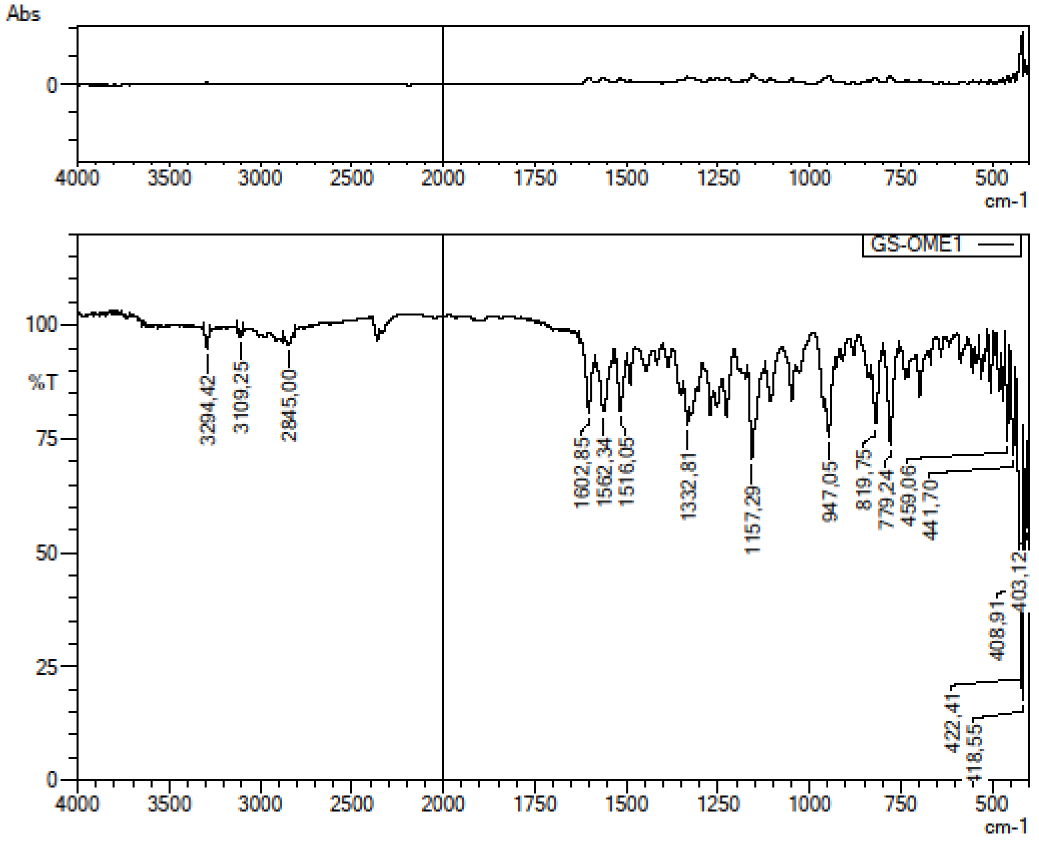

Figure S9. IR spectra of compound **3c**.

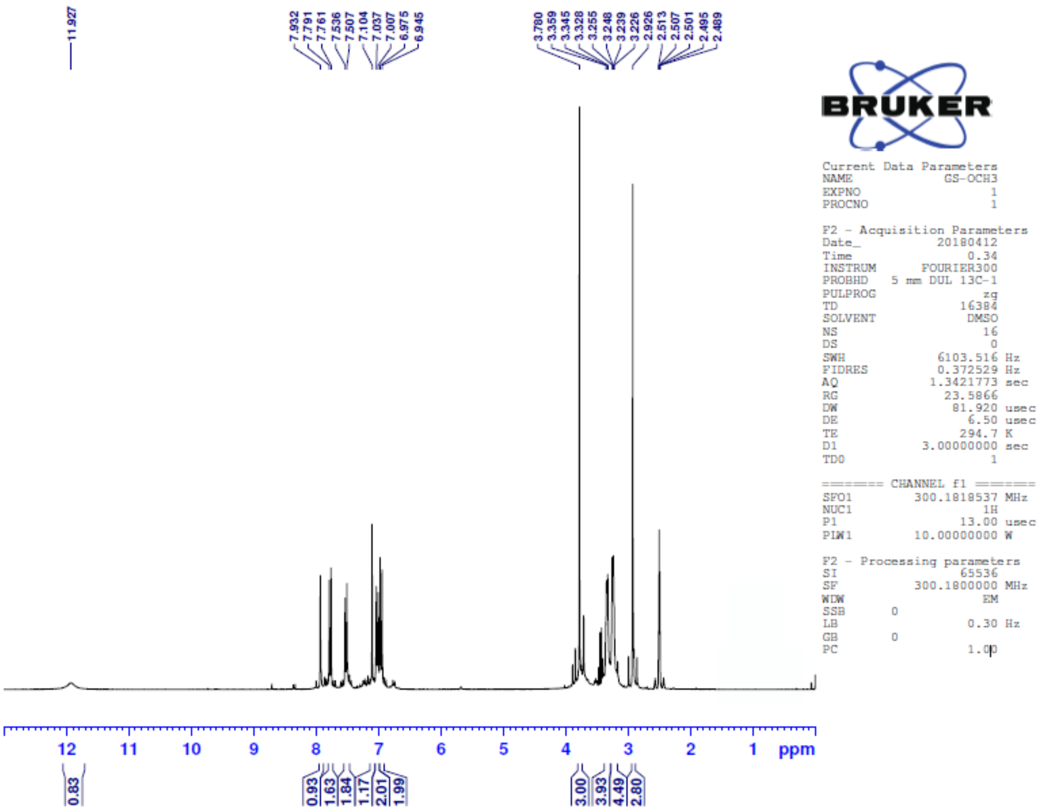

Figure S10. ^{1}H-NMR spectra of compound **3c**.

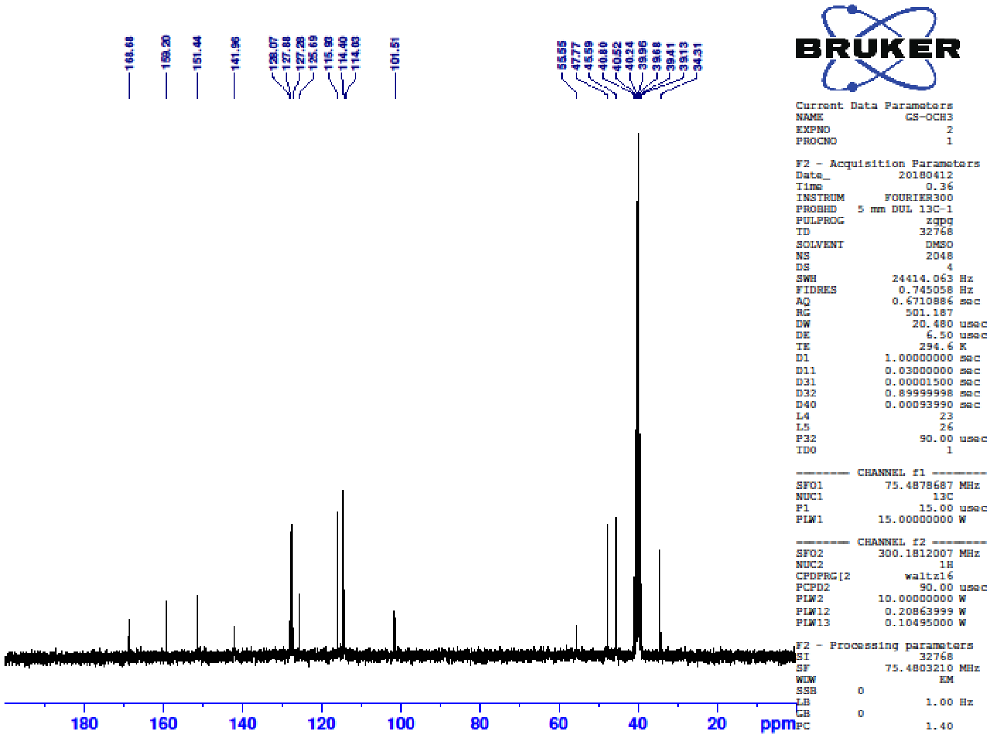

Figure S11. ^{13}C-NMR spectra of compound **3c**.

==== Shimadzu LCMSsolution Analysis Report ====

Acquired by	: Admin
Sample Name	: GS-OCH3
Sample ID	:
Vail #	: 1
Injection Volume	: 0.3 uL
Data File Name	: GS-OCH3_15.lcd
Method File Name	: genel.lcm
Batch File Name	: batch.lcb
Report File Name	: DefaultLCMS.lcr
Data Acquired	: 19.04.2018 12:06:21
Data Processed	: 19.04.2018 12:17:24

<Chromatogram>

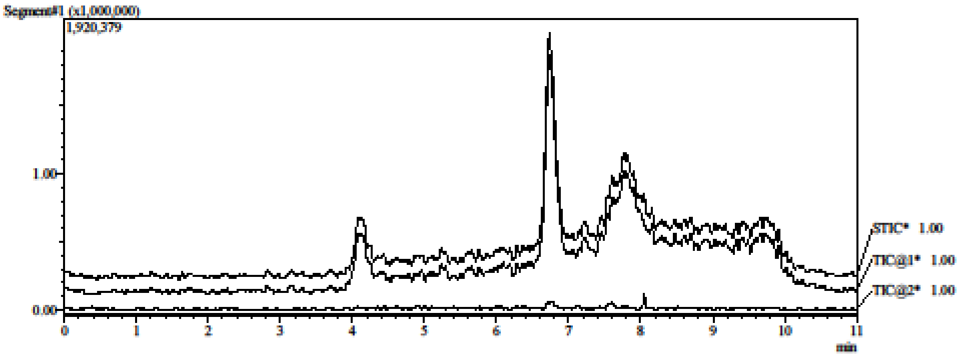

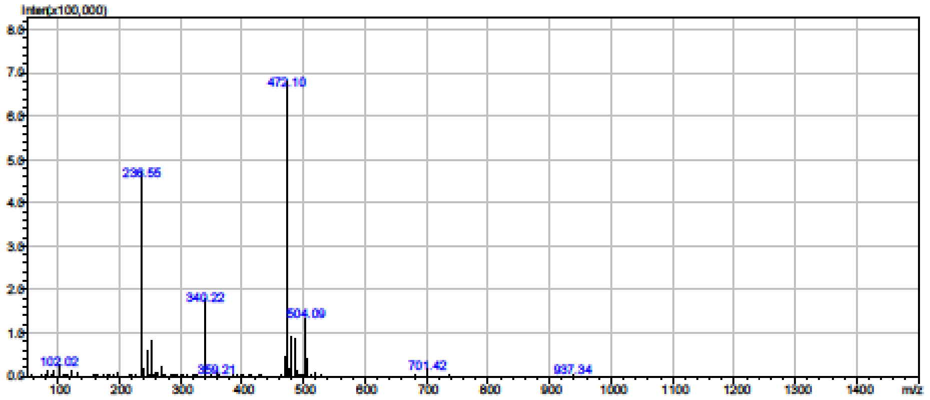

Figure S12. LCMSMS spectra of compound **3c**.

DOPNALAB

Item	Value
Acquired Date&Time	13.04.2018 15:09:08
Acquired by	System Administrator
Filename	C:\Users\dopnalab\Desktop\derya\ahmet osman tokay\GS-CN1.ispd
Spectrum name	GS-CN1
Sample name	GS-CN
Sample ID	
Option	
Comment	
No. of Scans	10
Resolution	4 [cm-1]
Apodization	Happ-Genzel

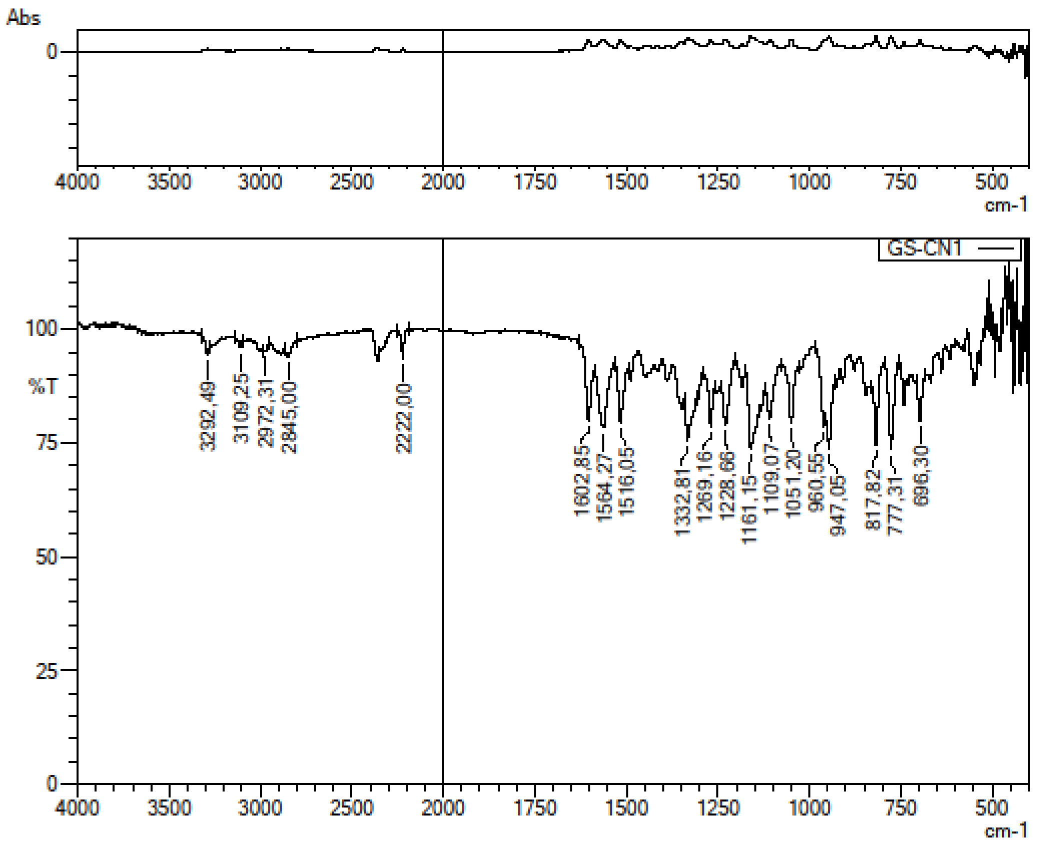

Figure S13. IR spectra of compound **3d**.

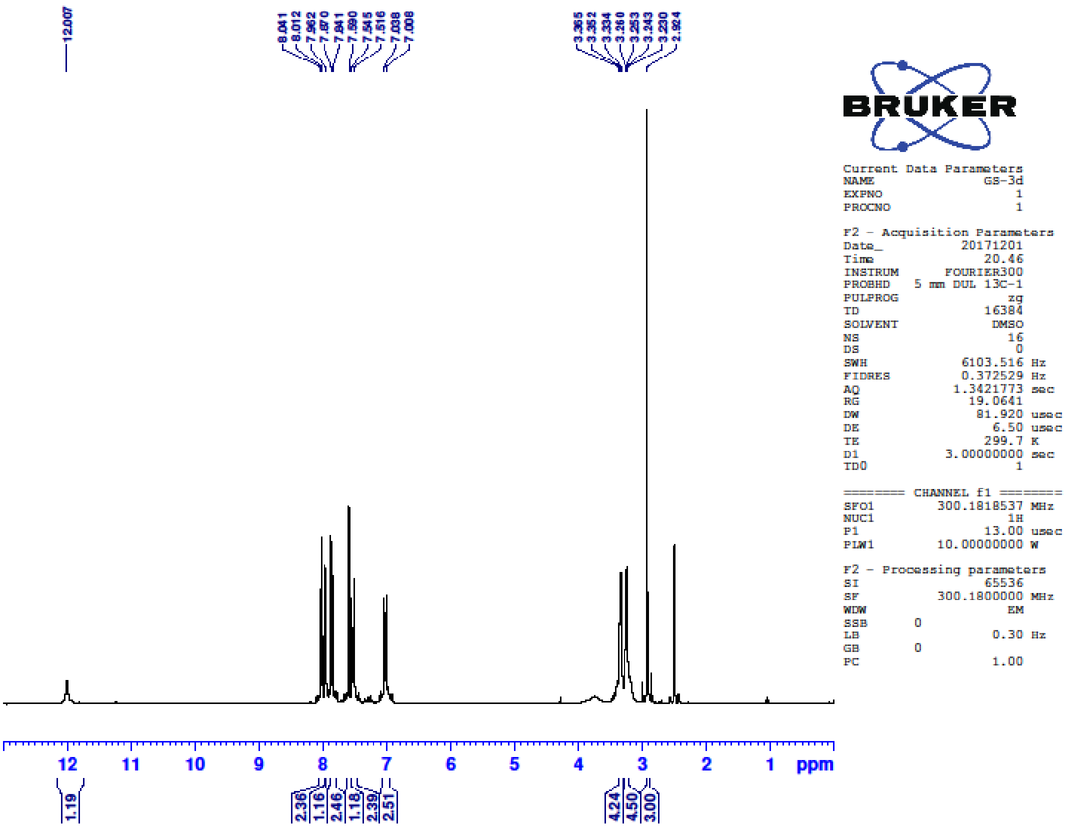

Figure S14. ^{1}H-NMR spectra of compound **3d**.

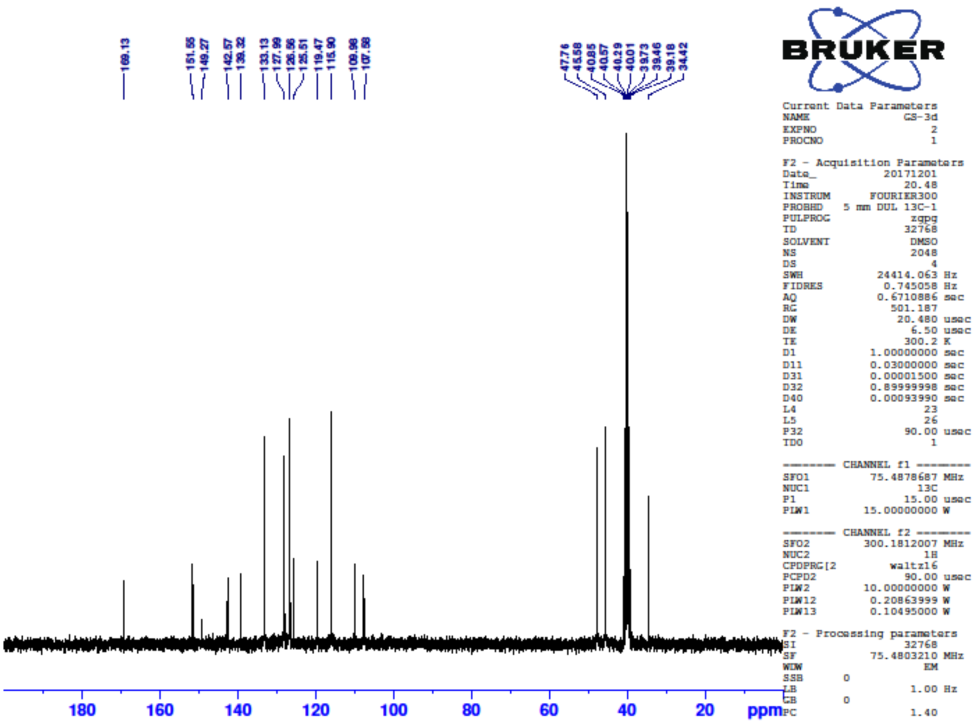

Figure S15. ^{13}C-NMR spectra of compound **3d**.

==== Shimadzu LCMSsolution Analysis Report ====

Acquired by	: Admin
Sample Name	: GS-CN
Sample ID	:
Vail #	: 7
Injection Volume	: 0.3 uL
Data File Name	: GS-CN_21.lcd
Method File Name	: genel.lcm
Batch File Name	: batch.lcb
Report File Name	: DefaultLCMS.lcr
Data Acquired	: 19.04.2018 13:15:42
Data Processed	: 19.04.2018 13:26:44

<Chromatogram>

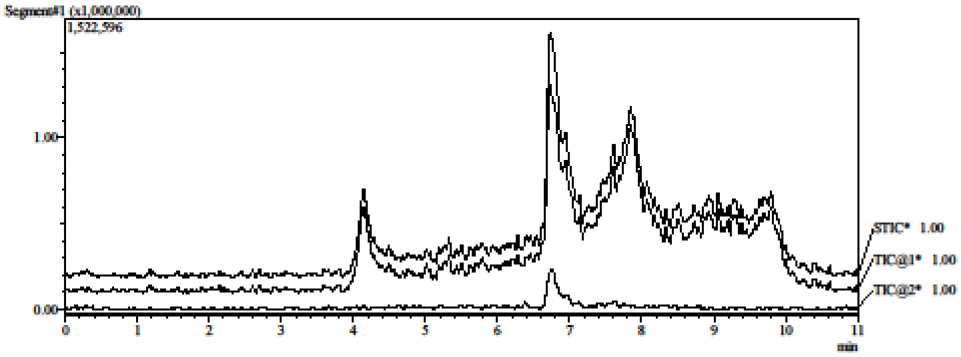

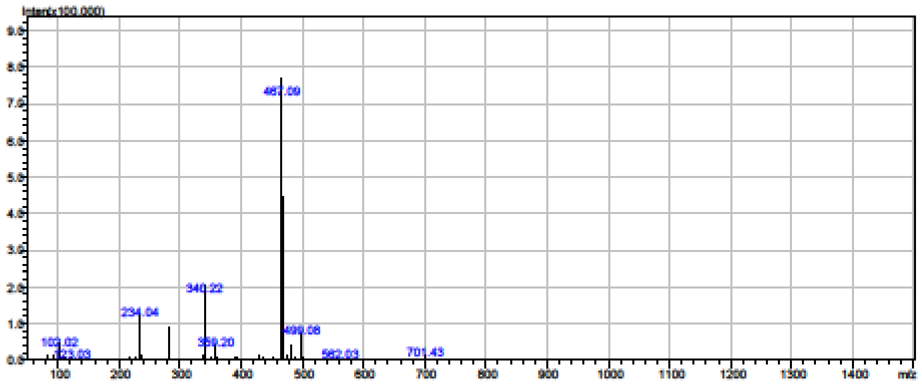

Figure S16. LCMSMS spectra of compound **3d**.

DOPNALAB

Item	Value
Acquired Date&Time	13.04.2018 15:12:32
Acquired by	System Administrator
Filename	C:\Users\dopnalab\Desktop\derya\ahmet osman tokay\GS-NO21.ispd
Spectrum name	GS-NO21
Sample name	GS-NO2
Sample ID	
Option	
Comment	
No. of Scans	10
Resolution	4 [cm-1]
Apodization	Happ-Genzel

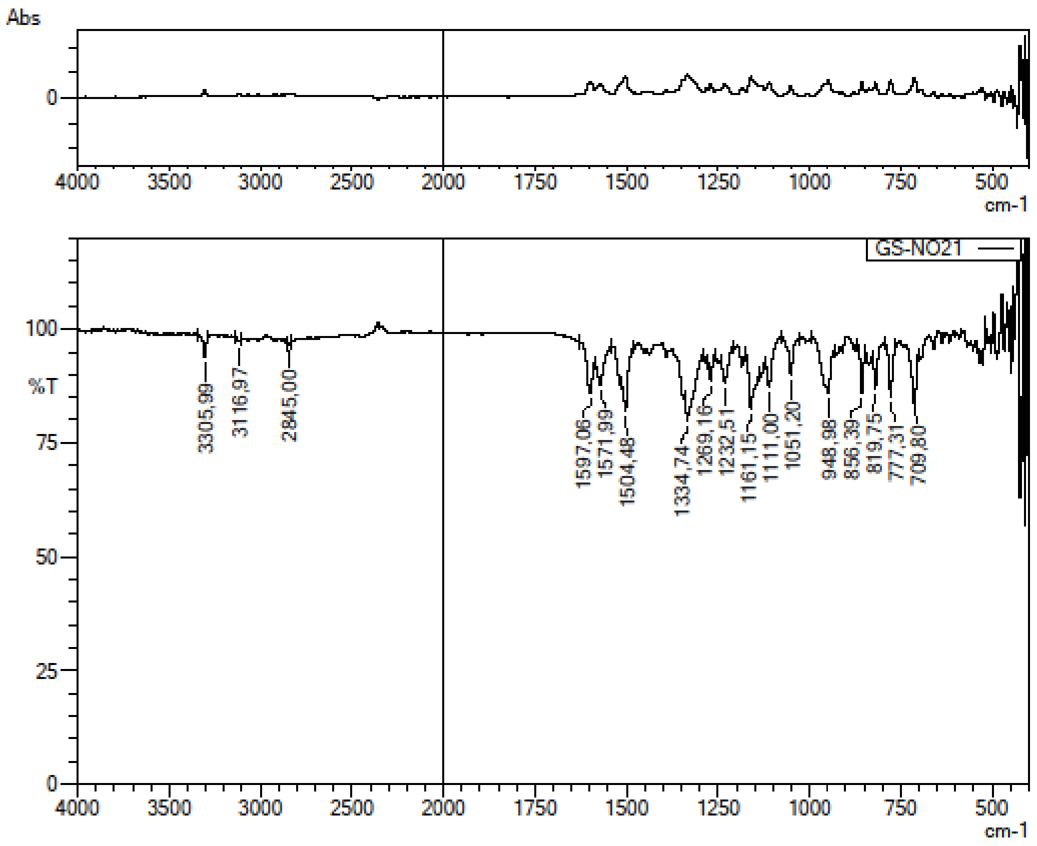

Figure S17. IR spectra of compound **3e**.

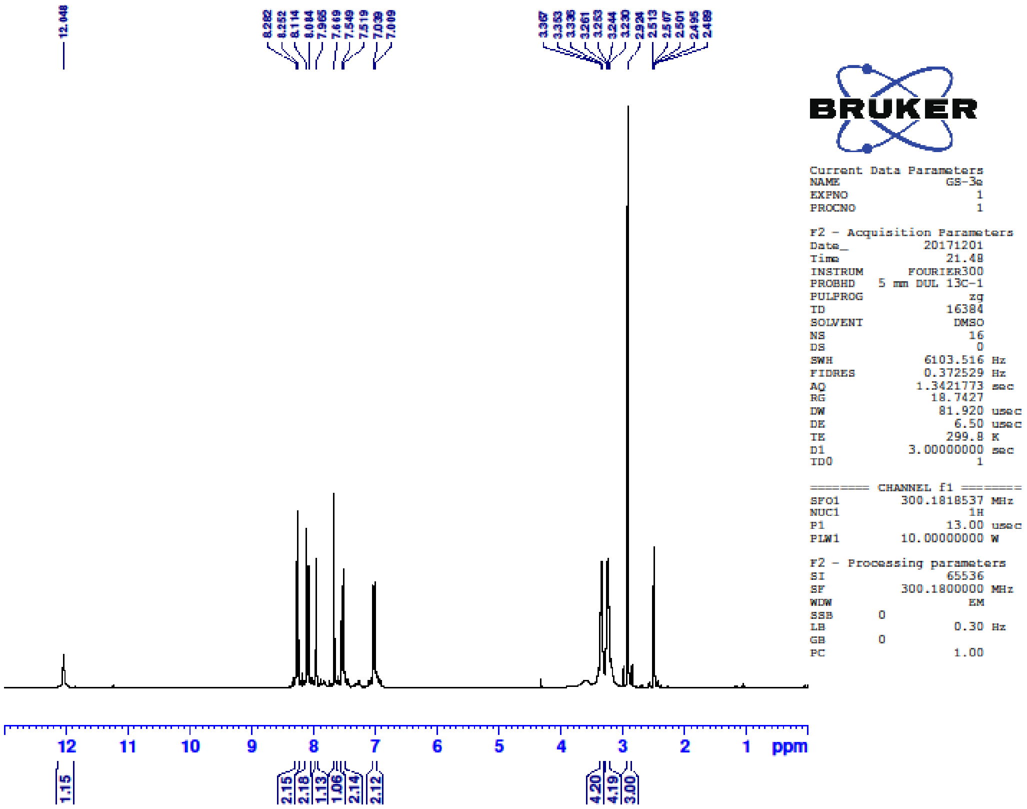

Figure S18. ^{1}H-NMR spectra of compound **3e**.

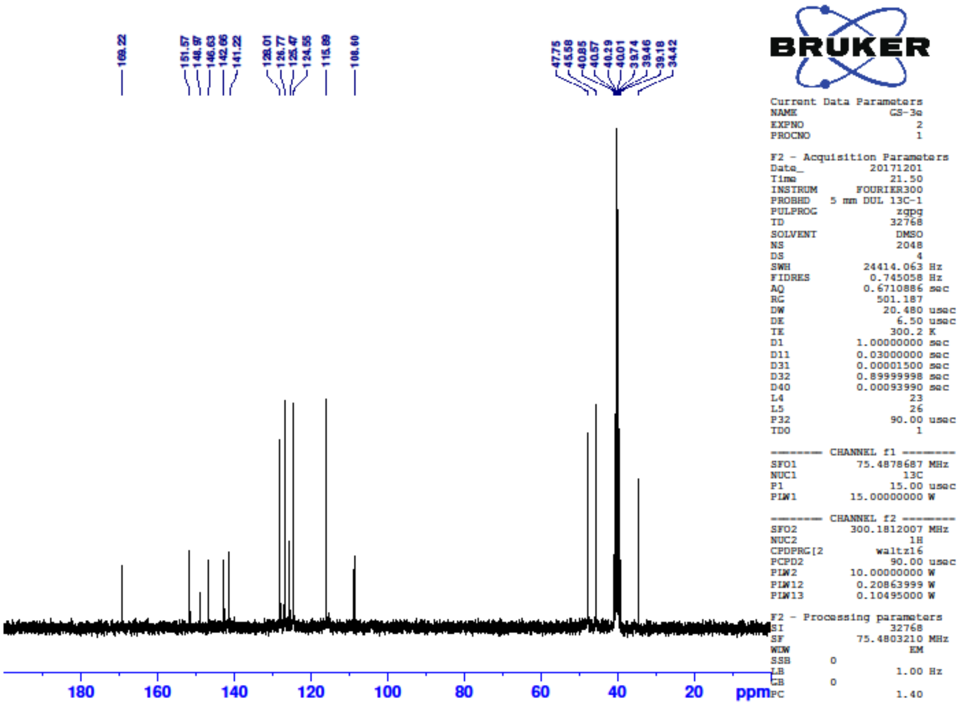

Figure S19. ^{13}C-NMR spectra of compound **3e**.

==== Shimadzu LCMSsolution Analysis Report ====

Acquired by	: Admin
Sample Name	: GS-NO2
Sample ID	:
Vail #	: 2
Injection Volume	: 0.3 uL
Data File Name	: GS-NO2_16.lcd
Method File Name	: genel.lcm
Batch File Name	: batch.lcb
Report File Name	: DefaultLCMS.lcr
Data Acquired	: 19.04.2018 12:17:55
Data Processed	: 19.04.2018 12:28:59

<Chromatogram>

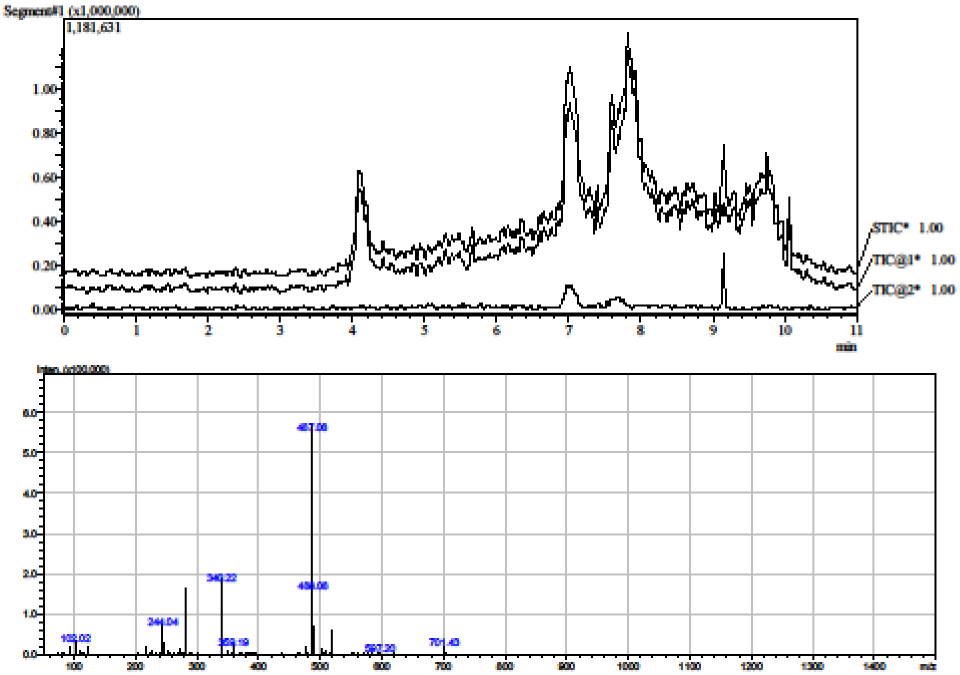

Figure S20. LCMSMS spectra of compound **3e**.

DOPNALAB

Item	Value
Acquired Date&Time	13.04.2018 15:16:03
Acquired by	System Administrator
Filename	C:\Users\dopnalab\Desktop\derya\ahmet osman tokay\GS-F1.ispd
Spectrum name	GS-F1
Sample name	GS-F
Sample ID	
Option	
Comment	
No. of Scans	10
Resolution	4 [cm-1]
Apodization	Happ-Genzel

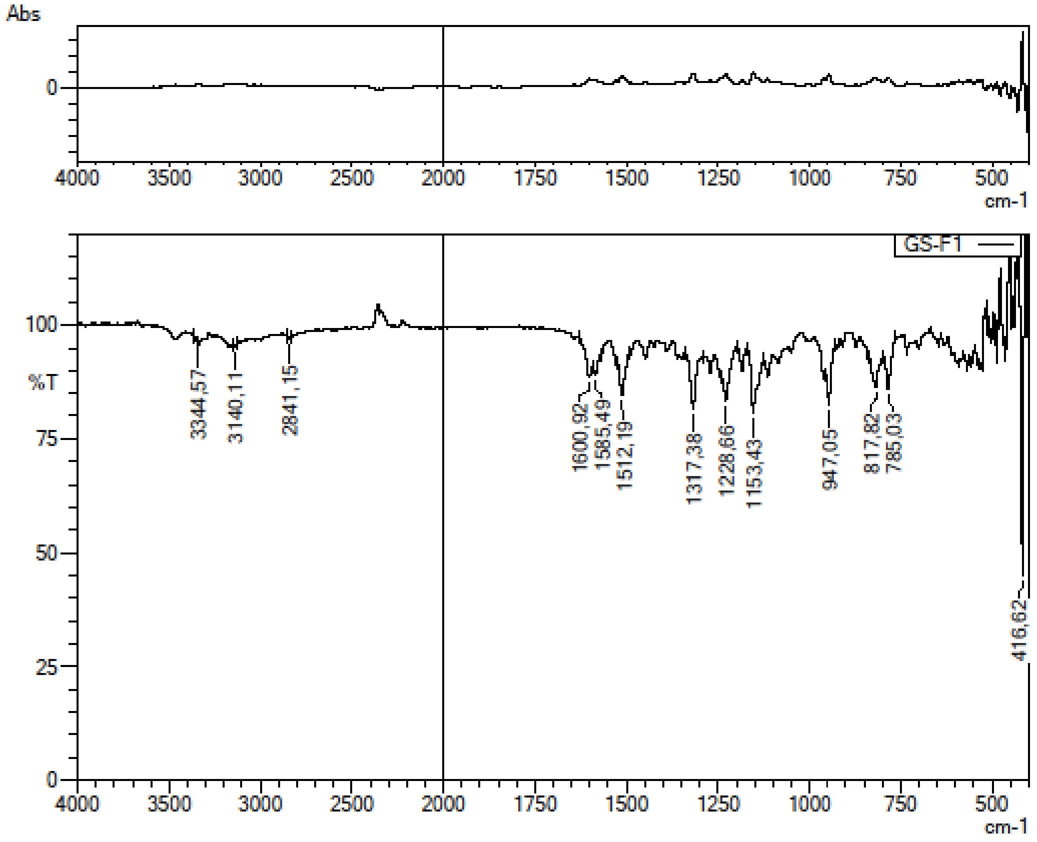

Figure S21. IR spectra of compound **3f**.

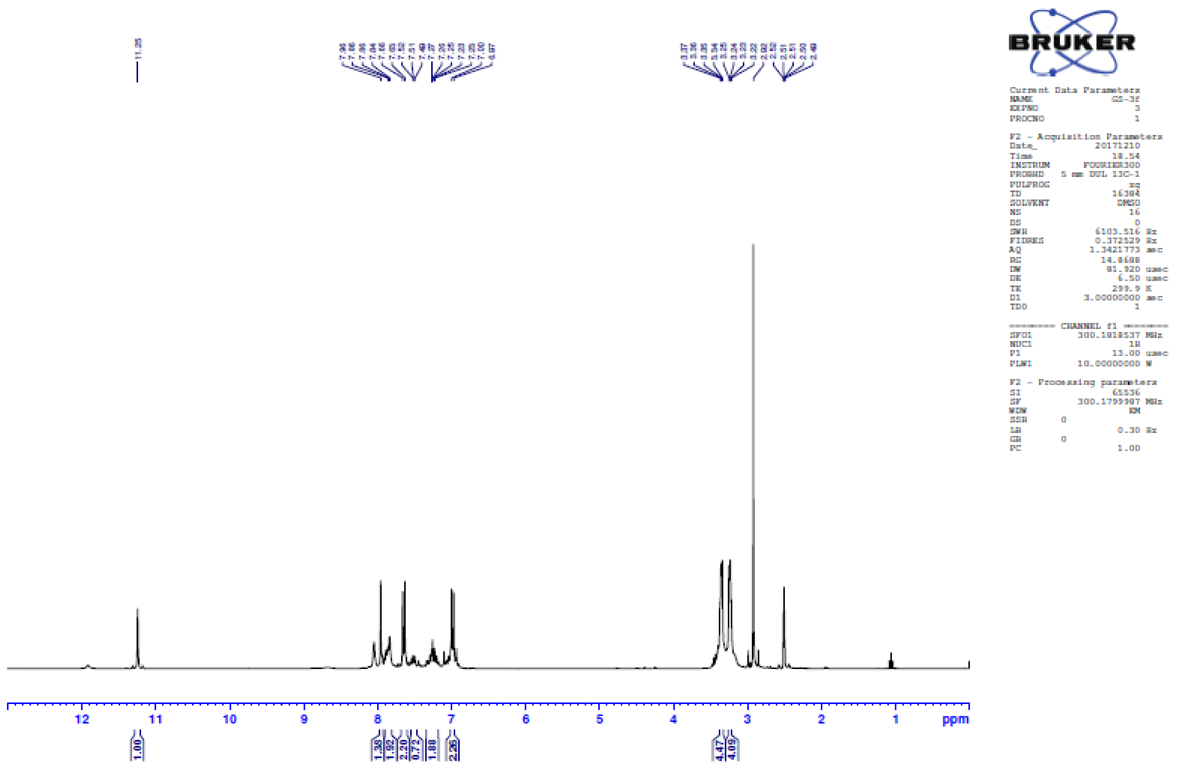

Figure S22. ^{1}H-NMR spectra of compound **3f**.

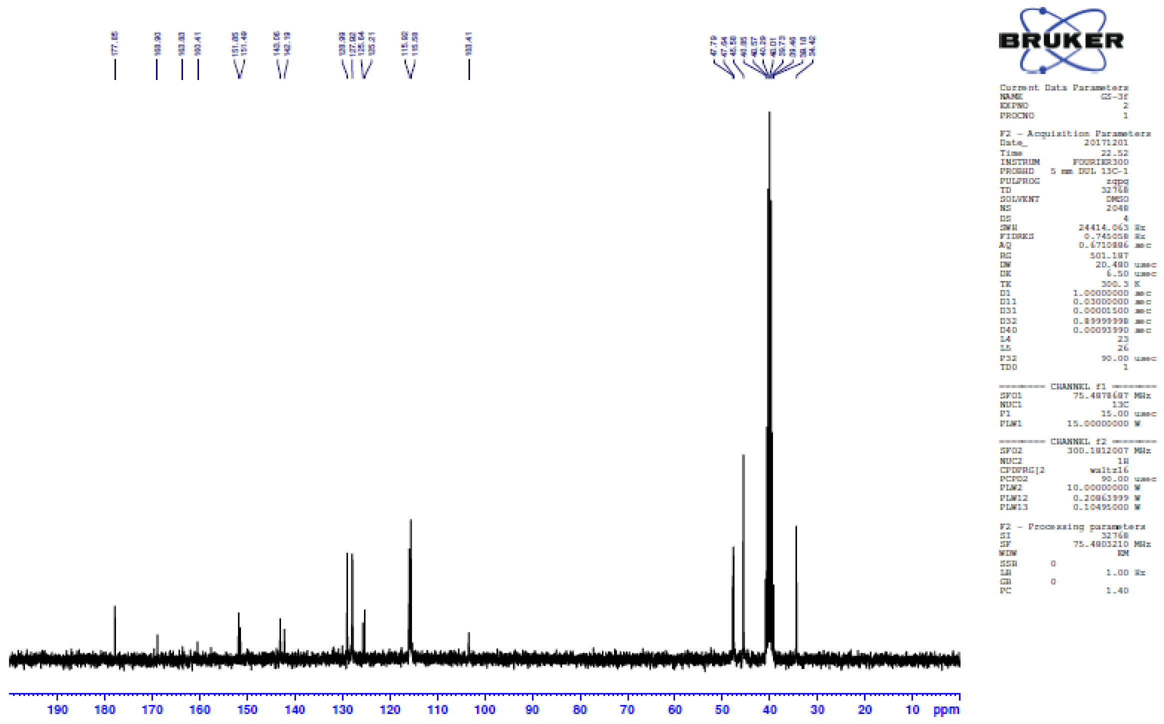

Figure S23. ^{13}C-NMR spectra of compound **3f**.

==== Shimadzu LCMSsolution Analysis Report ====

Acquired by	:Admin
Sample Name	:GS-F
Sample ID	
Vail #	:15
Injection Volume	:0.3 µL
Data File Name	:GS-F_22.lcd
Method File Name	:genel.lcm
Batch File Name	:batch.lcb
Report File Name	:DefaultLCMS.lcr
Data Acquired	:27.04.2021 10.35:10
Data Processed	:27.04.2021 10.46:14

<Chromatogram>

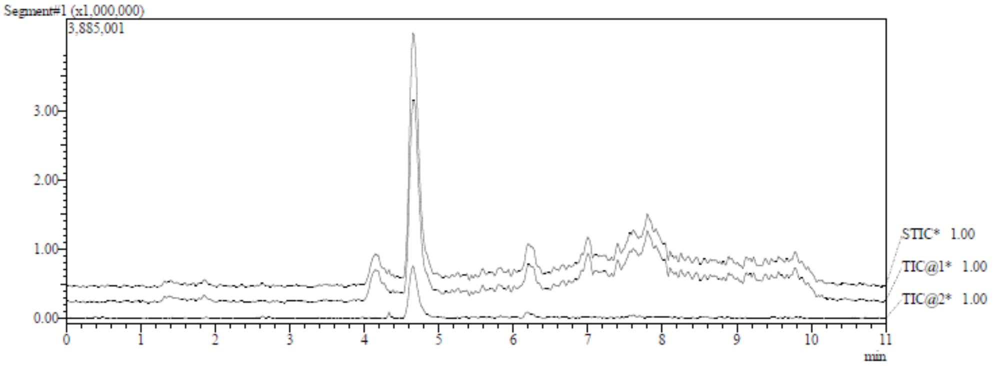

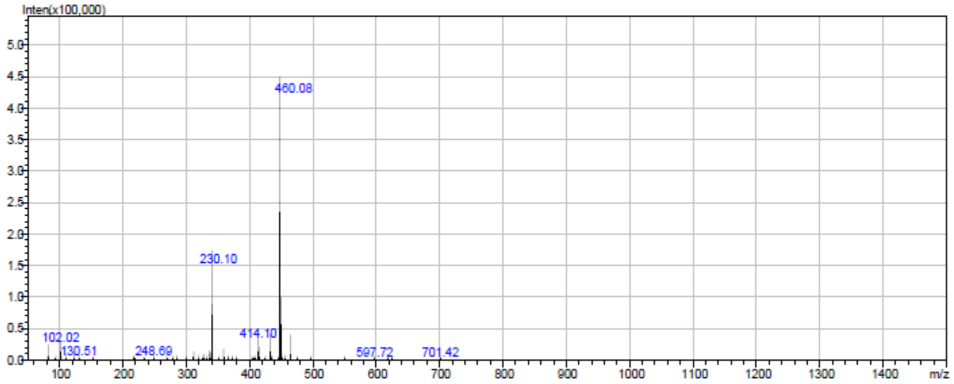

Figure S24. LCMSMS spectra of compound **3f**.

DOPNALAB

Item	Value
Acquired Date&Time	13.04.2018 15:18:29
Acquired by	System Administrator
Filename	C:\Users\dopnalab\Desktop\derya\ahmet osman tokay\GS-Cl1.lspd
Spectrum name	GS-Cl1
Sample name	GS-Cl
Sample ID	
Option	
Comment	
No. of Scans	10
Resolution	4 [cm-1]
Apodization	Happ-Genzel

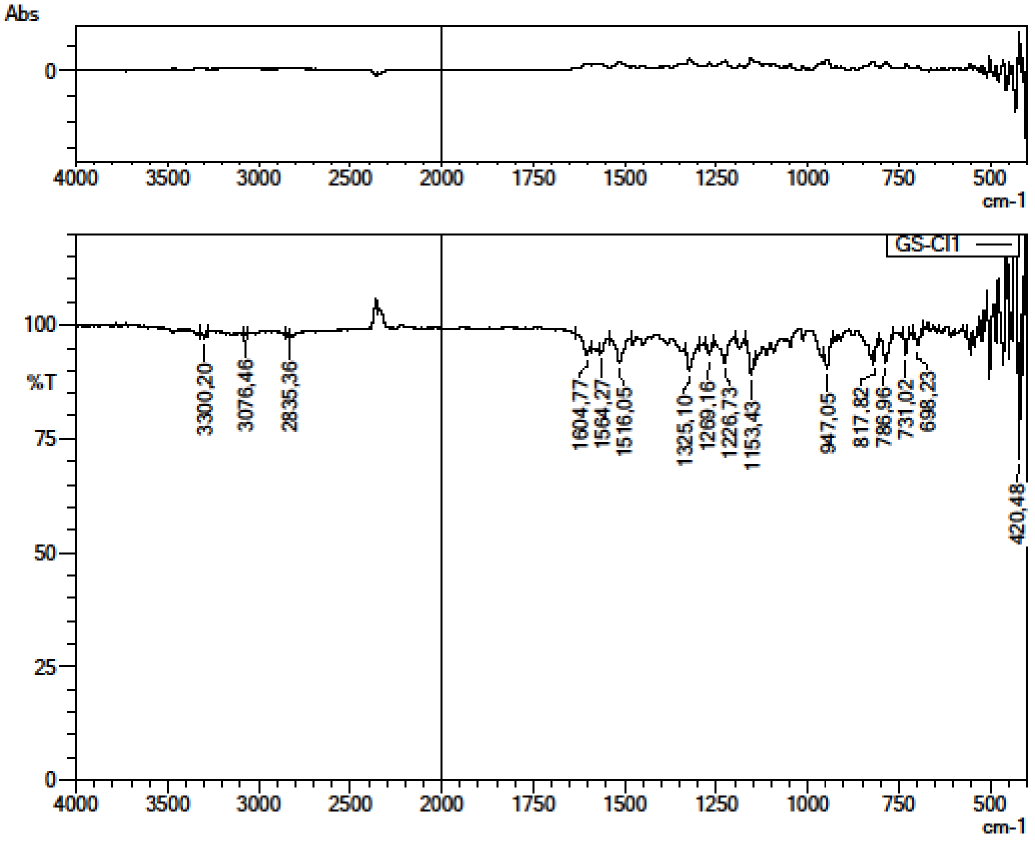

Figure S25. IR spectra of compound **3g**.

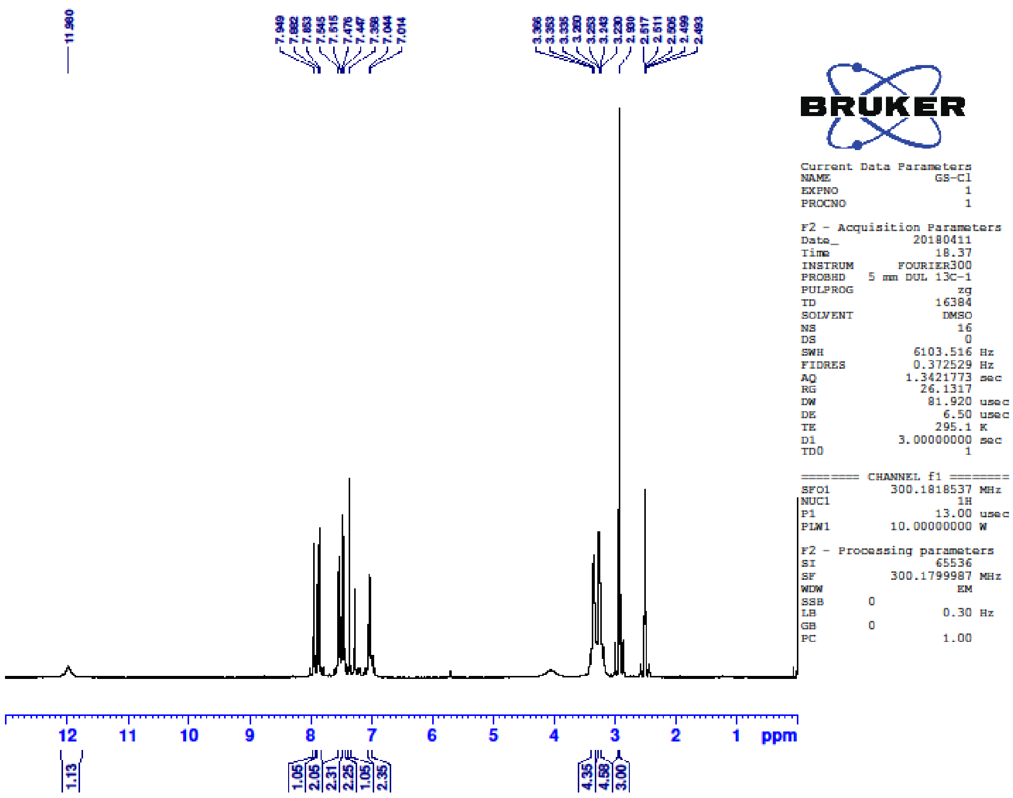

Figure S26. ^{1}H-NMR spectra of compound **3g**.

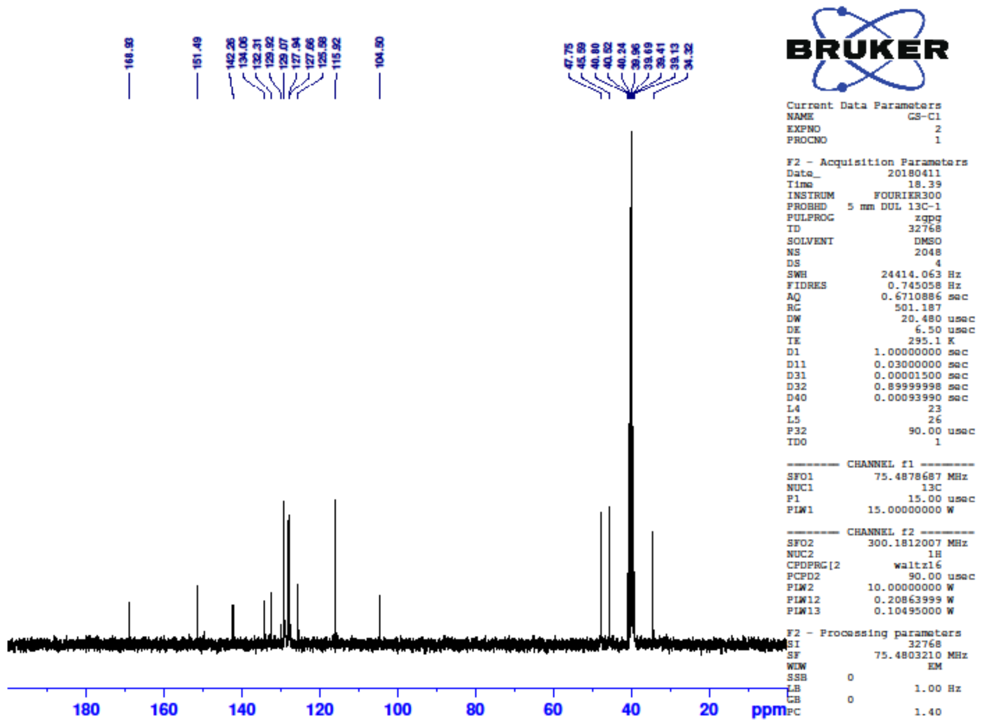

Figure S27. ^{13}C-NMR spectra of compound **3g**.

==== Shimadzu LCMSsolution Analysis Report ====

Acquired by	: Admin
Sample Name	: GS-Cl
Sample ID	:
Vail #	: 4
Injection Volume	: 0.3 uL
Data File Name	: GS-Cl_18.lcd
Method File Name	: genel.lcm
Batch File Name	: batch.lcb
Report File Name	: DefaultLCMS.lcr
Data Acquired	: 19.04.2018 12:41:02
Data Processed	: 19.04.2018 12:52:05

<Chromatogram>

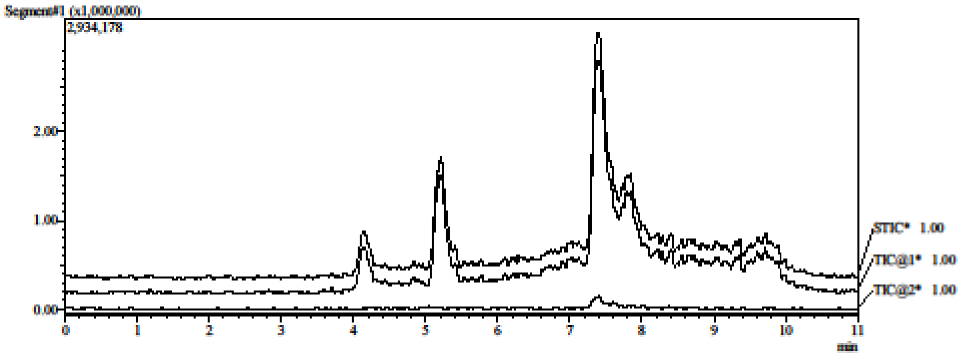

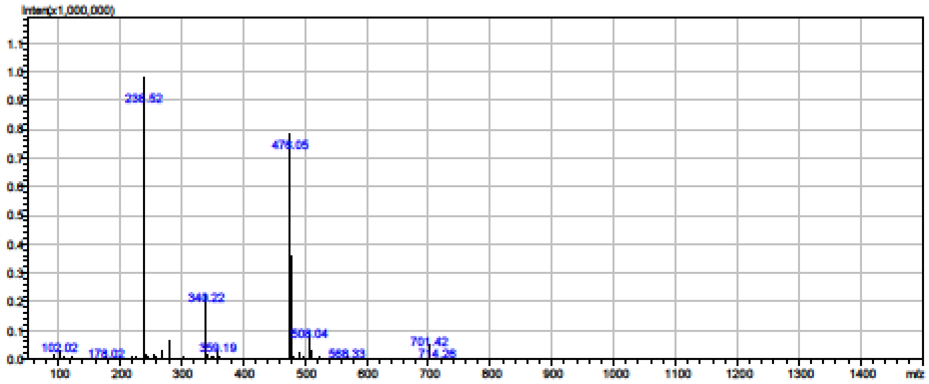

Figure S28. LCMSMS spectra of compound **3g**.

DOPNALAB

	Item	Value
	Acquired Date&Time	13.04.2018 15:21:58
	Acquired by	System Administrator
	Filename	C:\Users\dopnalab\Desktop\derya\ahmet osman tokay\GS-CF31.ispd
	Spectrum name	GS-CF31
	Sample name	GS-CF3
	Sample ID	
	Option	
	Comment	
	No. of Scans	10
	Resolution	4 [cm-1]
	Apodization	Happ-Genzel

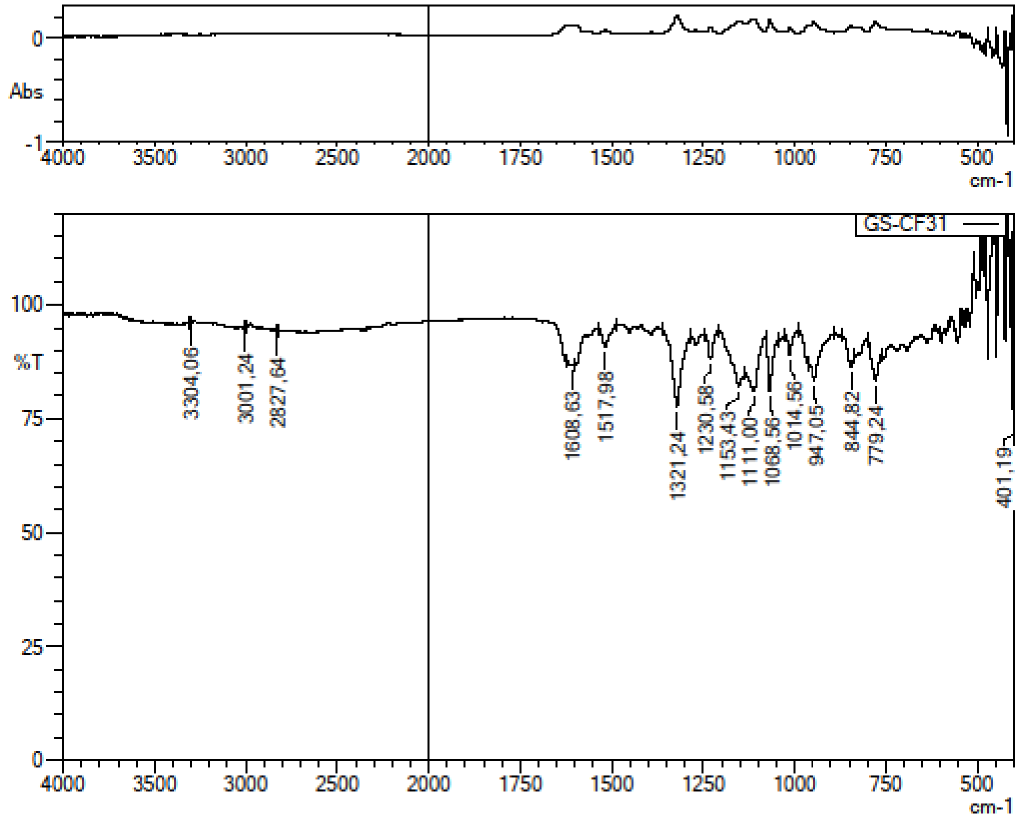

Figure S29. IR spectra of compound **3h**.

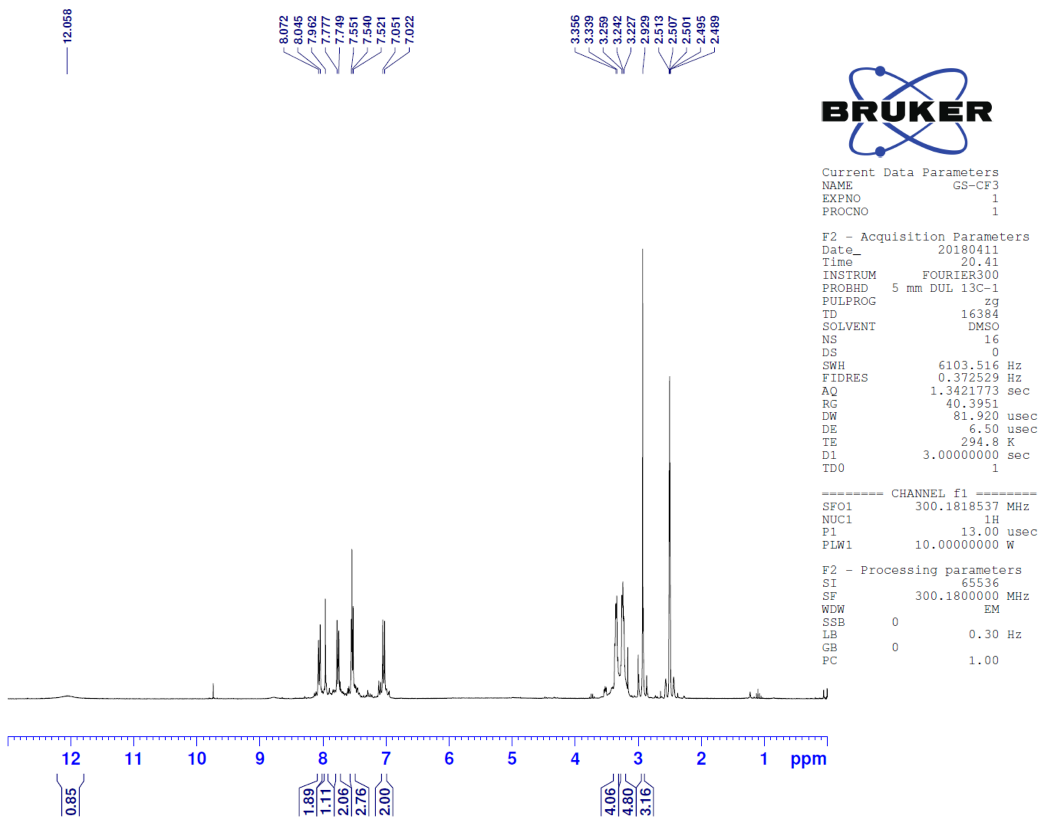

Figure S30. ^{1}H-NMR spectra of compound **3h**.

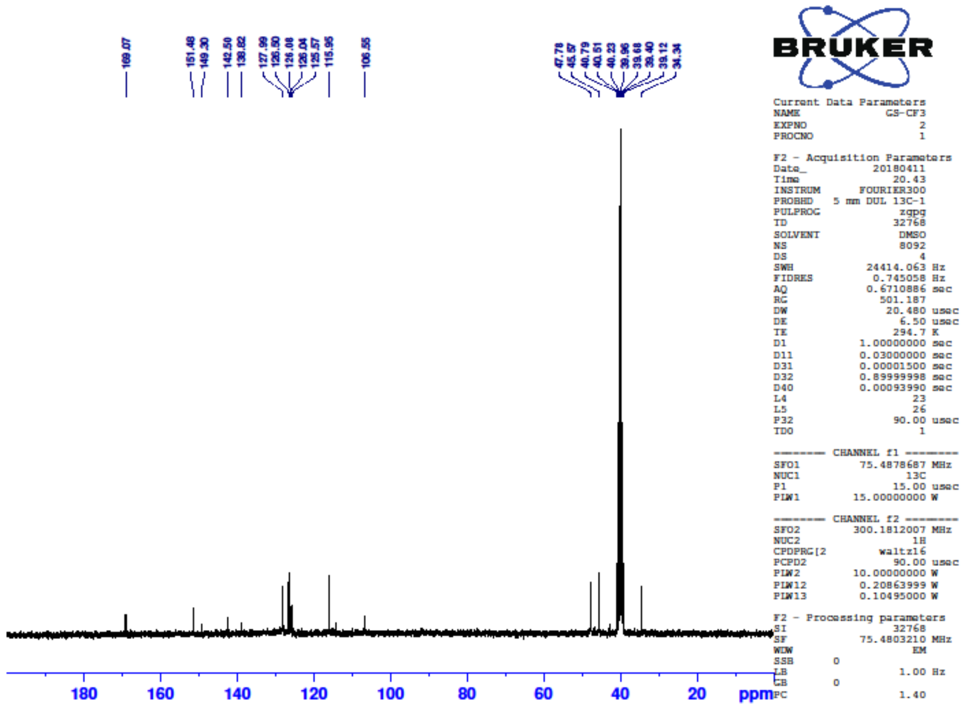

Figure S31. ^{13}C-NMR spectra of compound **3h**.

==== Shimadzu LCMSsolution Analysis Report ====

Acquired by	: Admin
Sample Name	: GS-CF3
Sample ID	:
Vail #	: 3
Injection Volume	: 0.3 uL
Data File Name	: GS-CF3_17.lcd
Method File Name	: genel.lcm
Batch File Name	: batch.lcb
Report File Name	: DefaultLCMS.lcr
Data Acquired	: 19.04.2018 12:29:29
Data Processed	: 19.04.2018 12:40:32

<Chromatogram>

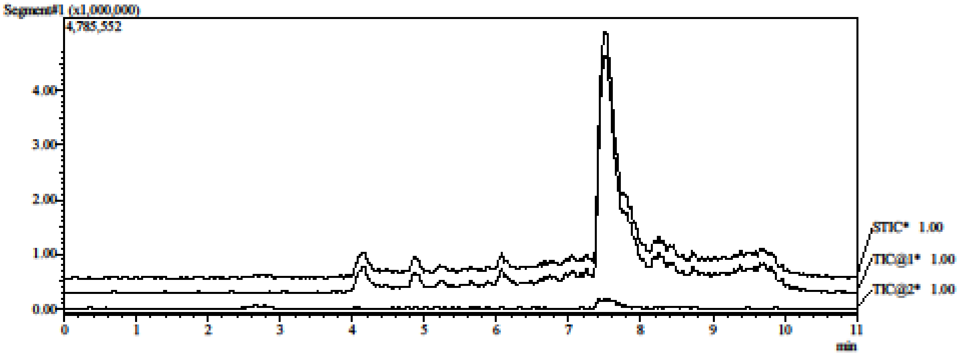

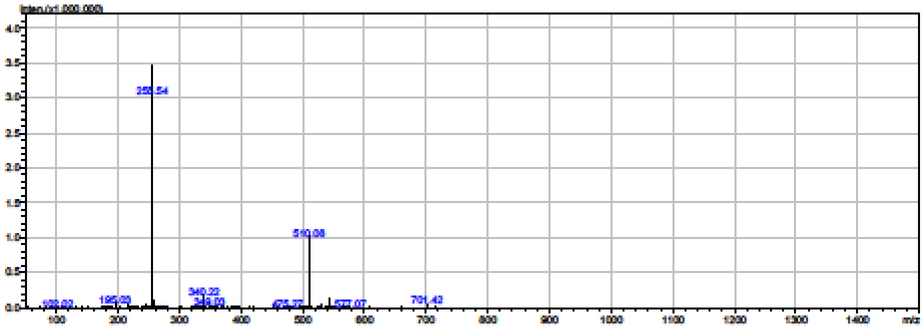

Figure S32. LCMSMS spectra of compound **3h**.

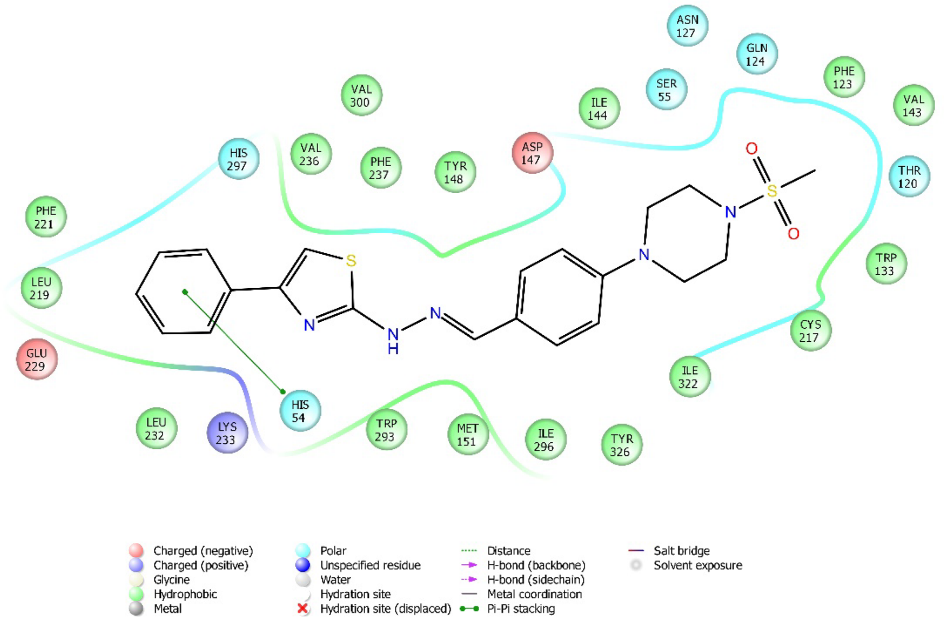

Figure S33. The two-dimensional interacting mode of compound **3a** in the active region of μ-opioid receptor (PDB Code: 5C1M).

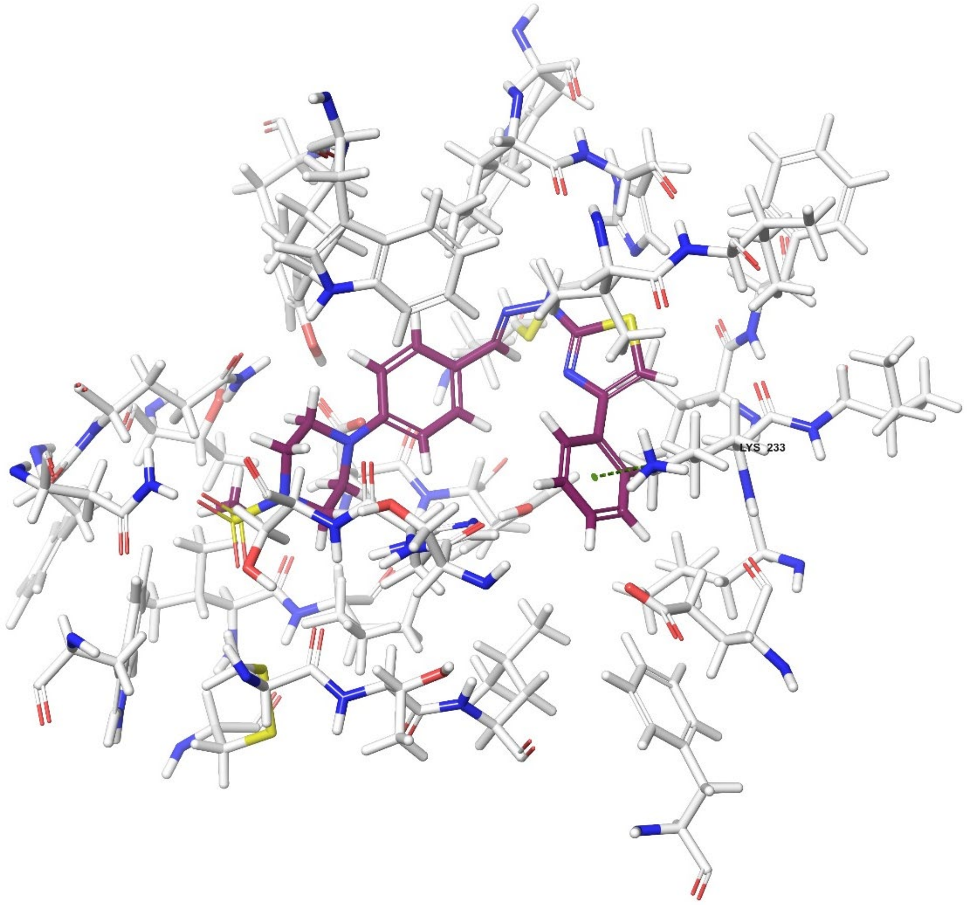

Figure S34. The three-dimensional interacting mode of compound **3a** in the active region of μ-opioid receptor. The ligand and significant residues of the active site of the receptor are presented by a tube model colored with purple and white, respectively (PDB Code: 5C1M).

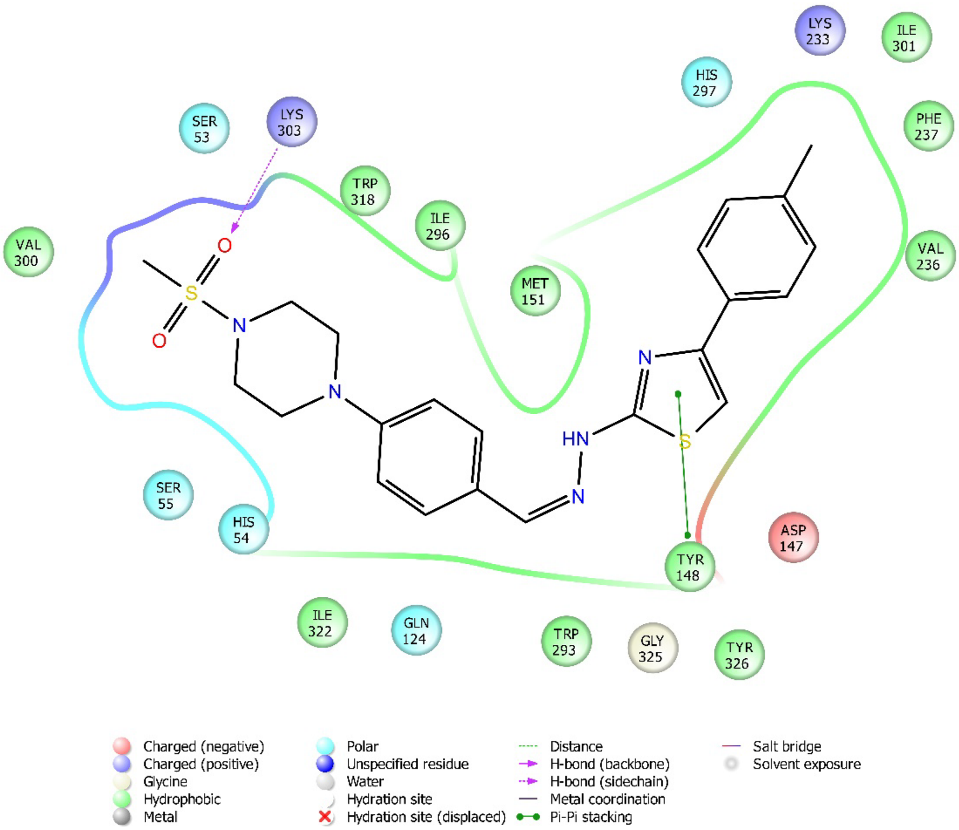

Figure S35. The two-dimensional interacting mode of compound **3b** in the active region of μ-opioid receptor (PDB Code: 5C1M).

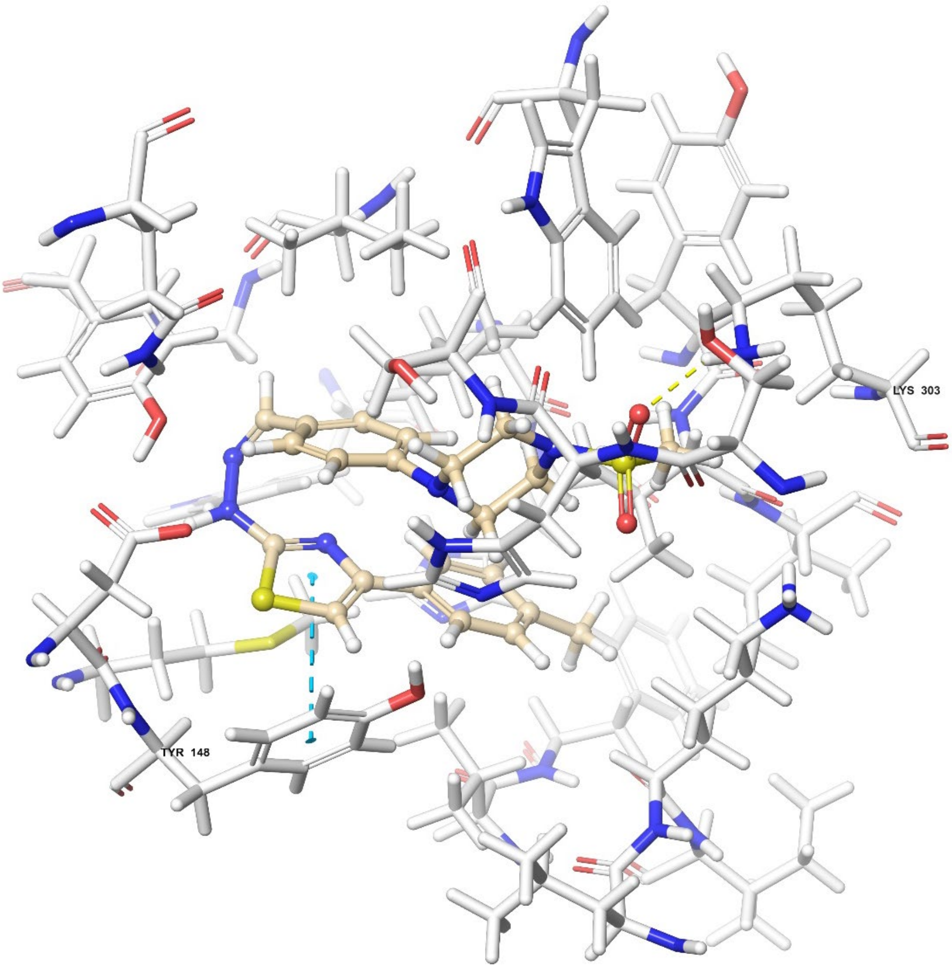

Figure S36. The three-dimensional interacting mode of compound **3b** in the active region of μ-opioid receptor. The ligand and significant residues of the active site of the receptor are presented by a tube model colored with orange and white, respectively (PDB Code: 5C1M).

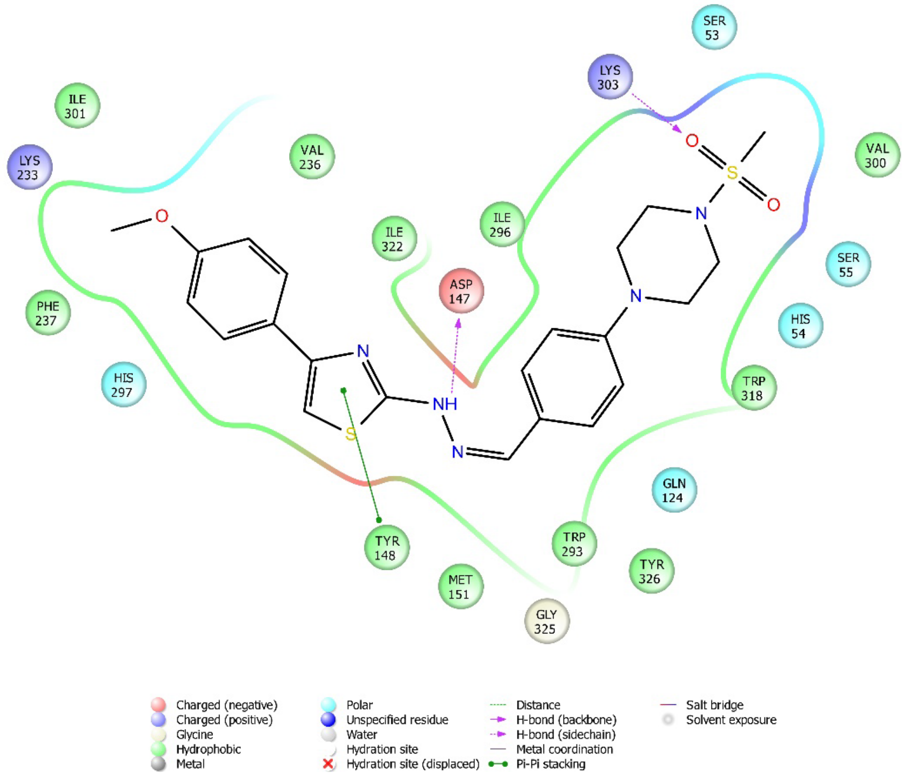

Figure S37. The two-dimensional interacting mode of compound **3c** in the active region of μ-opioid receptor (PDB Code: 5C1M).

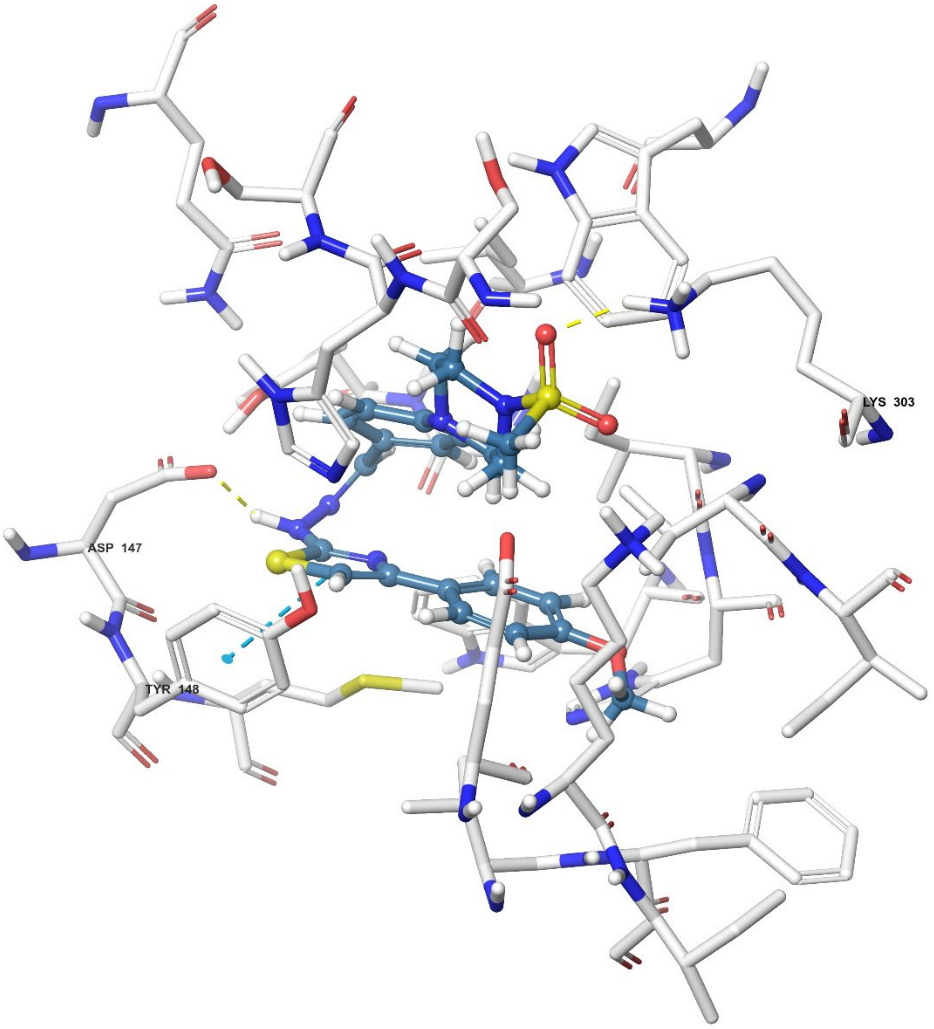

Figure S38. The three-dimensional interacting mode of compound **3c** in the active region of μ-opioid receptor. The ligand and significant residues of the active site of the receptor are presented by a tube model colored with blue and white, respectively (PDB Code: 5C1M).

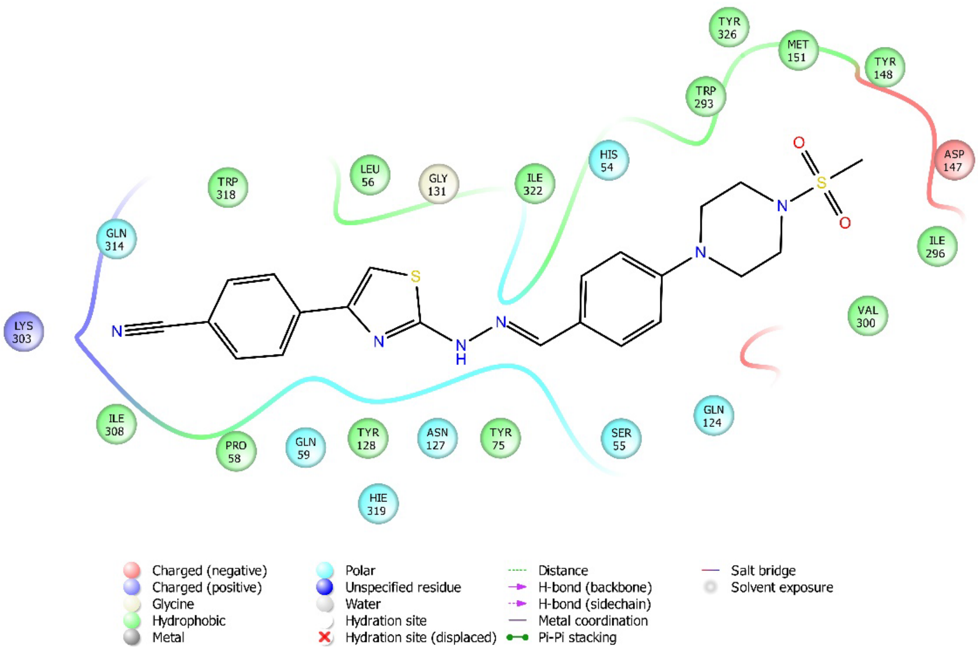

Figure S39. The two-dimensional interacting mode of compound **3d** in the active region of μ-opioid receptor (PDB Code: 5C1M).

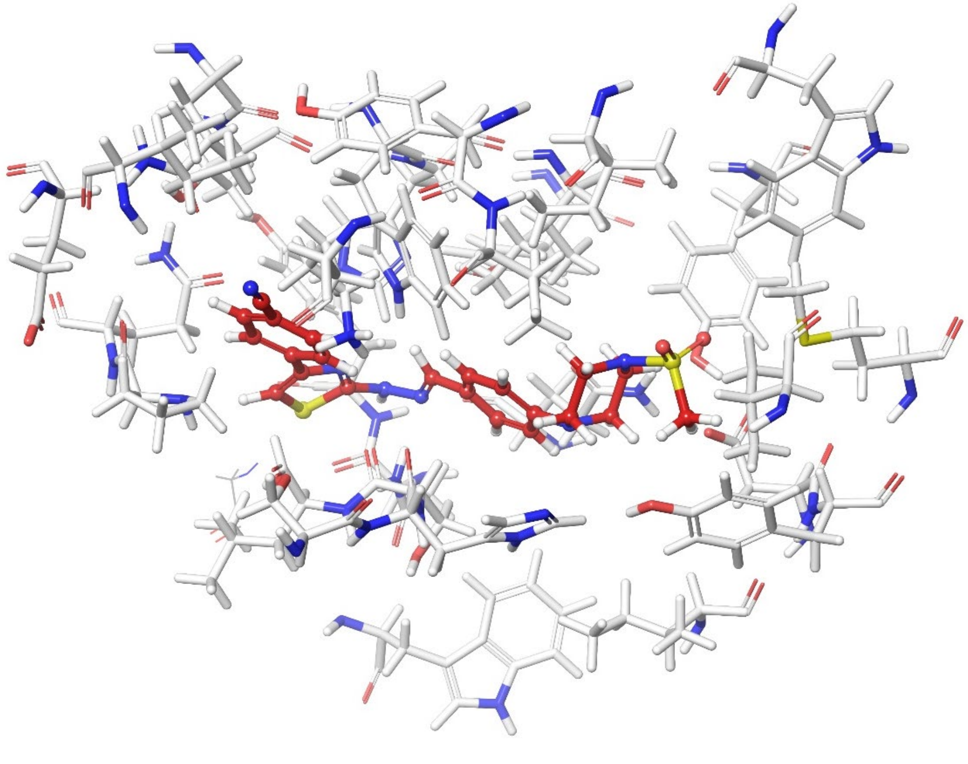

Figure S40. The three-dimensional interacting mode of compound **3d** in the active region of μ-opioid receptor. The ligand and significant residues of the active site of the receptor are presented by a tube model colored with red and white, respectively (PDB Code: 5C1M).

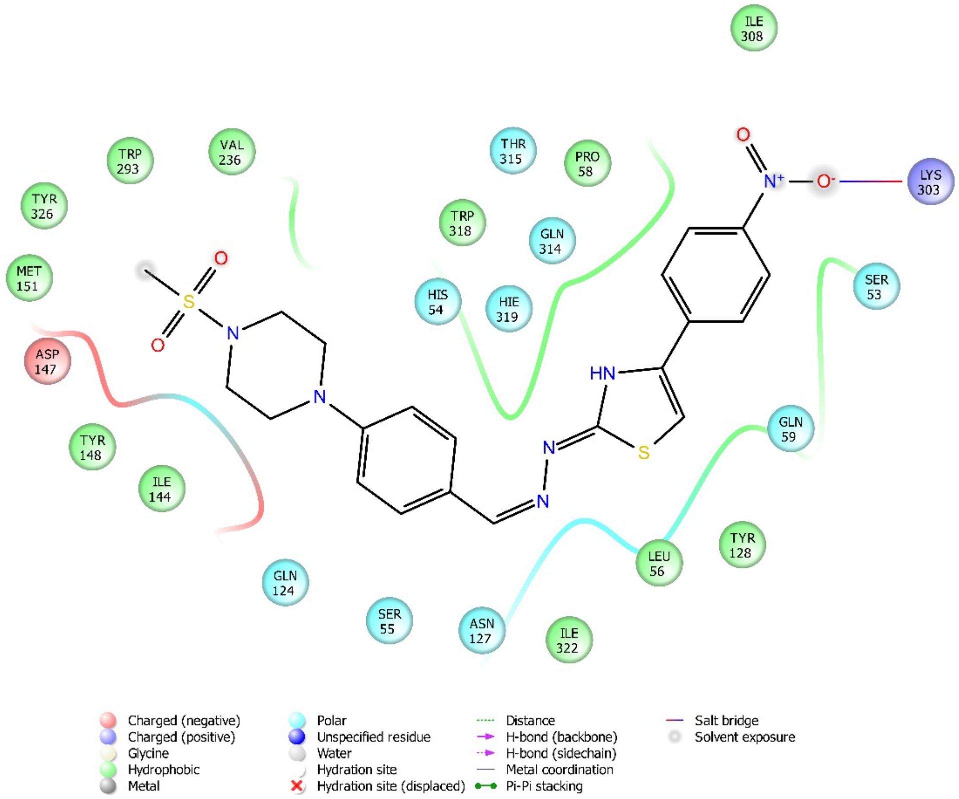

Figure S41. The two-dimensional interacting mode of compound **3e** in the active region of μ-opioid receptor (PDB Code: 5C1M).

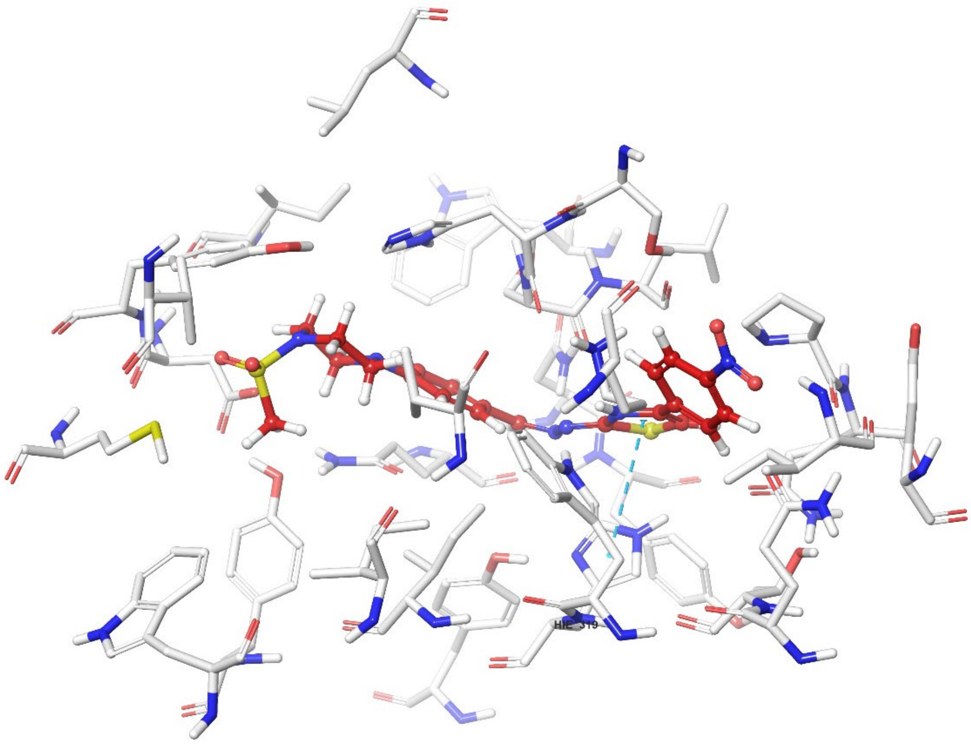

Figure S42. The three-dimensional interacting mode of compound **3e** in the active region of μ-opioid receptor. The ligand and significant residues of the active site of the receptor are presented by a tube model colored with red and white, respectively (PDB Code: 5C1M).

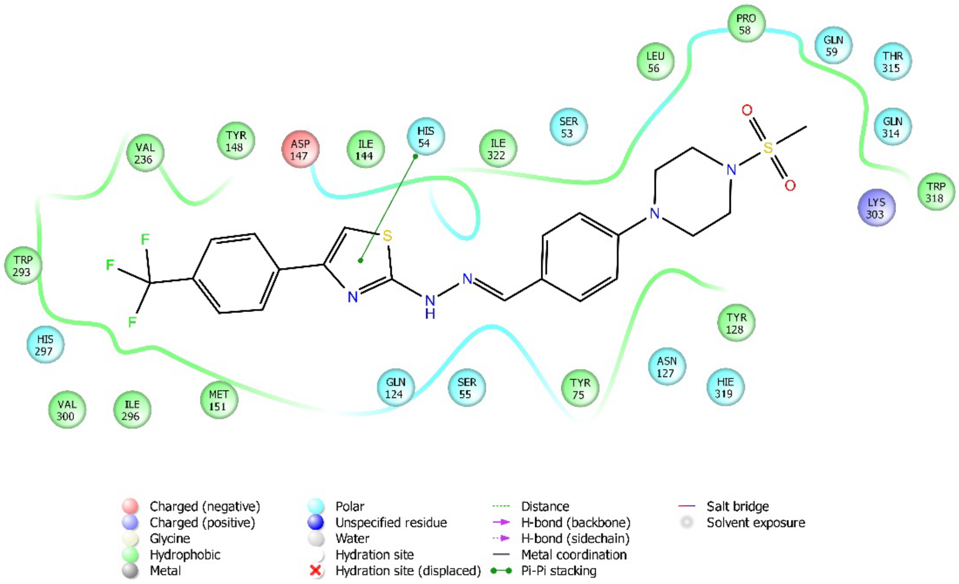

Figure S43. The two-dimensional interacting mode of compound **3h** in the active region of μ-opioid receptor (PDB Code: 5C1M).

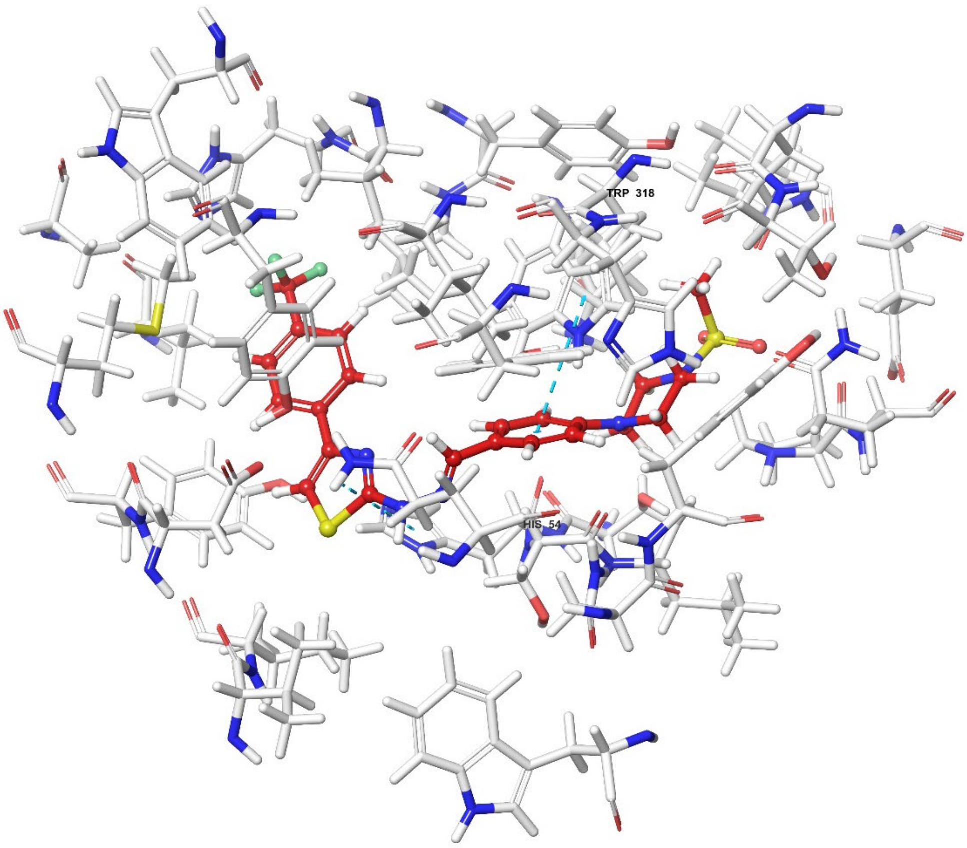

Figure S44. The three-dimensional interacting mode of compound **3h** in the active region of μ-opioid receptor. The ligand and significant residues of the active site of the receptor are presented by a tube model colored with red and white, respectively (PDB Code: 5C1M).

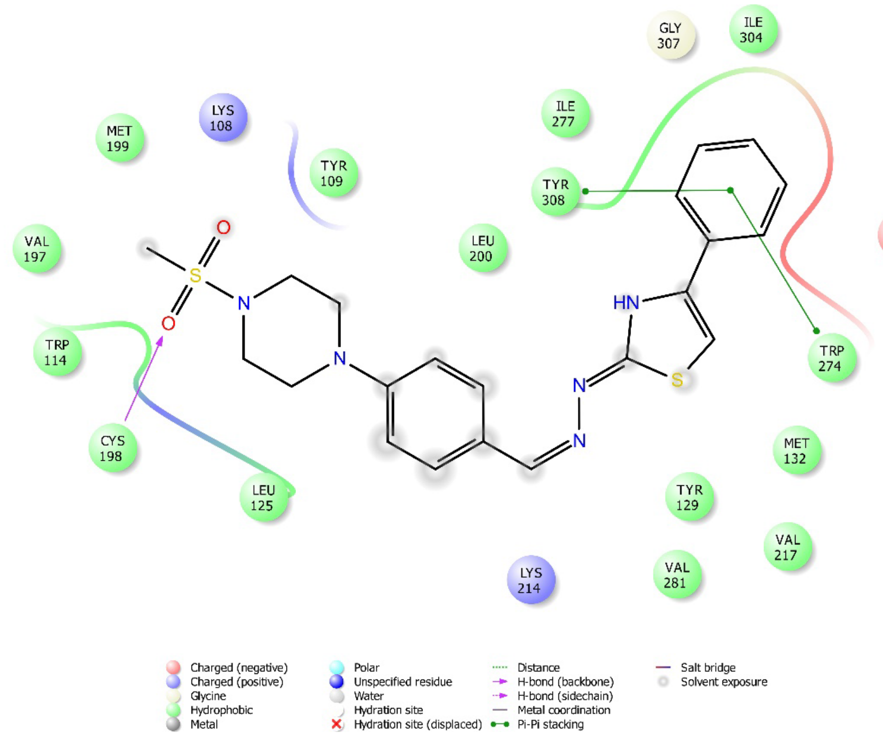

Figure S45. The two-dimensional interacting mode of compound **3a** in the active region of δ-opioid receptor (PDB Code: 4N6H).

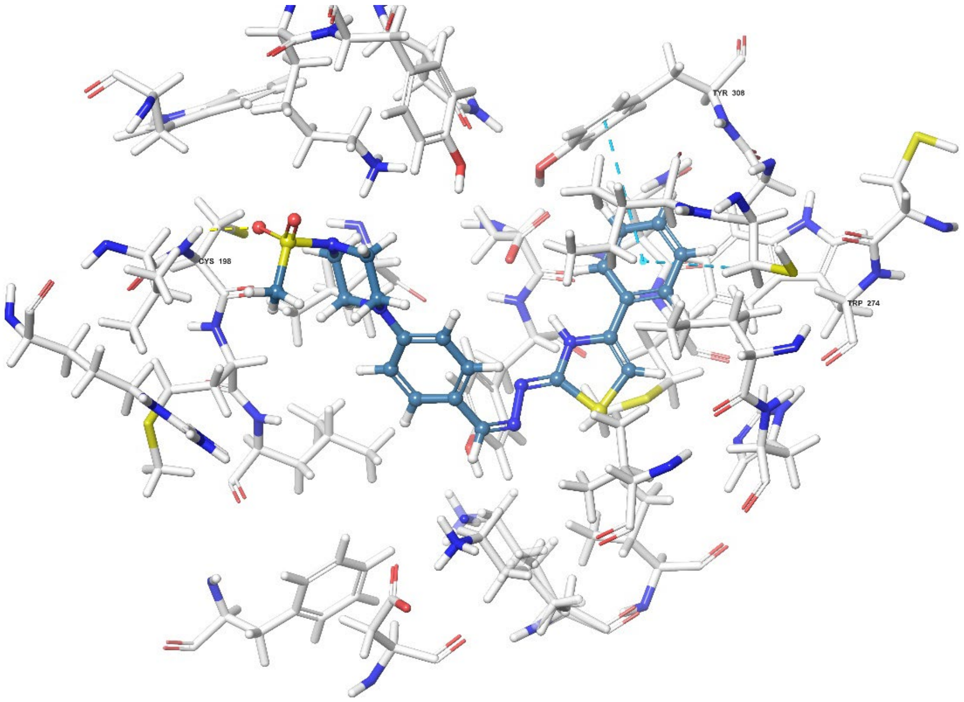

Figure S46. The three-dimensional interacting mode of compound **3a** in the active region of δ-opioid receptor. The ligand and significant residues of the active site of the receptor are presented by a tube model colored with blue and white, respectively (PDB Code: 4N6H).

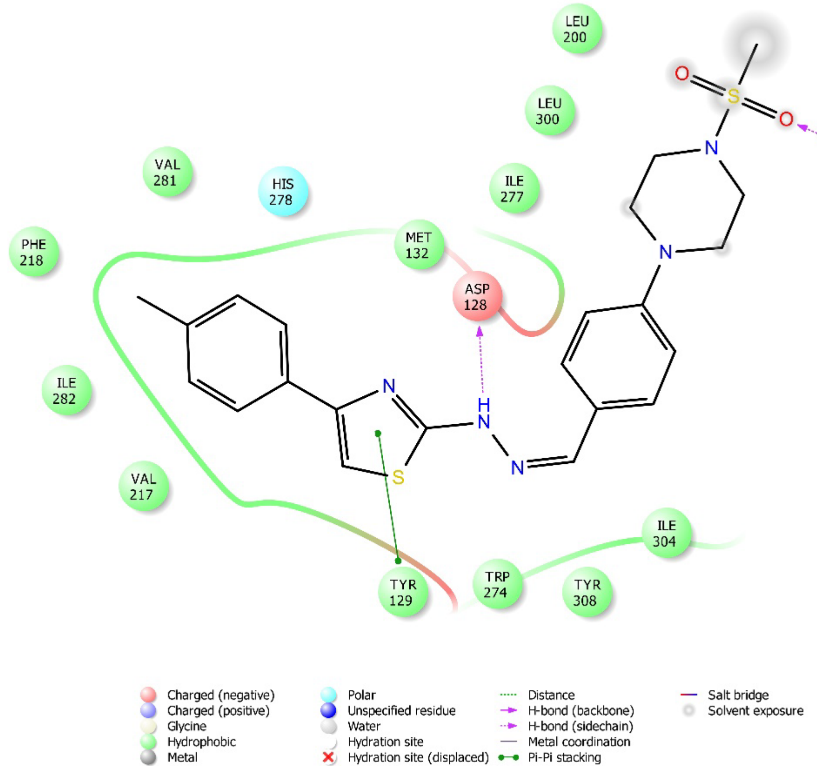

Figure S47. The two-dimensional interacting mode of compound **3b** in the active region of δ-opioid receptor (PDB Code: 4N6H).

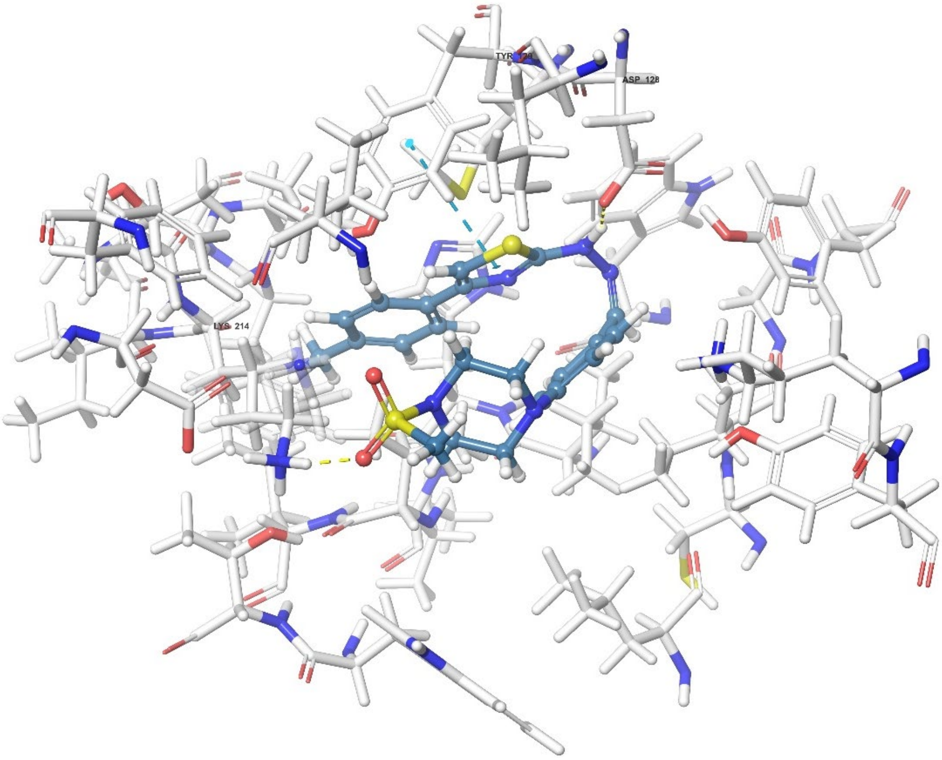

Figure S48. The three-dimensional interacting mode of compound **3b** in the active region of δ-opioid receptor. The ligand and significant residues of the active site of the receptor are presented by a tube model colored with blue and white, respectively (PDB Code: 4N6H).

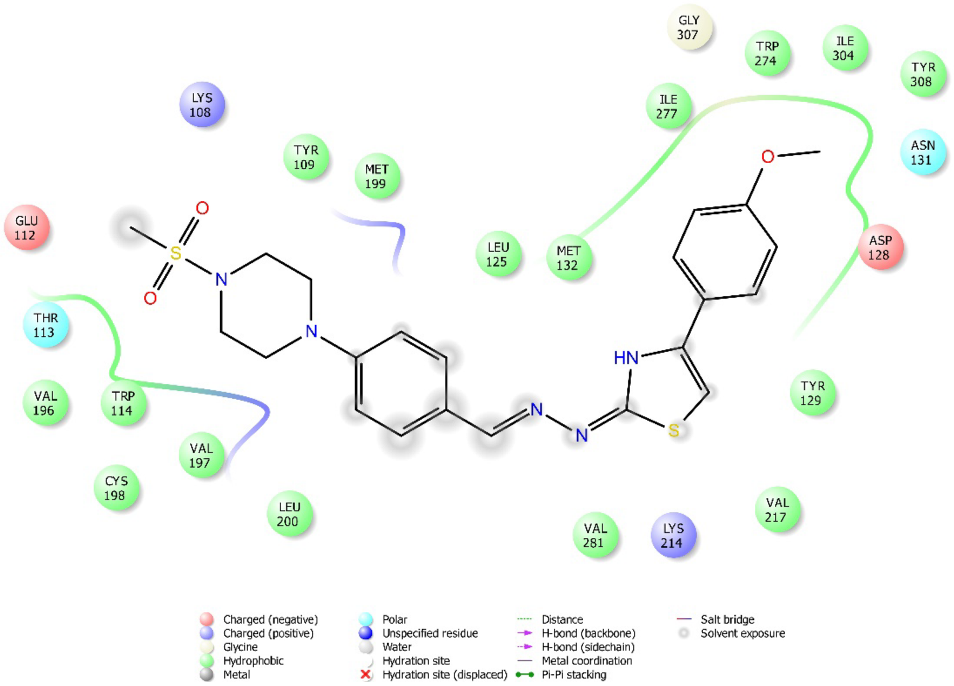

Figure S49. The two-dimensional interacting mode of compound **3c** in the active region of δ-opioid receptor (PDB Code: 4N6H).

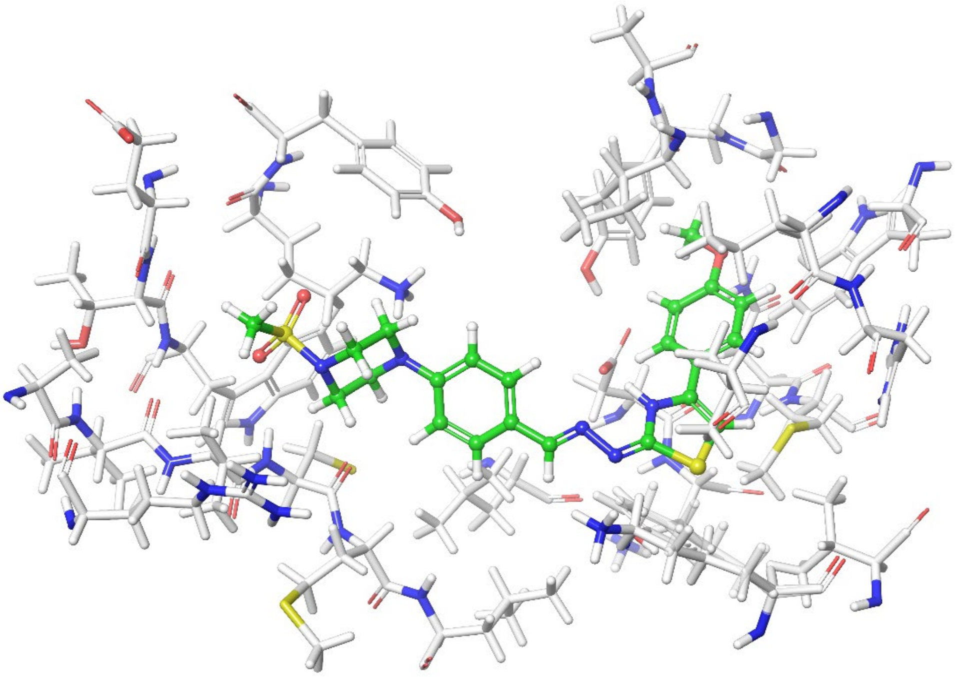

Figure S50. The three-dimensional interacting mode of compound **3c** in the active region of δ-opioid receptor. The ligand and significant residues of the active site of the receptor are presented by a tube model colored with green and white, respectively (PDB Code: 4N6H).

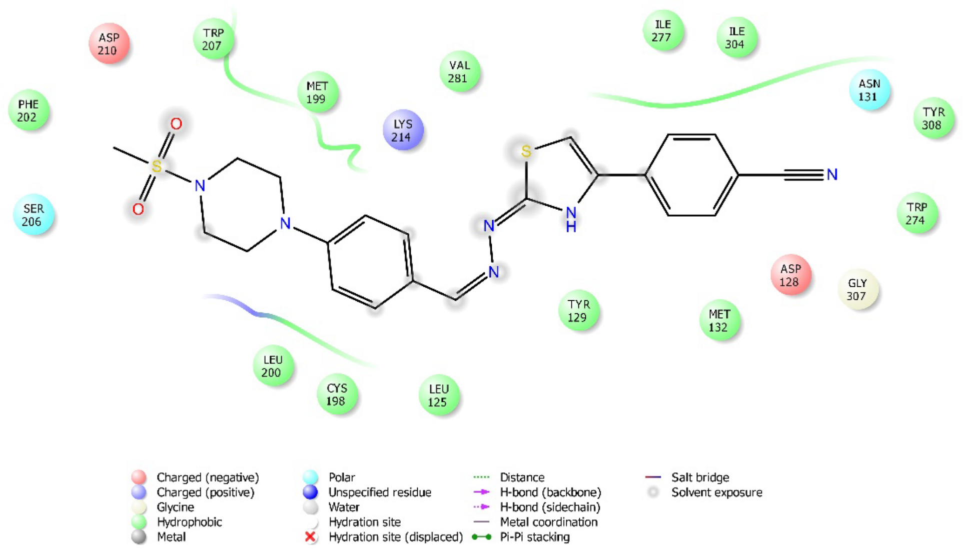

Figure S51. The two-dimensional interacting mode of compound **3d** in the active region of δ-opioid receptor (PDB Code: 4N6H).

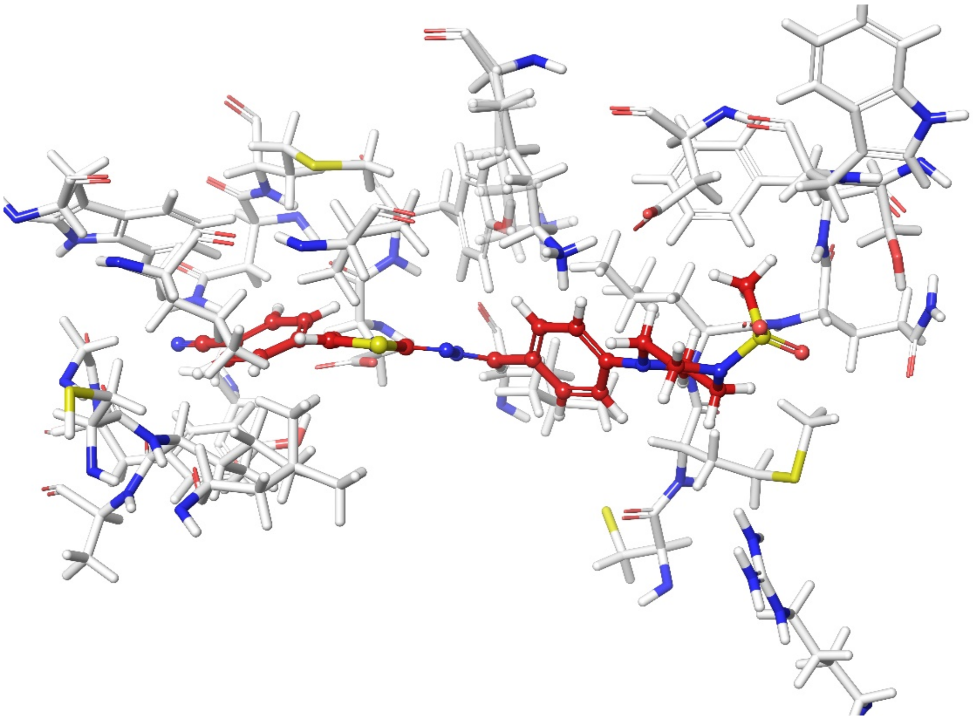

Figure S52. The three-dimensional interacting mode of compound **3d** in the active region of δ-opioid receptor. The ligand and significant residues of the active site of the receptor are presented by a tube model colored with red and white, respectively (PDB Code: 4N6H).

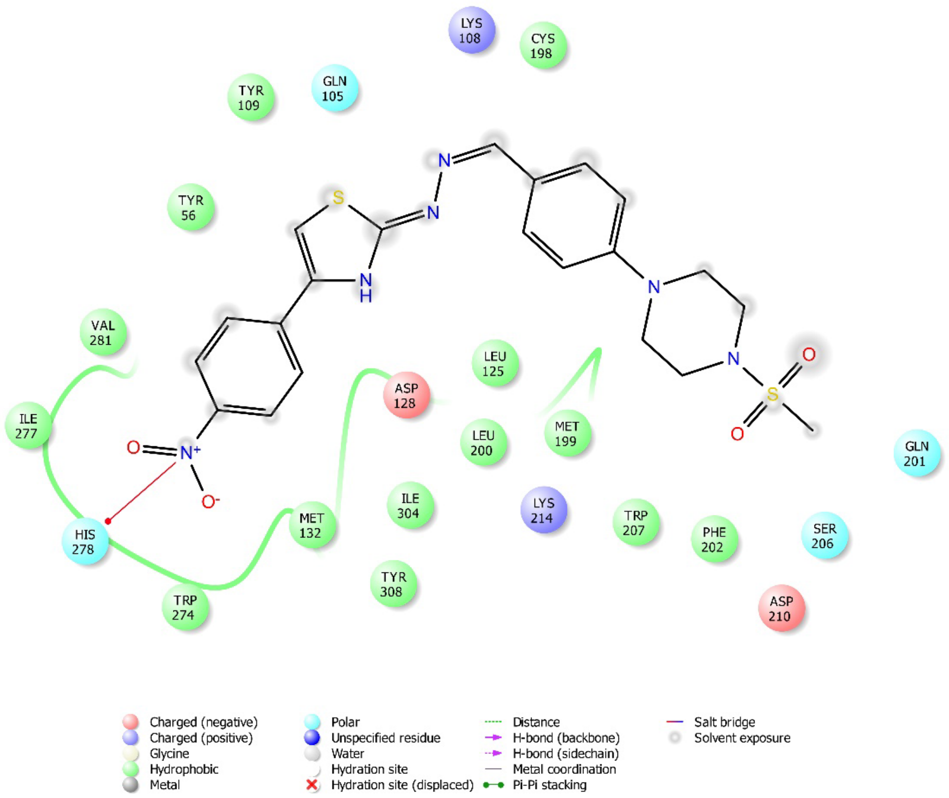

Figure S53. The two-dimensional interacting mode of compound **3e** in the active region of δ-opioid receptor (PDB Code: 4N6H).

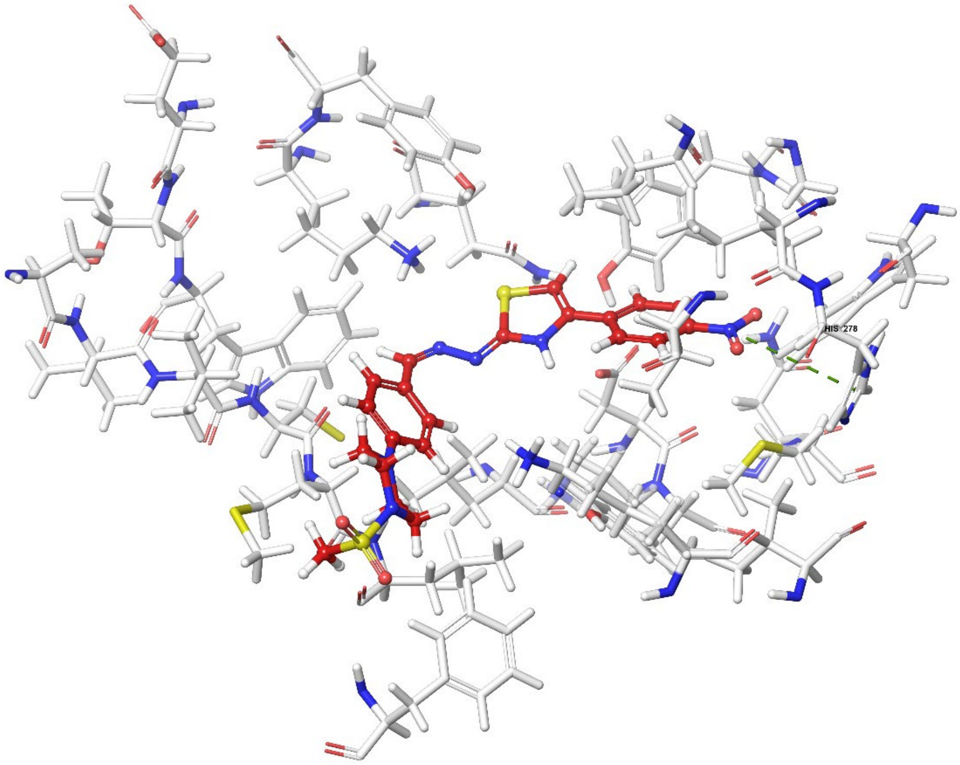

Figure S54. The three-dimensional interacting mode of compound **3e** in the active region of δ-opioid receptor. The ligand and significant residues of the active site of the receptor are presented by a tube model colored with red and white, respectively (PDB Code: 4N6H).

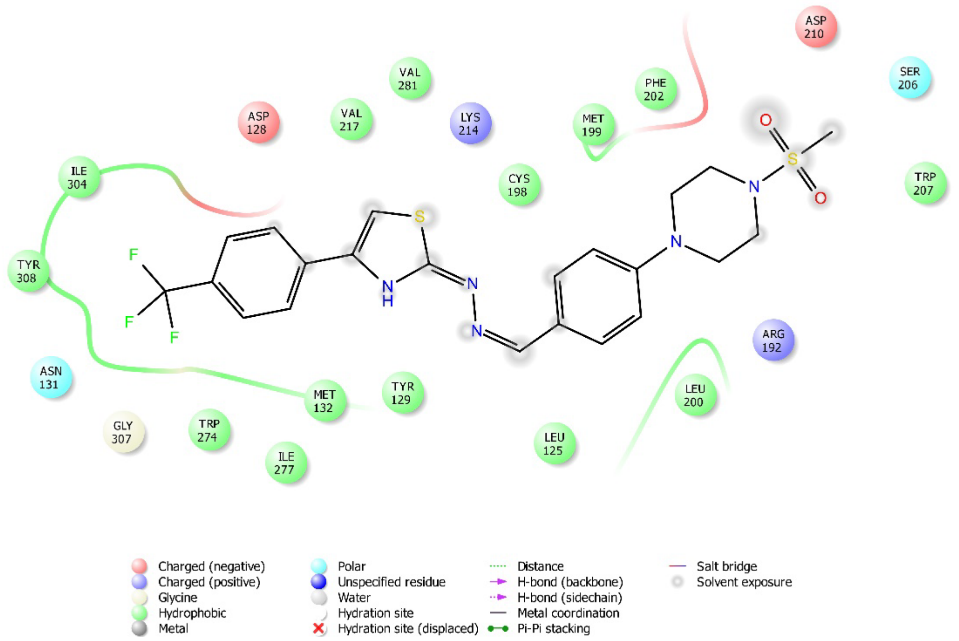

Figure S55. The two-dimensional interacting mode of compound **3h** in the active region of δ-opioid receptor (PDB Code: 4N6H).

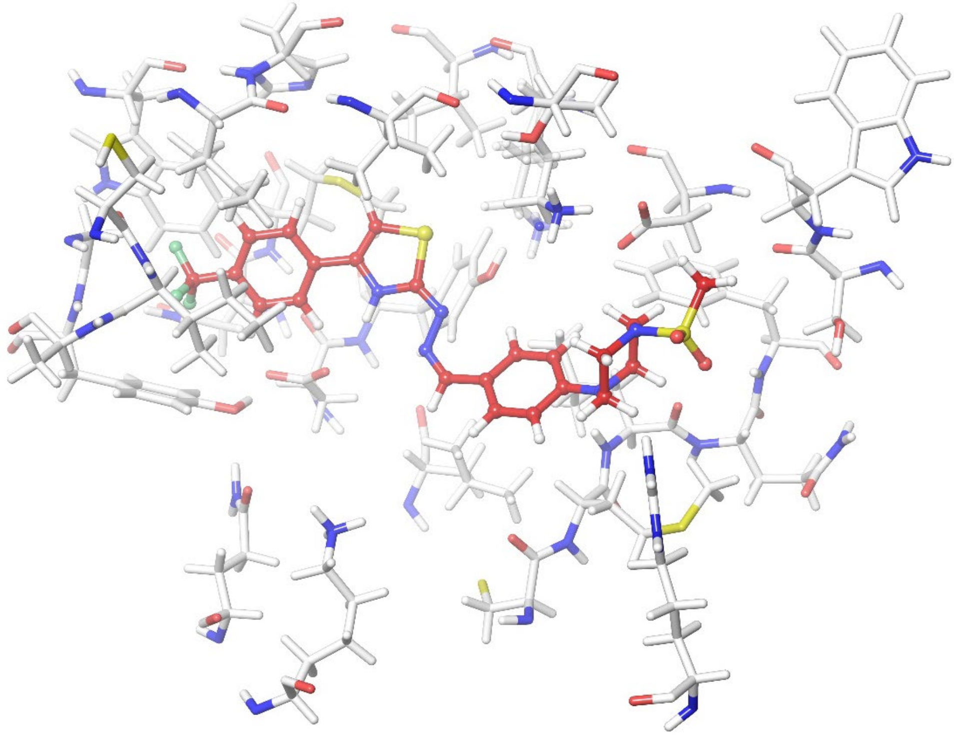

Figure S56. The three-dimensional interacting mode of compound **3h** in the active region of δ-opioid receptor. The ligand and significant residues of the active site of the receptor are presented by a tube model colored with red and white, respectively (PDB Code: 4N6H).

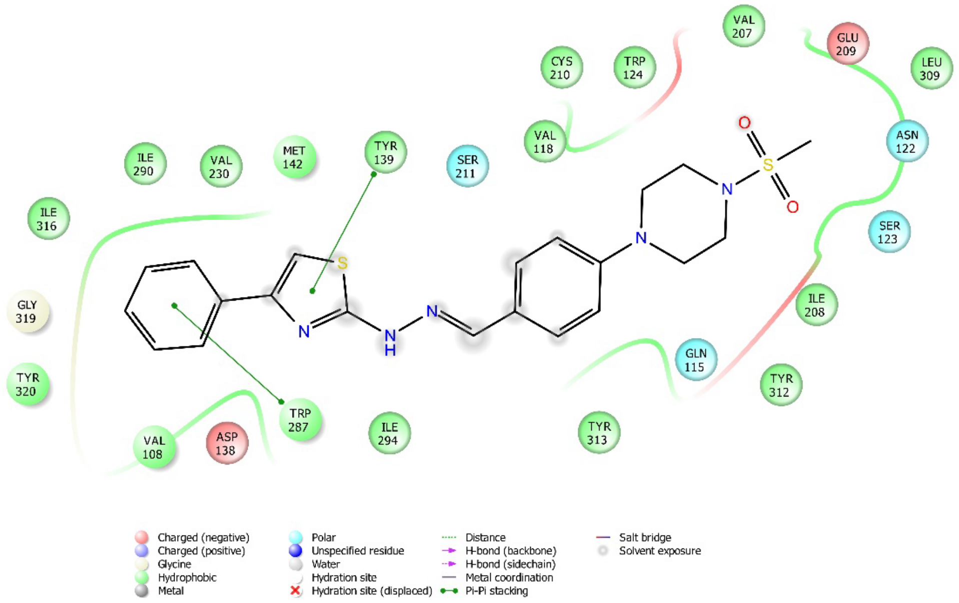

Figure S57. The two-dimensional interacting mode of compound **3a** in the active region of κ-opioid receptor (PDB Code: 6B73).

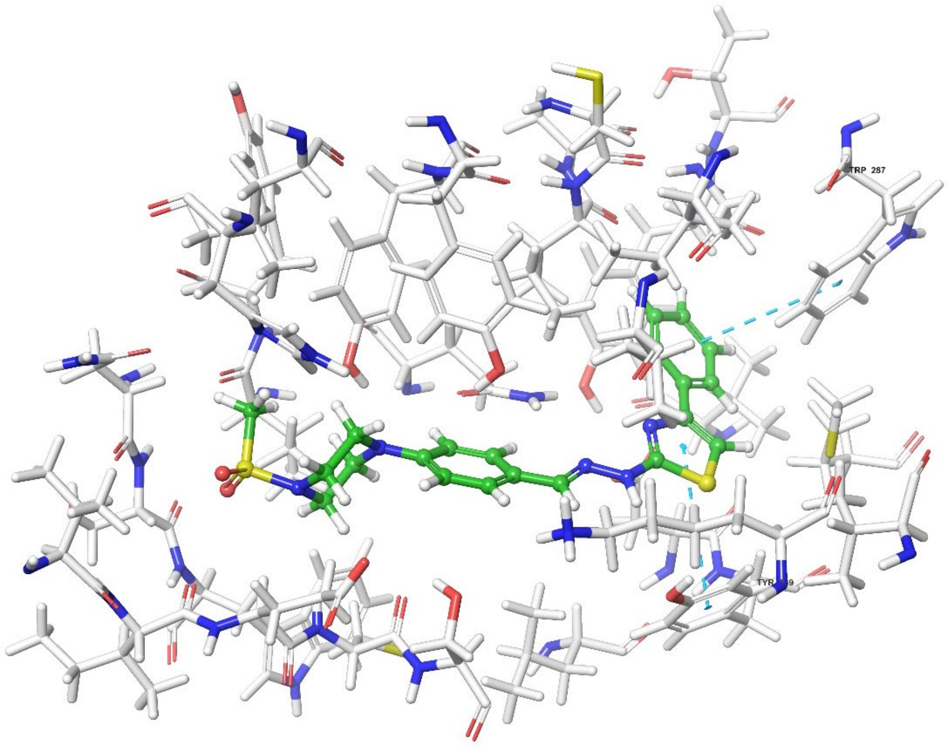

Figure S58. The three-dimensional interacting mode of compound **3a** in the active region of κ-opioid receptor. The ligand and significant residues of the active site of the receptor are presented by a tube model colored with green and white, respectively (PDB Code: 6B73).

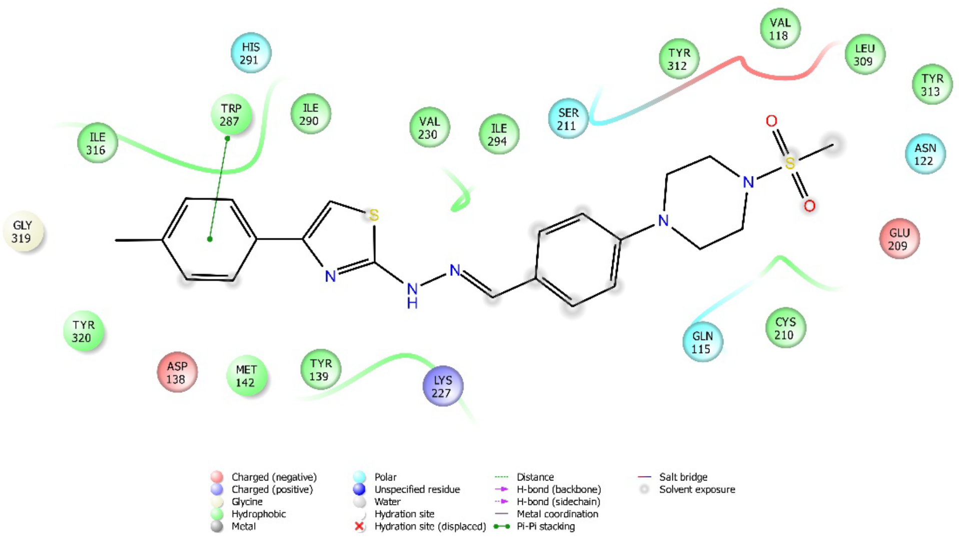

Figure S59. The two-dimensional interacting mode of compound **3b** in the active region of κ-opioid receptor (PDB Code: 6B73).

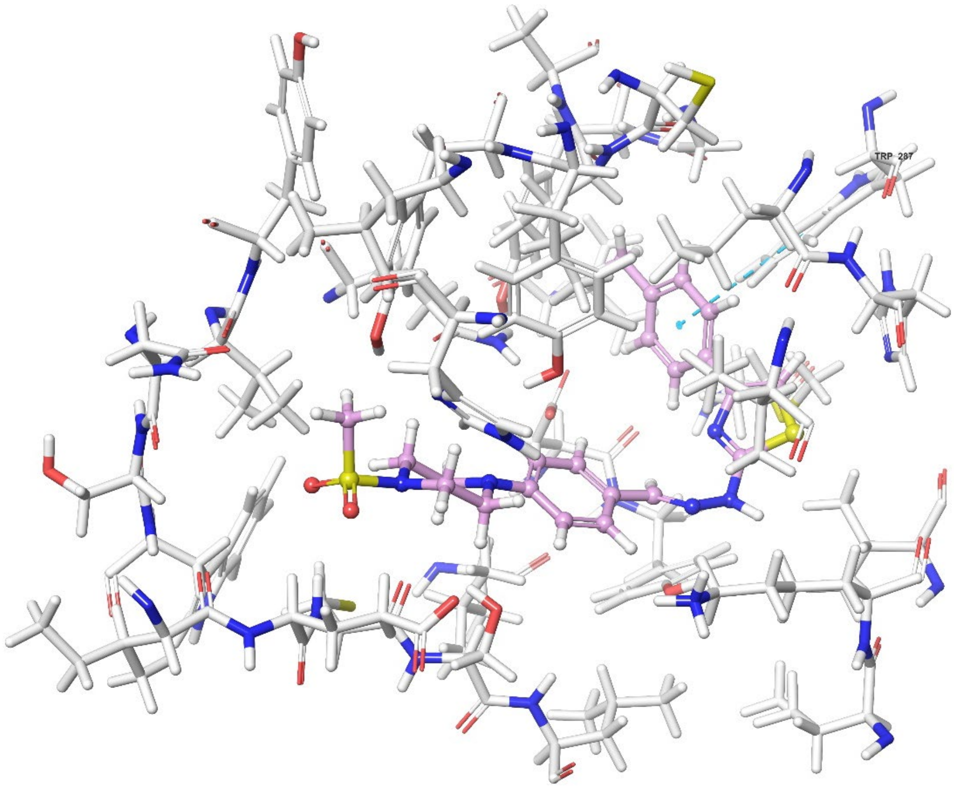

Figure S60. The three-dimensional interacting mode of compound **3b** in the active region of κ-opioid receptor. The ligand and significant residues of the active site of the receptor are presented by a tube model colored with pink and white, respectively (PDB Code: 6B73).

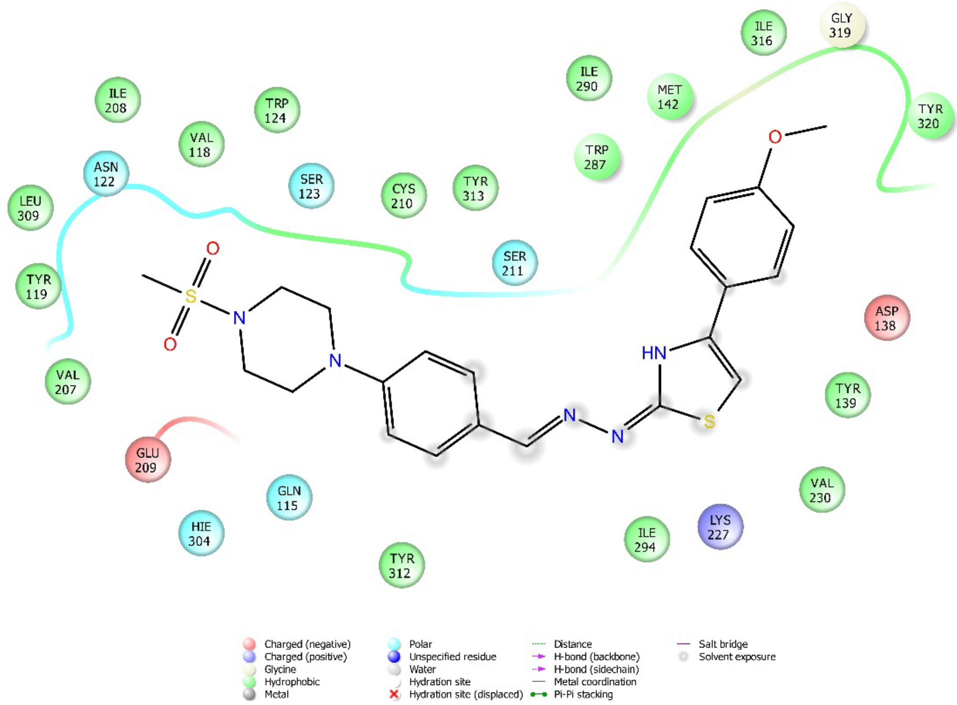

Figure S61. The two-dimensional interacting mode of compound **3c** in the active region of κ-opioid receptor (PDB Code: 6B73).

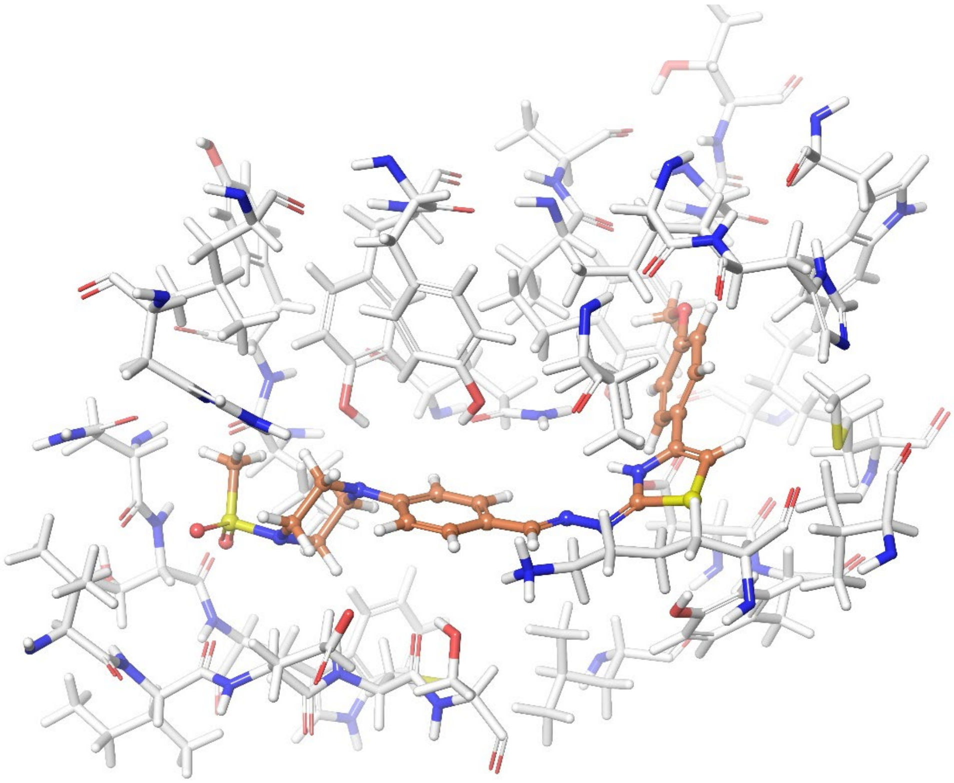

Figure S62. The three-dimensional interacting mode of compound **3c** in the active region of κ-opioid receptor. The ligand and significant residues of the active site of the receptor are presented by a tube model colored with orange and white, respectively (PDB Code: 6B73).

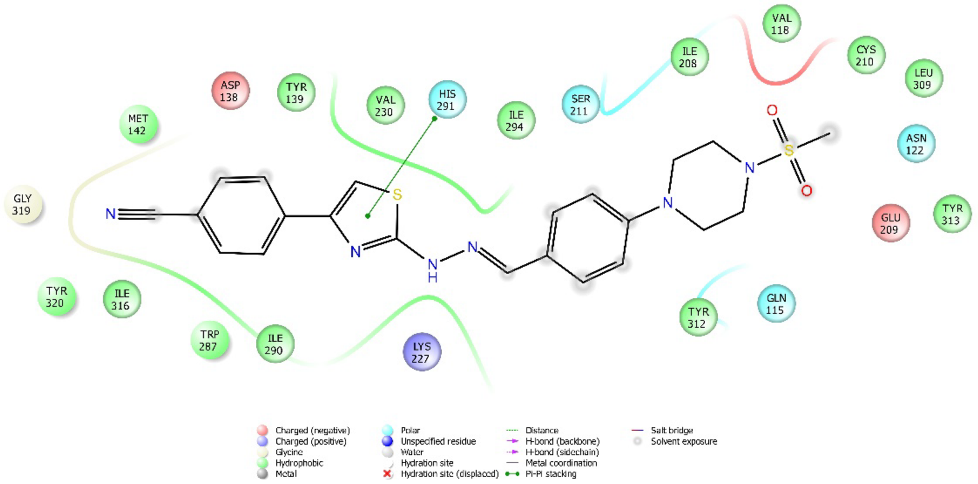

Figure S63. The two-dimensional interacting mode of compound **3d** in the active region of κ-opioid receptor (PDB Code: 6B73).

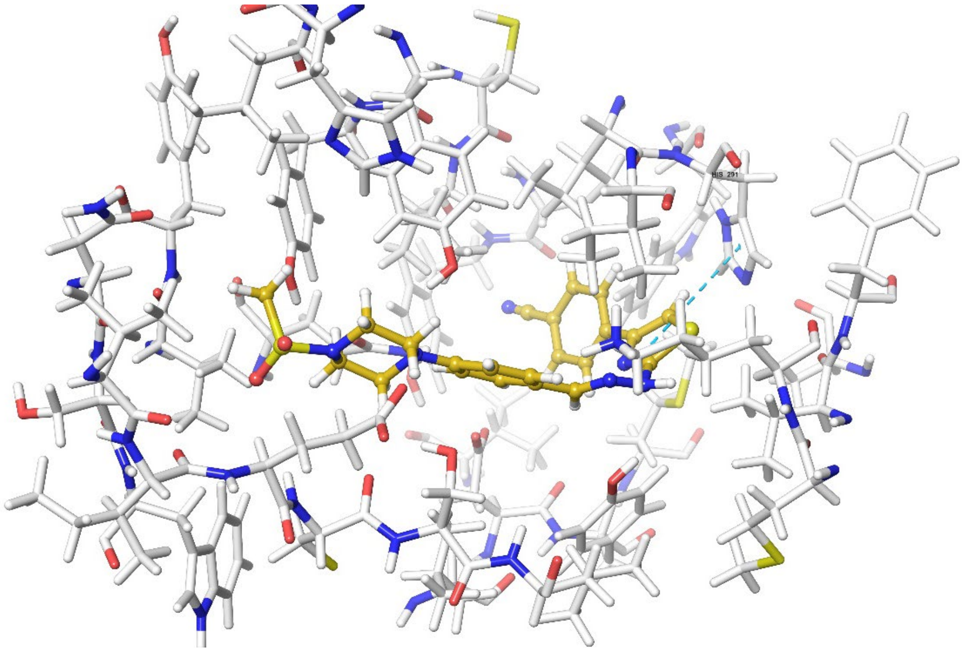

Figure S64. The three-dimensional interacting mode of compound **3d** in the active region of κ-opioid receptor. The ligand and significant residues of the active site of the receptor are presented by a tube model colored with yellow and white, respectively (PDB Code: 6B73).

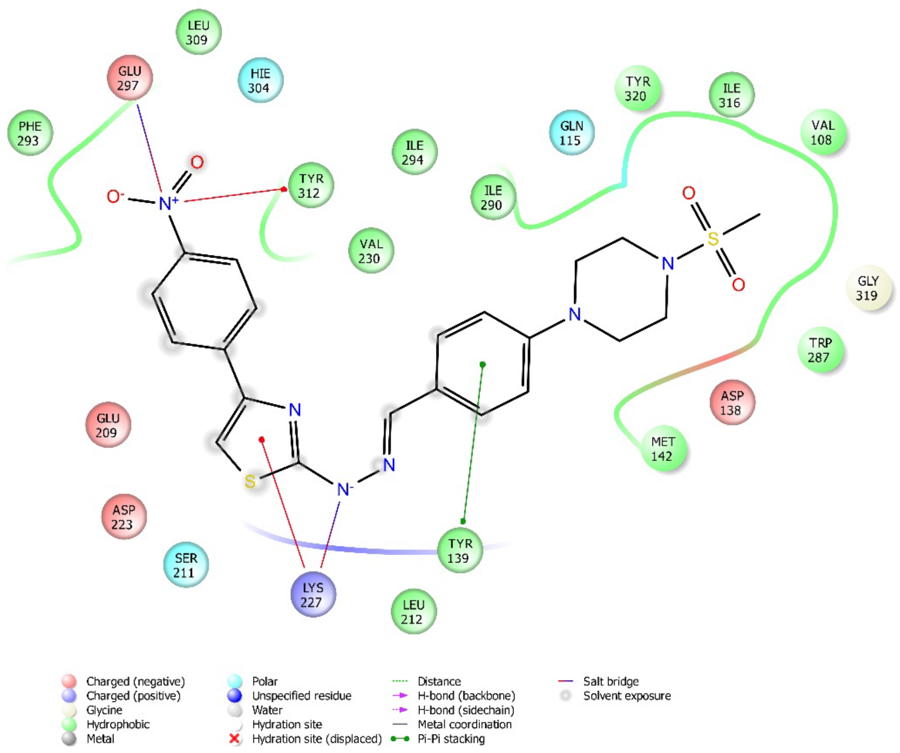

Figure S65. The two-dimensional interacting mode of compound **3e** in the active region of κ-opioid receptor (PDB Code: 6B73).

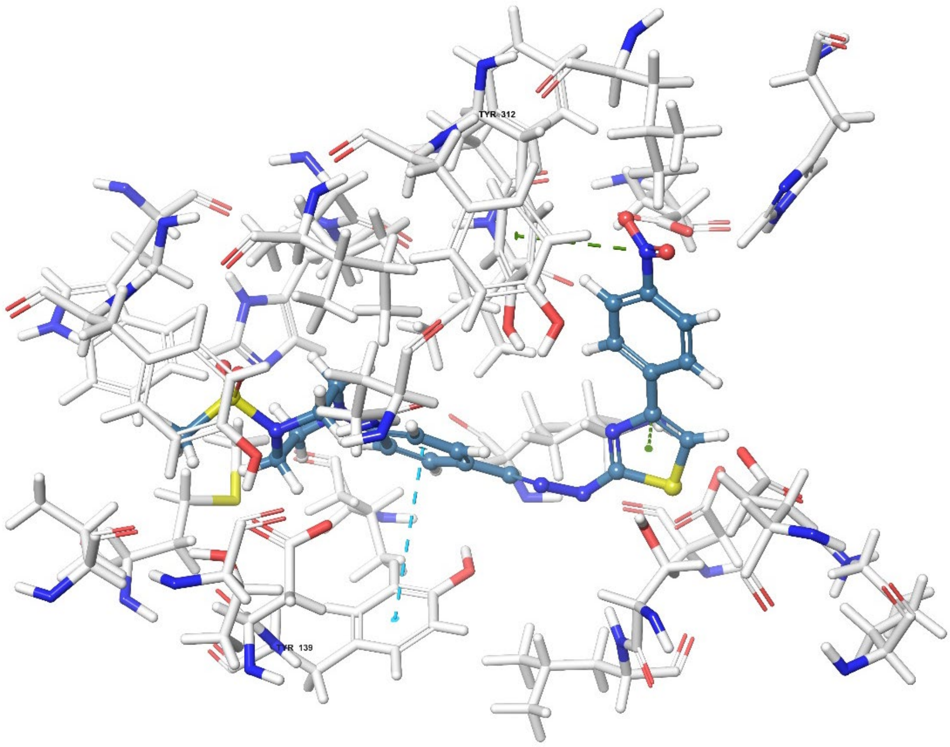

Figure S66. The three-dimensional interacting mode of compound **3e** in the active region of κ-opioid receptor. The ligand and significant residues of the active site of the receptor are presented by a tube model colored with blue and white, respectively (PDB Code: 6B73).

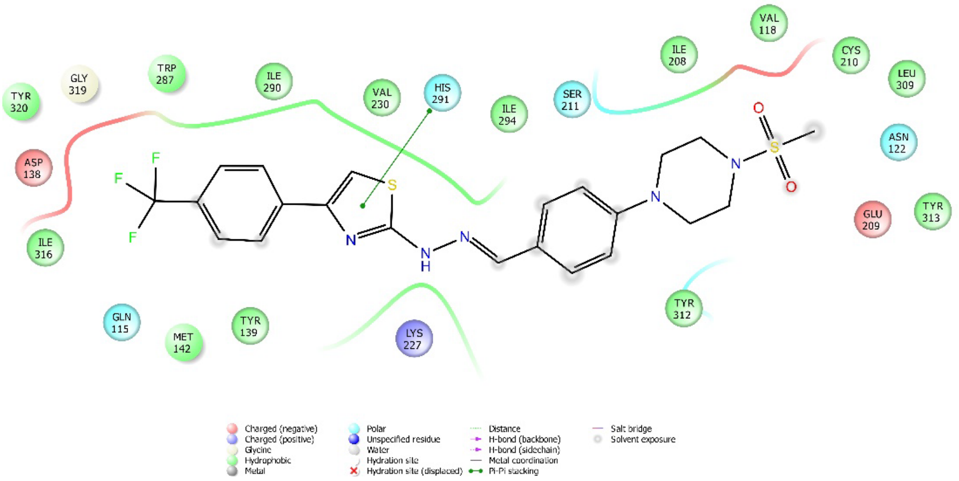

Figure S67. The two-dimensional interacting mode of compound **3h** in the active region of κ-opioid receptor (PDB Code: 6B73).

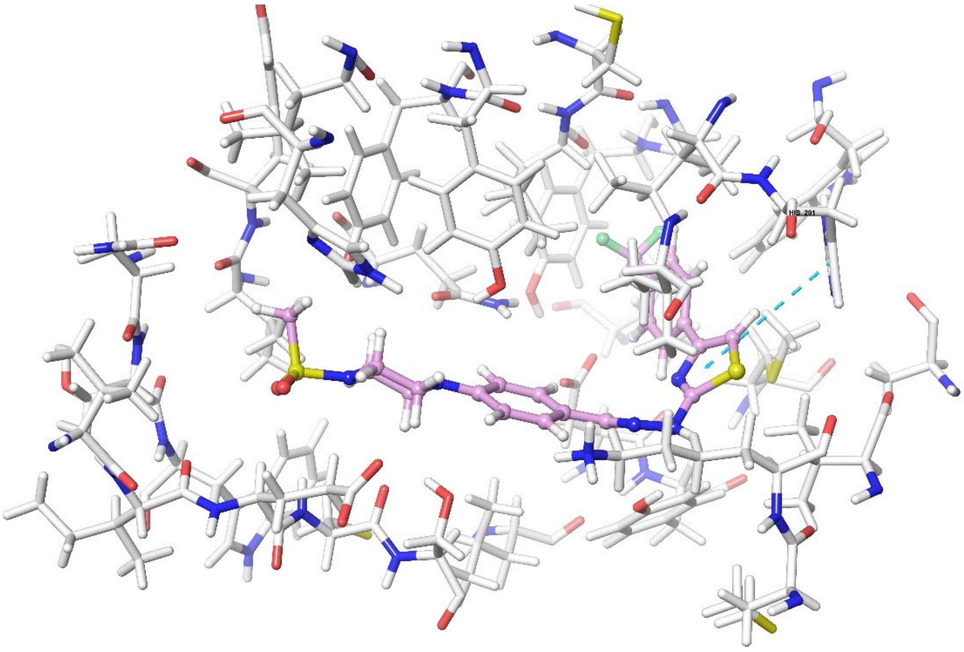

Figure S68. The three-dimensional interacting mode of compound **3h** in the active region of κ-opioid receptor. The ligand and significant residues of the active site of the receptor are presented by a tube model colored with pink and white, respectively (PDB Code: 6B73).
